# The molecular interaction of six single-stranded DNA aptamers to cardiac troponin I revealed by docking and molecular dynamics simulation

**DOI:** 10.1371/journal.pone.0302475

**Published:** 2024-05-15

**Authors:** Bejo Ropii, Maulidwina Bethasari, Isa Anshori, Allya Paramita Koesoema, Wervyan Shalannanda, Ardianto Satriawan, Casi Setianingsih, Mohammad Rizki Akbar, Reza Aditama, Fahmi Fahmi, Erwin Sutanto, Muhammad Yazid, Muhammad Aziz

**Affiliations:** 1 School of Electrical Engineering and Informatics, Bandung Institute of Technology, Bandung, West Java, Indonesia; 2 Department of Pharmacy, Universitas Muhammadiyah Bandung, Bandung, West Java, Indonesia; 3 Center for Health and Sports Technology, Bandung Institute of Technology, Bandung, West Java, Indonesia; 4 Department of Computer Engineering, School of Electrical Engineering, Telkom University, Bandung Regency, West Java, Indonesia; 5 Department of Cardiology and Vascular Medicine, Faculty of Medicine, Universitas Padjadjaran and Dr. Hasan Sadikin General Hospital, Bandung, Indonesia; 6 Biochemistry and Biomolecular Engineering Research Division, Faculty of Mathematics and Natural Sciences, Bandung Institute of Technology, Bandung, West Java, Indonesia; 7 Department of Electrical Engineering, Faculty of Engineering, Universitas Sumatera Utara, Medan, North Sumatera, Indonesia; 8 Department of Physics, Faculty of Science and Technology, Universitas Airlangga, Kampus C Unair Mulyorejo, Surabaya, East Java, Indonesia; 9 Biomedical Engineering Department, Institut Teknologi Sepuluh Nopember, Surabaya, East Java, Indonesia; 10 Institute of Industrial Science, The University of Tokyo, Tokyo, Japan; Qassim University, SAUDI ARABIA

## Abstract

Cardiac troponin I (cTnI) is a cardiac biomarker for diagnosing ischemic heart disease and acute myocardial infarction. Current biochemical assays use antibodies (Abs) due to their high specificity and sensitivity. However, there are some limitations, such as the high-cost production of Abs due to complex instruments, reagents, and steps; the variability of Abs quality from batch to batch; the low stability at high temperatures; and the difficulty of chemical modification. Aptamer overcomes the limitations of antibodies, such as relatively lower cost, high reproducibility, high stability, and ease of being chemically modified. Aptamers are three-dimensional architectures of single-stranded RNA or DNA that bind to targets such as proteins. Six aptamers (Tro1-Tro6) with higher binding affinity than an antibody have been identified, but the molecular interaction has not been studied. In this study, six DNA aptamers were modeled and docked to cTnI protein. Molecular docking revealed that the interaction between all aptamer and cTnI happened in the similar cTnI region. The interaction between aptamer and cTnI involved hydrophobic interaction, hydrogen bonds, *π*-cation interactions, *π*-stack interactions, and salt-bridge formation. The calculated binding energy of all complexes was negative, which means that the complex formation was thermodynamically favorable. The electrostatic energy term was the main driving force of the interaction between all aptamer and cTnI. This study could be used to predict the behavior of further modified aptamer to improve aptamer performance.

## Introduction

Cardiovascular diseases (CVDs), particularly stroke and ischemic heart disease, are the first two leading causes of death in Indonesia and worldwide [1–3]. Cardiac troponin (cTn) has been used as a cardiac biomarker for CVDs diagnosis, particularly for the suspected acute coronary syndrome (ACS). Prolonged ACS could lead to cardiac ischemia [4, 5]. Cardiac ischemia occurs when the heart tissue doesn’t get sufficient blood supply due to partial or total occlusion of a coronary artery due to plaque rupture. A prolonged episode of ischemia will lead to permanent myocardial damage and cause acute myocardial infarction (AMI). Cardiac troponins protein regulates the calcium-mediated interaction between thin and actin filaments of the cardiac muscle. Cardiac troponin T (cTnT) and troponin I (cTnI) are highly sensitive and specific markers of myocardial injury [6, 7]. cTnT and cTnI assay have become a clinically major assessment of patients with acute and chronic CVDs [8, 9] and are currently used as the gold standard for CVDs biomarkers. Current assay of cTnT and cTnI rely on detecting protein using monoclonal antibodies (MAbs) [10, 11]. The advantage of using MAbs is the high specificity and sensitivity of the assay. However, there are some limitations, such as the high-cost production of MAbs due to complex instruments, reagents, and steps; the variability of MAbs quality from batch to batch; the low stability at high temperatures; and the difficulty of chemical modification. Aptamer is a three-dimensional structure of single-stranded RNA or DNA that binds to targets such as proteins [12]. Aptamer, which specifically binds to cTnT and cTnI can be used as an alternative to antibodies for diagnostic application [13]. Aptamers overcome the limitation of MAbs, such as relatively lower cost, high reproducibility, high stability, and ease of being chemically modified [14, 15].

The biomolecule interactions, such as the interaction between the aptamer and target protein, result from its structure and dynamics. Molecular docking and molecular dynamics have been used to determine the complex conformation of aptamer and protein as well as its stability and dynamics [16–25]. DNA aptamers targetting cTnI has been isolated. in 2015, four aptamer sequences (TnIApt23, TnIApt19, TnIApt18, TnIApt11) were isolated by Dorraj et. al. [26]. Some aptamers have been applied for ELONA-type sandwich assay [27] and a sandwich bioluminescent assay for cTnI detection capable of detecting 0.04–3 nM of cTnI was developed [28–30]. Among these identified cTnI targetting aptamers, Six aptamers (Tro1-Tro6) which were identified by Jo et al., have been applied the most [13]. All of the 6 aptamers have distinct DNA sequences with KD values in the picomolar range for testing cTnI in serum. Tro4 and Tro6 have been proven with various proteins, including myoglobin and BSA. Tro4 has been used for numerous biosensors and detection methods [31]. Despite its high-applicabilities, The molecular interaction of these aptamers and cTnI have not been thoroughly investigated. In this study, we investigate the interaction of these six single-stranded DNA aptamers with cTnI using molecular docking and molecular dynamics approach.

## Methods

### Three-dimensional model preparation

Six DNA aptamer sequences (Table 1) were obtained from previous SELEX study [13] and its 2D and 3D structures were predicted using mFold [32] and RNAComposer web server, respectively [33, 34]. DNA aptamer sequences were initially transcribed into RNA sequences by replacing Thymine with Uracil. Subsequently, the RNA sequences were employed to construct 2D and 3D RNA aptamer structures using mFold and RNA composer, respectively. The resultant 3D RNA aptamer structures were then transformed back into DNA aptamers by substituting Uracil with Thymine through the use of Discovery Studio Visualizer. The DNA aptamers were then relaxed by performing 100 ns of MD simulation [35]. The three-dimensional structure of cTnI was modeled based on its amino acid sequence. To replicate a prior experimental study utilizing synthetic cTnI, the corresponding gene was identified through a BLAST search using the Troponin I primers: 5’-GCGGATCCATGGCGGATGGGAGCAG-3’ and 5’-GCAAGCTTTCAGCTCTCAAACTTTTTCTTGCGG-3’. The targeted gene was a Synthetic construct Homo sapiens clone CCSBHm_00030798 TNNI3 (see Table 1) [36]. The gene encoded the complete mRNA of cTnI. The mRNA was employed to derive the amino acid sequence, which was subsequently utilized to construct the complete 3D cTnI protein through the SWISS-MODEL tool [37]. Each structure was relaxed by 100ns of MD simulation and the final conformation was used for molecular docking. Relaxing the molecule before starting molecular docking is a critical step to establish a realistic and stable starting point, allowing subsequent simulations to represent the intended biological system accurately.

**Table 1:** The aptamer names and sequences used in this study. The nucleotides highlighted in both underlined and bold format were identified as unique, and it was predicted that these specific nucleotides play a crucial role in the interaction with the cTnI protein.

Name	Sequence
Tro1	5’-TCACACCCTCCC**TCCC**ACATACCGCATACAC**TTT**C**T**GATT-3’
Tro2	5’-CCCGACCACG**TCCC**TGCCCTTTCCTAACCTG**TTT**GTTGAT-3’
Tro3	5’-ATGCGTTGAA**CCCTC**CTGACCGTTTATCACATACTCCAGA-3’
Tro4	5’-CGTGCAGTACGCCAACC**TTT**C**TCA**TGCGCTGC**CCCTC**TTA-3’
Tro5	5’-CAACTGT**AA**TGTAC**CCTC**CTCGATCACGCACCACTTGCAT-3’
Tro6	5’-CGCATGCCA**AA**CGTTG**CCTC**ATAGTTCCCTCCCCGTGTCC-3’
cTnI	MADESGDAAG CPPPAPAPIR RQSSANYRAY ATEPHAKKKS
	KISASRKLQL KTLMLQIAKQ ELEREAEERR GEKGRALSTR
	CQPLELAGLG FAELQDLCRQ LHARVDKVDE ERYDVEAKVT
	KNITEIADLT QKIFDLRGKF KRPTLRRVRI SADAMMQALL
	GTRAKESLDL RAHLKQVKKE DTEKENREVG DWRKNIDALS
	GMEGRKKKFE

### Molecular docking

To obtain information about the possible binding location between aptamers and cTnI, molecular docking was performed using the HADDOCK web server, a docking approach based on biochemical or biophysical information [38, 39]. The relaxed 3D structure of cTnI and each aptamer were used as input molecules for receptor and ligand, respectively. DP-bind was used to determine the input of the active residues of the protein [40] while the active residues of each aptamer were obtained from previously predicted study [13], which were underlined and bold in Table 1. Default docking parameters 56 provided by HADDOCK were used to perform the molecular docking. for distance restraints, restraints were automatically generated to uphold the structural integrity, with the deletion of all hydrogens except those bonded to a polar atom (N, O). Ambiguous restraints were subject to random exclusion, employing a fraction of exclusions determined by the number of partitions (%excluded = 100/number of partitions), set at 2.0000. Rigid body docking involved the sampling of 1000 structures, and the subsequent minimization process consisted of 5 trials. During rigid body EM, solutions were explored through 180-degree rotation. Semi-flexible refinement was conducted with 200 structures, followed by a final refinement in the presence of water as a solvent. Fraction of Common Contacts (FCC) was used as the Clustering method for clustering parameters. RMSD Cutoff for clustering was set at 0.6A. a minimum cluster size of 4 was established. Energy and interaction parameters utilized OPLSX as nonbonded parameters, with electrostatics included during rigid body docking (it0 and it1). The epsilon constant for the electrostatic energy term in it0 was set at 10.0, while in it1, it was set at 1.0. Cutoff distances for defining hydrogen bonds and hydrophobic contacts were set at 2.5 and 3.9 (carbon-carbon), respectively.

### Molecular dynamics simulation

MD simulation was performed using GROMACS and CHARMM36 force field [41, 42]. The complex of the aptamer-cTnI was selected from the best (lowest-energy, best Haddock score) configuration of the molecular docking scores. In each system, the Tro-cTnI complex was immersed in a TIP3P water model, enclosed within a dodecahedron box positioned 1 nm away from the complex’s edges. To achieve neutrality, the system was balanced by incorporating the requisite number of ions, either sodium or chloride. Periodic boundary conditions (PBCs) were applied to the system in all the spatial directions. LINCS algorithms were used, and all hydrogen bonds were constrained. A 1.2 nm distance cutoff for the short-distance electrostatic and van der Waals interaction was used. Particle Mesh Ewald algorithm (PME) was used to calculate the long-range electrostatic forces. The steepest descent algorithm was used to minimize the system’s energy. The system was then allowed to reach an equilibrium state through the NVT ensemble using the V-Rescale thermostat at 300K, then through NPT ensemble using the Parrinello-Rahman barostat at 1 atm.

### Binding energy calculation

Molecular mechanics/Poisson–Boltzmann (Generalized-Born) surface area, also known as MM/PB(GB)SA, was used to calculate the Gibbs free binding energy between each aptamer-cTnI complex [43]. This approach is particularly applied in the field of computational chemistry and structural biology to study the thermodynamics of molecular interactions, such as protein-ligand binding. The MMPB(GB)SA method combines molecular mechanics, Poisson-Boltzmann electrostatics, and solvent-accessible surface area calculations to provide an estimate of the free energy changes associated with the binding of molecules. It can be used to analyze the energetics of protein-protein interactions, protein-ligand binding, or other biomolecular associations. MM/PB(GB)SA is a post-processing end-state method to calculate the free energies of molecules in solution [44]. The Gibbs free binding energy (Δ*G*_*binding*_) was a difference between energy in complex and energy in every single structure of receptor or ligand.

$$\begin{array}{c} \Delta G_{binding}=\Delta G_{Tro-cTnlcomplex}-\left(\Delta G_{Tro}+\Delta G_{cTnl}\right) \end{array}$$ (1)

The Δ*G* consist of polar (Δ*G*_*polar*_) and non-polar (Δ*G*_*non*–*polar*_) energy

$$\begin{array}{c} \Delta G=\Delta G_{polar}+\Delta G_{non-polar} \end{array}$$ (2)

Where Δ*G*_*polar*_ and Δ*G*_*non*–*polar*_ are given by

$$\begin{array}{c} \Delta G_{polar}=\Delta G_{ps}+\Delta G_{elec}\Delta G_{non-polar}=\Delta G_{nps}+\Delta G_{vdW} \end{array}$$ (3)

where Δ*Gps* and Δ*Gnps* are the difference of the polar and nonpolar solvation energies, respectively. Also, Δ*GvdW* is the energy difference related to the van der Waals interactions, and Δ*G*_*elec*_ stands for the energy difference associated with the electrostatic interactions.

## Results and discussion

### The structure and dynamics of individual aptamer and cTnI protein

**Fig 1:**
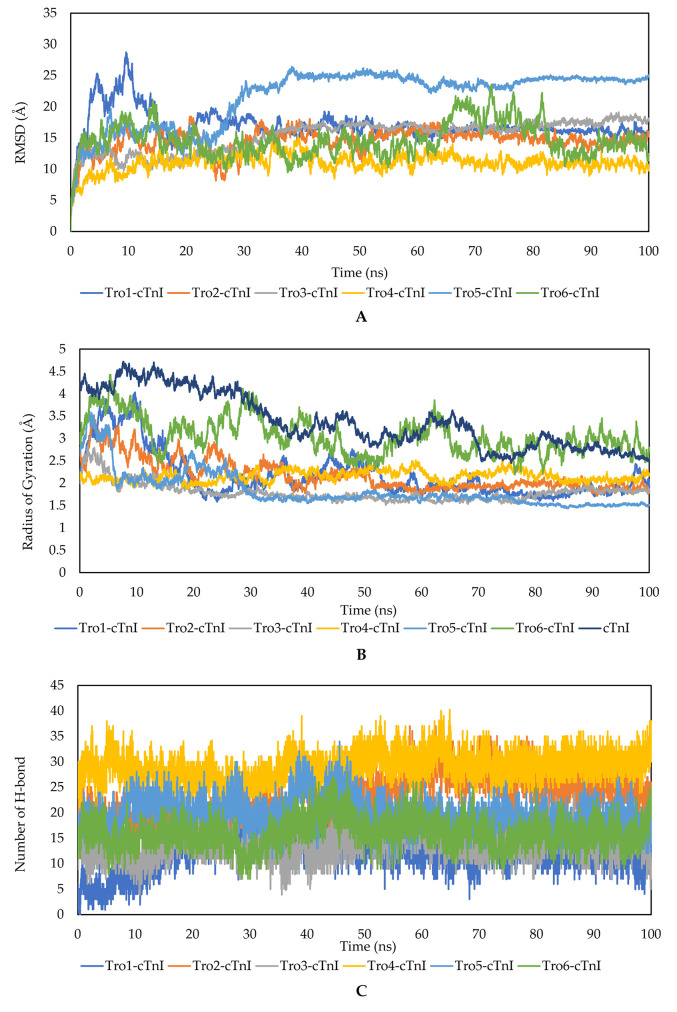
RMSD (A), Radius of Gyration (B), and Number of H-bonds (C) of each aptamer and cTnI during 100 ns MD simulation for structure relaxation. These structures were more relaxed and compact as shown by the RMSD and radius of gyration, respectively.

The 3D structure of aptamers and cTnI were obtained from RNAComposer modeling and the SWISS-MODEL, respectively. The initial structures were then relaxed using 100 ns MD simulation to better predict the individual structure in the water solution model. Fig 1 showed the root mean square distance (RMSD), Radius of Gyration (RoG), and Number of Hydrogen Bonds of each individual aptamer and cTnI protein during 100 ns of MD simulation for structure relaxation.

RMSD values showed that all aptamers and cTnI started to be stable at 85 ns during 100 ns simulation. The radius of gyration of all structure were observed to be stable at 80 ns (except for Tro6 which was slightly fluctuative). From these data, Tro4 occured to be the most stable aptamer, which was shown by the lowest RMSD value. Hydrogen bonds may play important role in the stabilization of aptamer structure. The average hydrogen bonds present in the Tro1-Tro6 were 12, 23, 14, 28, 15, and 19, respectively. For the cTnI system, during 100 ns of simulation, cTnI started to be stable at 40 ns. The structure of cTnI became more compact during the relaxation as shown by the radius of gyration (Fig 1).

**Fig 2:**
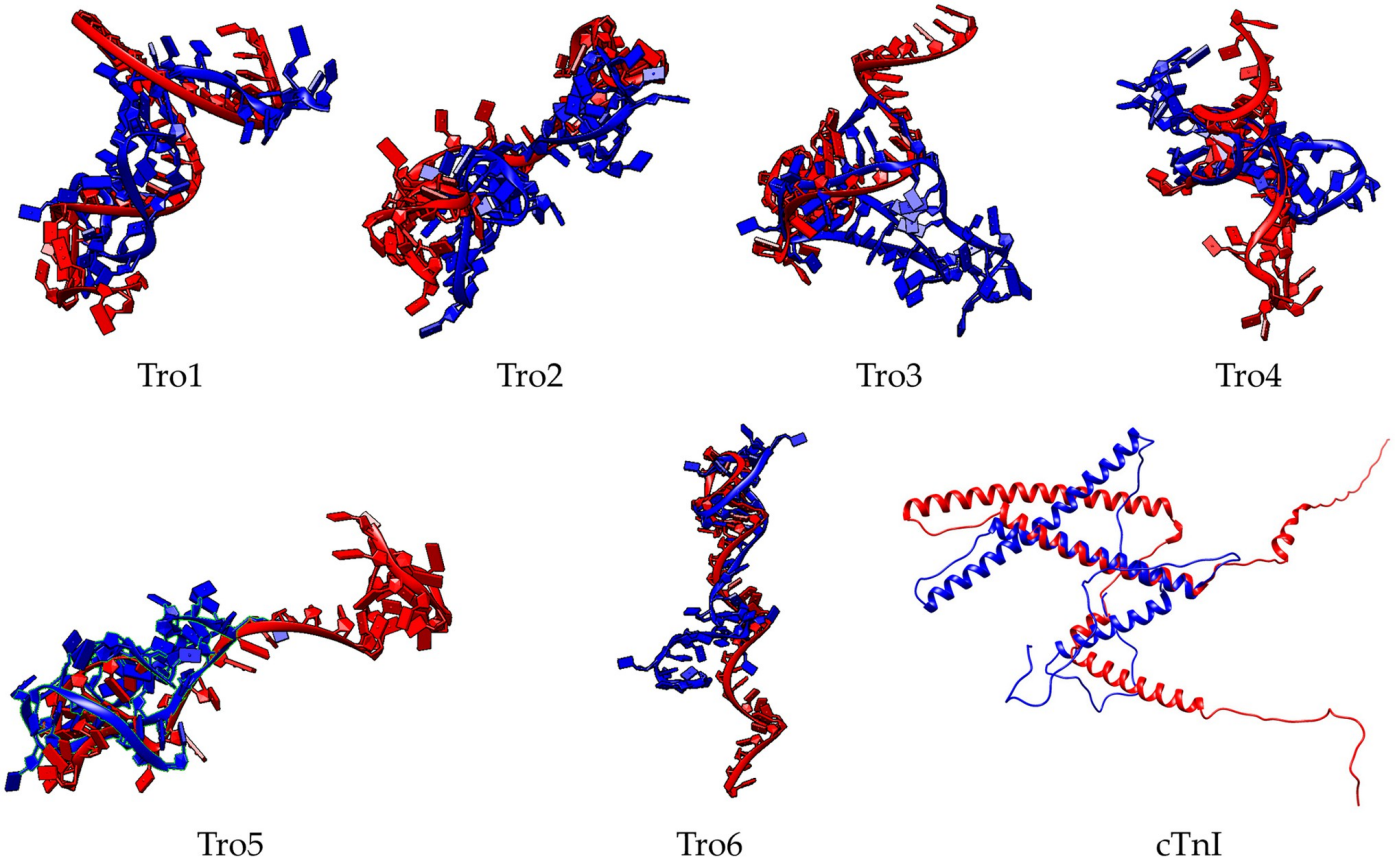
Structure comparison of aptamers and cTnI before (red) and after (blue) 100 ns MD simulation for structure relaxation. This blue visualization showed these structures become more compact after structure relaxation by 100 ns of MD simulation compared to the red.

The structure comparison of aptamers and cTnI protein before and after structure relaxation through MD simulation was shown in Fig 2. For aptamers, Tro1 and Tro5 structures were significantly changed from the initial structure, while Tro2, Tro3, Tro4, and Tro6 slightly became less compact. The visualization of cTnI before and after relaxation was more compact in agreement with the radius of gyration(Fig 1).

### Aptamer-cTnl docking

**Fig 3:**
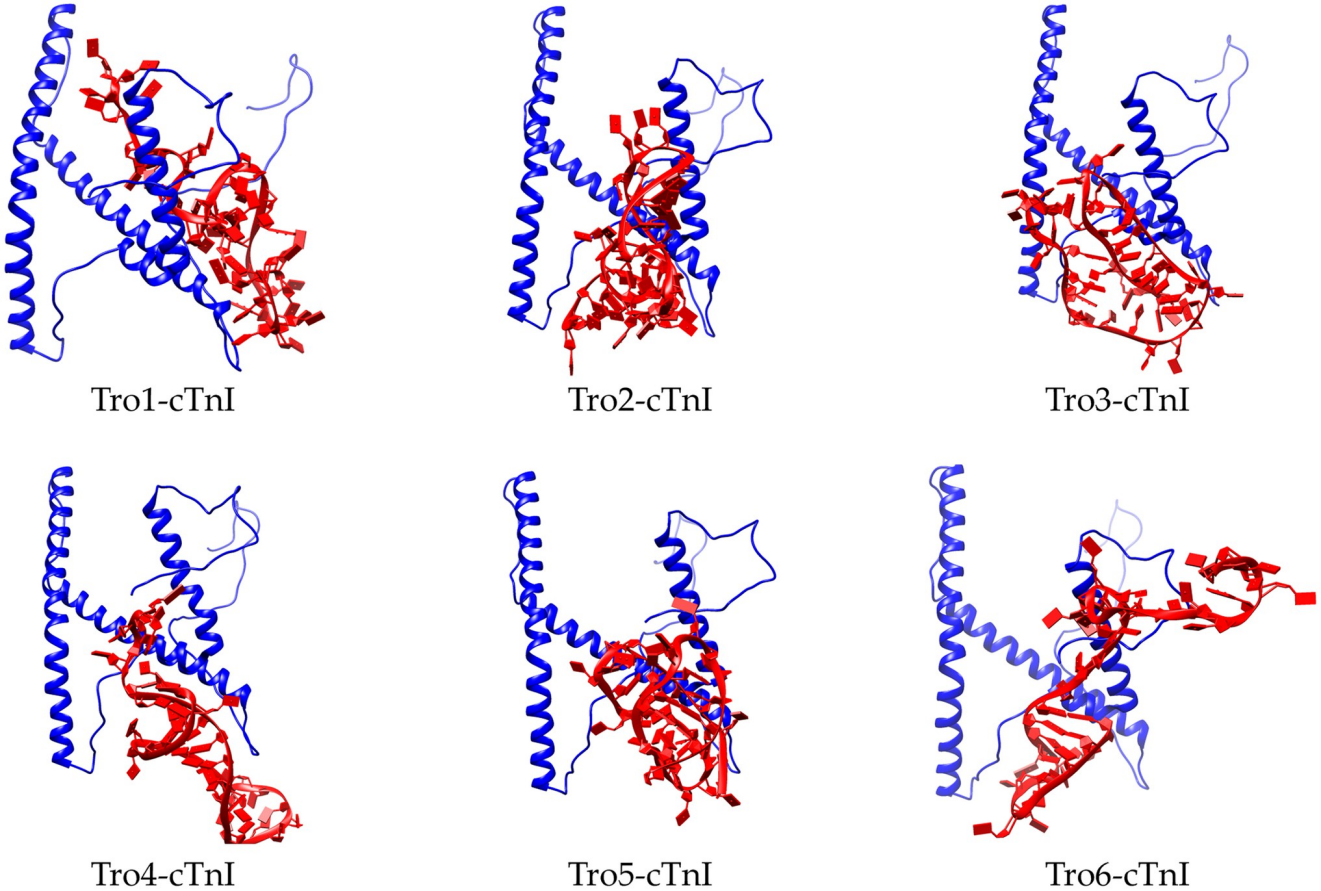
Predicted binding mode of aptamer-cTnI using HADDOCK. All aptamers interacted with similar regions of cTnI.

After 3D structure relaxation using 100 ns of MD simulation, molecular docking was performed using HADDOCK. cTnI protein and aptamer were used as the receptor and ligand input, respectively. The predicted active residues of protein were determined by DP-bind [40] which were Ala19, Arg22, Ser23, Lys37, Lys38, Lys40, Arg45, Lys46, Gln48, Lys50, Leu53, Gln55, Ile56, Ala57, Lys72, Arg79, Arg98, Lys120, Arg136, Gly137, Lys138, Phe139, Lys140, Arg141, Pro142, Thr143, Leu144, Arg145, Arg146, Val147, Arg148, Ile149, Ser150, Ala151, Arg170, His172, Lys174, Gln175, Val176, Lys177, Gly189, Trp191, Arg192, Lys193, Asn194, Gly203, Arg204, Lys205, Lys206, Lys207, and Phe208. The predicted active residues of aptamer were highlighted in both underlined and bold format in Table 1 [13]. HADDOCK utilizes data from interacting residues to guide the docking procedure. The active residues exhibit notable chemical shift perturbation upon complex formation and substantial solvent accessibility in the protein’s free form. Following the computation, the structures are sorted based on their intermolecular energy, encompassing the cumulative values of electrostatic, van der Waals, and ambiguous interaction restraints (AIR) energy terms. The HADDOCK output consists of several structures with different docking values. Structure with the lowest docking score was chosen for the next analysis. The docking score showed that all aptamers bind to the similar region of cTnI which suggests that the region play important role in binding with aptamer (Fig 3).

### Structural and interaction of aptamer-cTnI complexes through MD simulation

**Fig 4:**
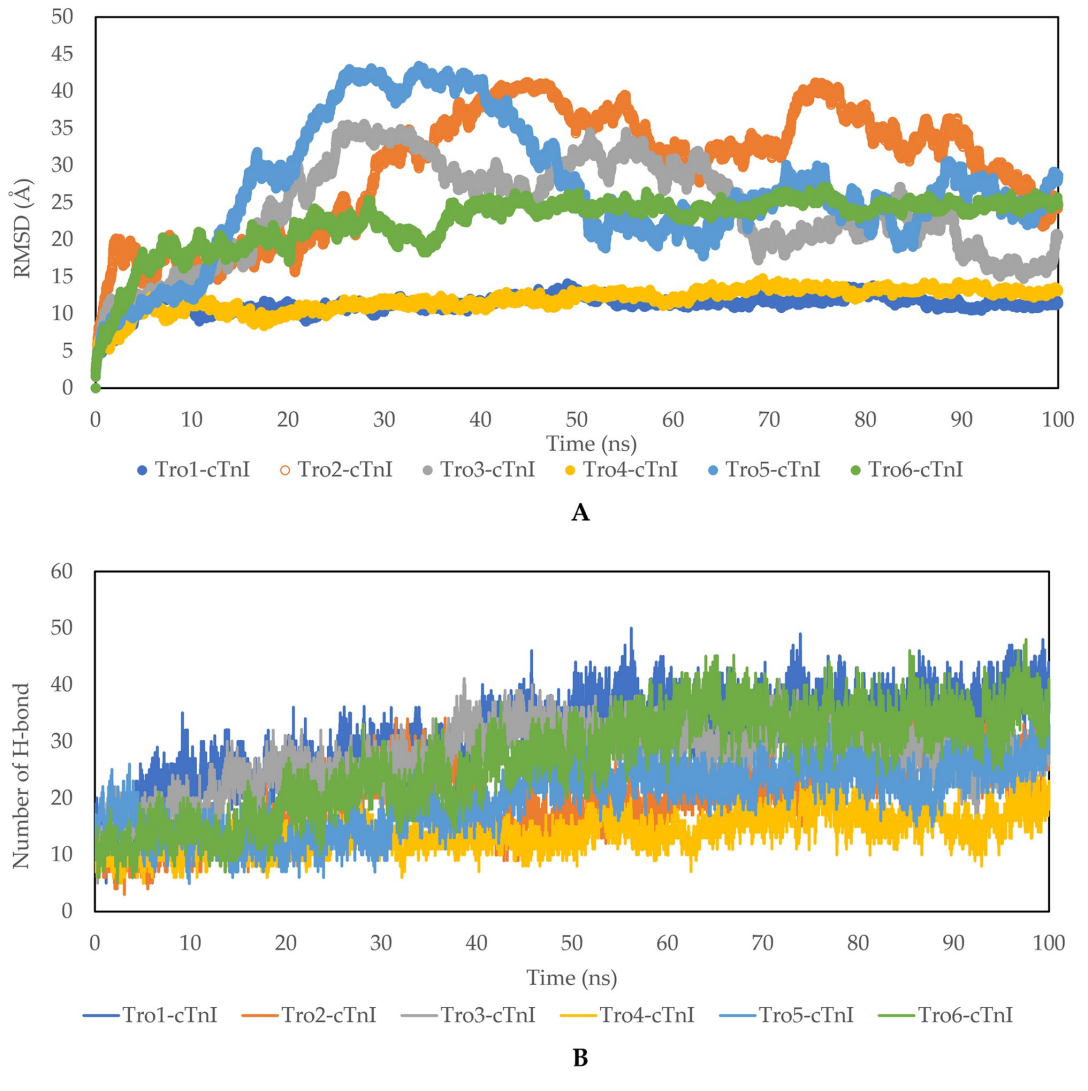
(a) RMSD, (b) number of Hydrogen bond of aptamer-cTnI complex during 100 ns MD simulation.

Aptamer-cTnI structure from the lowest docking score was then run for another 100 ns MD simulation to analyze the structural stability and dynamics between aptamer and cTnI. The RMSD and number of hydrogen bonds between aptamer and cTnI were calculated to evaluate the stability of the complex. Based on the RMSD value during the 100 ns MD simulation (Fig 4), the Tro1-cTnI and Tro4-cTnI complexes demonstrated stability starting from 10 ns, with the Tro6-cTnI complex stabilizing at 39 ns. In contrast, the remaining complexes exhibited relatively higher fluctuations and only began to show stability around 95 ns. Tro4-cTnI and Tro1-cTnI complex were relatively more stable than the others as shown by lower RMSD value. There were an increasing number of hydrogen bonds in all aptamer during the 100 ns of MD simulation.

To identify the molecular interaction of the aptamer-cTnI complex during the 100 ns simulation, a snapshot was taken at 0, 20, 40, 60, 80, and 100 ns from the trajectory. Protein-Ligan Interaction Profile (PLIP) was used to identify the residue that plays a role in the interaction between aptamer and cTnI for each snapshot [45]. PLIP analysis revealed that the interaction between aptamers and cTnI involved hydrophobic interaction, hydrogen bond, *π*-cation interactions, salt-bridge formation, and *π*-stacking interaction (S1 File). Our study was consistent with the literature. Studies showed that the formation of complexes between aptamers and proteins involves multiple interaction forces. *π*-cation interactions, where positively charged ions interact with aromatic rings, hydrogen bonds formed between specific atoms in the aptamer and protein, and electrostatic interactions between positively and negatively charged regions on both molecules collectively contribute to the stability and specificity of the complex. These diverse forces play a pivotal role in shaping the binding affinity between aptamers and proteins [46].

According to PLIP analysis, Table C.1. in S3 File presents the protein residues involved in interactions with all aptamers. The primary residues demonstrating consistent interaction across all aptamers were Arginine and Lysine, both of which fall within the category of positively charged amino acids. Additionally, Serine and Valine were identified as significant contributors to the major interacting residues observed in all aptamers.

Based on the PLIP analysis from six snapshots of each aptamer during 100ns of MD simulation, the interaction patterns of all aptamers with the protein revealed consistent binding to similar regions, as illustrated in Fig 3 and detailed in Table C.1. in S3 File. All aptamers interacted with residue Lys37, Arg141, and Arg148. Four cTnI residues that had more than fifty molecular interactions were Arg146, Lys37, Arg204, and Arg148, with the number of interactions were 85, 69, 68, and 53, respectively. Notably, the cTnI protein encompasses residues 1–33, distinguishing it from muscular troponin. Tro1 has strong interaction with Arg20 and Arg21. Tro2 did not interact with any residue number 1–33, which suggests that Tro2 has less selectivity compared to other aptamers. Tro3, Tro4, and Tro5 mainly interact with Arg21, Arg10, or Arg20, respectively. In addition, Tro6 mainly interacts with Arg21 and Arg22. This observation underscores the aptamer’s pronounced specificity for cTnI, implying its potential utility in targeted applications. During AMI, cTnI is represented in a number of diverse forms, of which the binary complex of TnC–cTnI is the predominant circulating form, with minor forms of the tertiary complex (TnC–cTnI–cTnT) and free cTnI. To this end, the mid-fragment of cTnI (amino acid residue 30–110) is recommended for detection due to its stability [47].

Compared to antibodies, The type of molecular interaction is similar to antibodies, such as Electrostatic interactions, hydrogen bonds, van der Waals forces, and hydrophobic interactions. [48, 49]. Therefore, these aptamers exhibit promising potential for applications in the detection of cTnI as an alternative for antibody, with the prospect of achieving high specificity.

**Fig 5:**
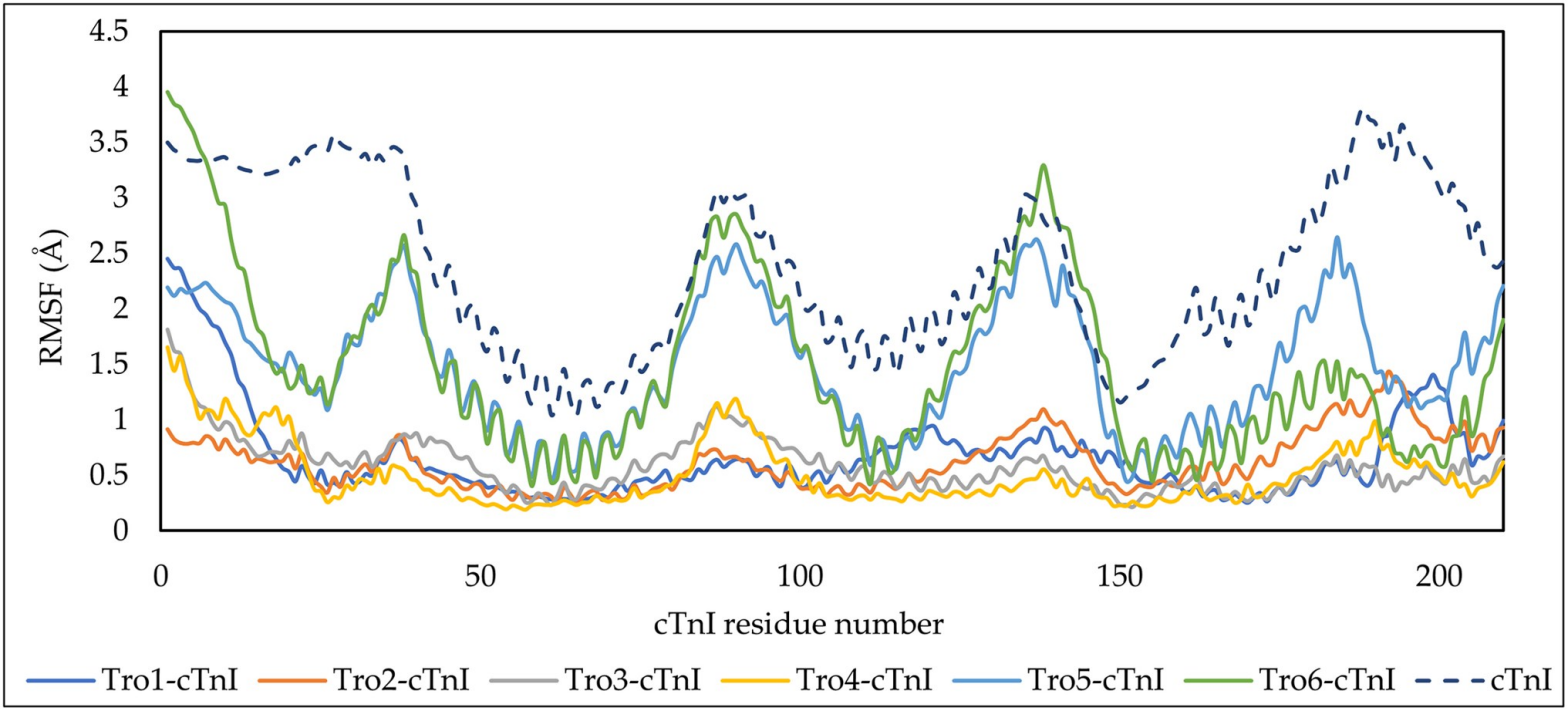
RMSF of individual cTnI and cTnI-in-aptamer-cTnI-complex.

To identify the effect of aptamer binding to cTnI, another 100 ns of cTnI MD simulation was performed from the previously relaxed cTnI structure. The RMSF of the individual cTnI was calculated and compared to the RMSF of cTnI in the aptamer-cTnI complex (Fig 5). The RMSF graph reveals significant fluctuations in residues exhibiting coil-shaped secondary structures within the cTnI protein. The RMSF of the cTnI from individual simulation (purple line) showed a higher value than cTnI in complex formation. This coil structure emerges as the primary source of fluctuations in the overall cTnI protein structure. However, a noteworthy observation arises when the cTnI interacts with the aptamer. This interaction induced stabilization to the general structure of cTnI, with a particular emphasis on mitigating fluctuations within the coil structure.

### Binding energy

The binding energy was calculated using the MMPB(GB)SA method. The calculated binding energy using MMPB(GB)SA is presented in Table 2. Calculated binding energy using the PB approach showed that Tro4 has the lowest binding energy, which agreed with the experimental result [13]. The PB and GB methods are continuum-solvation models that are used to calculate the free energy of binding of small ligands to biological macromolecules. These methods are based on molecular dynamics simulations of the receptor-ligand complex and are intermediate in both accuracy and computational effort between empirical scoring and strict alchemical perturbation methods. The computed binding energies consistently yielded negative values, demonstrating a congruence in results between the Poisson-Boltzmann (PB) and Generalized Born (GB) approaches. The detailed breakdown of the energy terms in the calculated PB and GB methods can be found in S2 File. Notably, the electrostatic energy emerged as the predominant driving force behind the interaction between the aptamer and cTnI protein.

**Table 2:** Calculated binding energy using MMPB(GB)SA.

Complex	ΔG exp (kcal/mol)	GB (kcal/mol)	PB (kcal/mol)
Tro1-cTnI	-12.996	-132.20	-160.63
Tro2-cTnI	-13.654	-21.14	-20.57
Tro3-cTnI	-13.649	-22.83	-21.89
Tro4-cTnI	-14.508	-202.47	-236.13
Tro5-cTnI	-13.024	-19.04	-17.61
Tro6-cTnI	-14.412	-113.89	-125.41

## Conclusions

We conducted molecular docking and molecular dynamics simulations for six DNA aptamers binding to the cTnI protein to elucidate their molecular interactions. The stability of each aptamer was assessed using the RMSD value, revealing that Tro4 exhibited the highest stability, as indicated by the lowest RMSD value throughout the simulation. Hydrogen bonds appeared to contribute significantly to the stability of the aptamers. The stability of aptamer-cTnI interactions was further confirmed by RMSD values, indicating sustained binding during the 100ns MD simulation. All aptamers demonstrated binding to a similar region of cTnI, including residues specific to this protein. This observation suggested that Tro1 and Tro4 may have relatively higher selectivity due to their binding with the specific region of cTnI (residue 1–33). The molecular interactions involved hydrophobic interactions, hydrogen bonds, *π*-stack, *π*-cation interactions, and salt-bridge formation. MMPB(GB)SA calculations yielded negative values, signifying favorable interactions, with electrostatic energy identified as the primary driving force. Notably, our study revealed that aptamers exhibit molecular interactions similar to antibodies but with stronger binding affinities, as demonstrated in a previous study. Consequently, these developed aptamers present themselves as potential substitutes for anti-cTnI antibodies, offering enhanced sensitivity for acute myocardial infarction (AMI) diagnosis. The results of this in-silico analysis can further explain the in-vitro results of the original experiments by Jo. Further development of this in-silico analysis can be applied to improve aptamer stability as one of the methods to enhance aptamer stability is by nucleic acid structure nucleic acid.

## Supporting information

S1 File Protein-ligand interaction profiler results. (including a table of atom types in Chimera [50]).
(PDF)

S2 File Energy terms. (PDF)

S3 File The number of molecular interactions of all aptamers with the protein. (PDF)
