# Supplementary material for: The molecular interaction of six single-stranded DNA aptamers to cardiac troponin I revealed by docking and molecular dynamics simulation
Source: PLoS One. 2024 May 15;19(5):e0302475. doi: 10.1371/journal.pone.0302475 (PMC11095691; doi:10.1371/journal.pone.0302475)
Supplement: S1 File — (including a table of atom types in Chimera [50]). (PDF) [file pone.0302475.s001.pdf]

Table A.1. Hydrophobic Interaction

Complex_Time point	Index	Residue	AA	Distance	Ligand Atom	Protein Atom
Tro1-cTnI20	1	20A	ARG	3.9	2111	136
Tro1-cTnI20	2	36A	LYS	3.96	2331	272
Tro1-cTnI20	3	36A	LYS	3.56	2273	273
Tro1-cTnI20	4	52A	LEU	3.21	2071	401
Tro1-cTnI20	5	55A	GLN	3.89	1946	424
Tro1-cTnI20	6	100A	LEU	3.83	1697	782
Tro1-cTnI20	7	165A	GLU	3.4	1965	1306
Tro1-cTnI40	1	18A	PRO	3.91	2105	121
Tro1-cTnI40	2	20A	ARG	3.63	2111	136
Tro1-cTnI40	3	36A	LYS	3.81	2273	271
Tro1-cTnI40	4	93A	LEU	3.86	1697	724
Tro1-cTnI40	5	162A	ARG	3.73	1952	1281
Tro1-cTnI40	6	162A	ARG	3.81	1984	1282
Tro1-cTnI40	7	165A	GLU	3.84	1965	1307
Tro1-cTnI40	8	170A	ARG	3.87	1894	1346
Tro1-cTnI40	9	206A	LYS	3.88	1971	1644
Tro1-cTnI60	1	18A	PRO	4	2105	121
Tro1-cTnI60	2	20A	ARG	4	2111	136
Tro1-cTnI60	3	36A	LYS	3.79	2331	273
Tro1-cTnI60	4	36A	LYS	3.97	2273	271
Tro1-cTnI60	5	52A	LEU	3.46	2069	402
Tro1-cTnI60	6	100A	LEU	3.78	1712	782
Tro1-cTnI60	7	170A	ARG	3.8	1894	1346
Tro1-cTnI60	8	206A	LYS	3.78	1971	1642
Tro1-cTnI80	1	36A	LYS	3.7	2273	272
Tro1-cTnI80	2	55A	GLN	3.64	1931	423
Tro1-cTnI80	3	100A	LEU	3.54	1712	783
Tro1-cTnI80	4	162A	ARG	3.72	1952	1281
Tro1-cTnI80	5	165A	GLU	3.69	1965	1306
Tro1-cTnI80	6	169A	LEU	3.82	1971	1340
Tro1-cTnI100	1	36A	LYS	3.84	2273	271
Tro1-cTnI100	2	52A	LEU	3.64	2071	402
Tro1-cTnI100	3	55A	GLN	3.76	1931	423
Tro1-cTnI100	4	59A	GLN	3.67	1932	455
Tro1-cTnI100	5	162A	ARG	3.91	1952	1281
Tro1-cTnI100	6	162A	ARG	3.61	1984	1282
Tro1-cTnI100	7	173A	LEU	3.95	1888	1373
Tro1-cTnI0	1	36A	LYS	3.38	3147	343
Tro1-cTnI0	2	45A	ARG	3.61	3213	434
Tro1-cTnI0	3	52A	LEU	3.8	2744	517
Tro1-cTnI0	4	98A	ARG	3.79	2156	965
Tro2-cTnI0	1	149A	ILE	3.78	2311	1520

Complex_Time point	Index	Residue	AA	Distance	Ligand Atom	Protein Atom
Tro2-cTnI0	2	157A	ALA	3.3	2560	1586
Tro2-cTnI0	3	204A	ARG	3.76	3188	2054
Tro2-cTnI20	1	35A	ALA	3.71	1817	266
Tro2-cTnI20	2	47A	LEU	3.49	2049	361
Tro2-cTnI20	3	47A	LEU	3.72	1975	358
Tro2-cTnI40	1	35A	ALA	3.87	1817	266
Tro2-cTnI40	2	43A	ALA	3.76	1976	327
Tro2-cTnI40	3	47A	LEU	3.87	2049	361
Tro2-cTnI40	4	47A	LEU	3.69	1975	360
Tro2-cTnI40	5	147A	VAL	3.86	1912	1177
Tro2-cTnI40	6	149A	ILE	3.82	1931	1195
Tro2-cTnI60	1	158A	LEU	3.93	1975	1258
Tro2-cTnI80	1	43A	ALA	3.97	1976	327
Tro2-cTnI80	2	144A	LEU	3.6	1912	1149
Tro2-cTnI80	3	146A	ARG	3.9	1792	1166
Tro2-cTnI100	1	47A	LEU	3.67	2049	360
Tro2-cTnI100	2	47A	LEU	4	1974	359
Tro2-cTnI100	3	146A	ARG	3.87	1792	1166
Tro2-cTnI100	4	158A	LEU	3.79	1975	1259
Tro2-cTnI100	5	207A	LYS	3.96	2333	1651
Tro2-cTnI100	6	209A	GLU	3.71	2313	1672
Tro3-cTnI20	1	38A	LYS	3.77	2020	289
Tro3-cTnI20	2	140A	LYS	3.53	2467	1114
Tro3-cTnI20	3	174A	LYS	3.96	2239	1380
Tro3-cTnI40	1	38A	LYS	3.76	2021	289
Tro3-cTnI40	2	112A	TYR	3.45	2306	889
Tro3-cTnI40	3	141A	ARG	3.77	1919	1122
Tro3-cTnI40	4	149A	ILE	3.72	2399	1195
Tro3-cTnI40	5	174A	LYS	3.51	2239	1379
Tro3-cTnI40	6	191A	TRP	3.83	2245	1526
Tro3-cTnI40	7	208A	PHE	3.83	2239	1664
Tro3-cTnI60	1	38A	LYS	3.62	2020	289
Tro3-cTnI60	2	131A	LYS	3.59	1755	1035
Tro3-cTnI60	3	149A	ILE	3.6	2399	1196
Tro3-cTnI60	4	167A	LEU	3.62	2206	1323
Tro3-cTnI60	5	174A	LYS	3.46	2239	1380
Tro3-cTnI60	6	177A	LYS	3.53	2239	1405
Tro3-cTnI60	7	191A	TRP	3.21	2245	1526
Tro3-cTnI80	1	38A	LYS	3.57	2021	290
Tro3-cTnI80	2	38A	LYS	3.64	2020	289
Tro3-cTnI80	3	112A	TYR	3.59	2306	889
Tro3-cTnI80	4	142A	PRO	3.9	1906	1134
Tro3-cTnI80	5	146A	ARG	3.74	2360	1165
Tro3-cTnI80	6	174A	LYS	3.62	2239	1380

Complex_Time point	Index	Residue	AA	Distance	Ligand Atom	Protein Atom
Tro3-cTnI100	1	38A	LYS	3.57	2021	290
Tro3-cTnI100	2	112A	TYR	3.68	2306	889
Tro3-cTnI100	3	141A	ARG	3.47	1919	1121
Tro3-cTnI100	4	191A	TRP	3.31	2239	1525
Tro3-cTnI0	1	158A	LEU	3.96	3259	1594
Tro3-cTnI0	2	164A	LYS	3.82	2917	1646
Tro3-cTnI0	3	167A	LEU	3.9	2949	1678
Tro3-cTnI0	4	208A	PHE	3.2	3009	2111
Tro3-cTnI0	5	209A	GLU	3.71	2949	2122
Tro4-cTnI20	1	37A	LYS	3.85	2005	282
Tro4-cTnI20	2	139A	PHE	3.68	1842	1101
Tro4-cTnI20	3	139A	PHE	3.74	1843	1102
Tro4-cTnI20	4	147A	VAL	3.6	2364	1177
Tro4-cTnI20	5	149A	ILE	3.83	1692	1197
Tro4-cTnI20	6	149A	ILE	3.71	2345	1194
Tro4-cTnI20	7	149A	ILE	3.66	2339	1195
Tro4-cTnI40	1	36A	LYS	3.98	2005	273
Tro4-cTnI40	2	139A	PHE	3.42	1844	1103
Tro4-cTnI40	3	139A	PHE	3.93	1843	1105
Tro4-cTnI40	4	147A	VAL	3.97	2364	1177
Tro4-cTnI40	5	149A	ILE	3.56	2339	1195
Tro4-cTnI60	1	37A	LYS	3.69	1999	281
Tro4-cTnI60	2	139A	PHE	3.96	1844	1103
Tro4-cTnI60	3	147A	VAL	3.84	2364	1177
Tro4-cTnI60	4	149A	ILE	3.87	2345	1194
Tro4-cTnI60	5	149A	ILE	3.54	2339	1195
Tro4-cTnI60	6	152A	ASP	3.87	2402	1213
Tro4-cTnI80	1	10A	ARG	3.92	1986	58
Tro4-cTnI80	2	139A	PHE	3.74	1842	1101
Tro4-cTnI80	3	139A	PHE	3.55	1843	1103
Tro4-cTnI80	4	149A	ILE	3.85	2339	1195
Tro4-cTnI80	5	207A	LYS	3.89	2402	1652
Tro4-cTnI100	1	10A	ARG	3.83	1986	58
Tro4-cTnI100	2	139A	PHE	3.73	1843	1104
Tro4-cTnI100	3	140A	LYS	3.77	1844	1114
Tro4-cTnI100	4	153A	ALA	3.95	2383	1221
Tro4-cTnI0	1	149A	ILE	3.66	3163	1518
Tro4-cTnI0	2	209A	GLU	3.84	3383	2121
Tro4-cTnI0	3	209A	GLU	3.69	3257	2122
Tro5-cTnI0	1	46A	LYS	3.34	2722	452
Tro5-cTnI0	2	54A	LEU	3.73	2632	536
Tro5-cTnI0	3	57A	ALA	3.52	2630	563
Tro5-cTnI0	4	153A	ALA	3.77	2349	1550

Complex_Time point	Index	Residue	AA	Distance	Ligand Atom	Protein Atom
Tro5-cTnI0	5	209A	GLU	3.8	2413	2121
Tro5-cTnI20	1	40A	LYS	3.93	2061	305
Tro5-cTnI20	2	149A	ILE	3.66	1997	1195
Tro5-cTnI20	3	154A	MET	3.7	1997	1226
Tro5-cTnI20	4	164A	LYS	3.96	2381	1299
Tro5-cTnI40	1	40A	LYS	3.9	2061	306
Tro5-cTnI40	2	126A	ALA	3.89	1978	996
Tro5-cTnI40	3	140A	LYS	3.91	1965	1114
Tro5-cTnI40	4	146A	ARG	3.65	1978	1165
Tro5-cTnI40	5	149A	ILE	3.84	1984	1197
Tro5-cTnI40	6	161A	ALA	3.85	1779	1276
Tro5-cTnI60	1	139A	PHE	3.7	2143	1106
Tro5-cTnI60	2	140A	LYS	3.75	2163	1114
Tro5-cTnI60	3	146A	ARG	3.83	1965	1166
Tro5-cTnI60	4	146A	ARG	3.96	1978	1165
Tro5-cTnI60	5	149A	ILE	3.47	1984	1197
Tro5-cTnI60	6	149A	ILE	3.87	1997	1194
Tro5-cTnI60	7	161A	ALA	3.79	1778	1276
Tro5-cTnI60	8	204A	ARG	3.78	2343	1622
Tro5-cTnI80	1	146A	ARG	3.52	1978	1165
Tro5-cTnI80	2	149A	ILE	3.89	1984	1196
Tro5-cTnI80	3	156A	GLN	3.83	1821	1242
Tro5-cTnI80	4	161A	ALA	3.67	2401	1276
Tro5-cTnI80	5	204A	ARG	3.73	2343	1622
Tro5-cTnI100	1	47A	LEU	3.74	1719	361
Tro5-cTnI100	2	139A	PHE	3.75	2143	1106
Tro5-cTnI100	3	140A	LYS	3.91	1965	1114
Tro5-cTnI100	4	146A	ARG	3.82	1978	1165
Tro5-cTnI100	5	149A	ILE	3.52	1997	1195
Tro6-cTnI0	1	149A	ILE	3.68	2601	1521
Tro6-cTnI20	1	36A	LYS	3.95	1922	273
Tro6-cTnI20	2	161A	ALA	3.77	2038	1276
Tro6-cTnI20	3	205A	LYS	3.81	2123	1634
Tro6-cTnI80	1	40A	LYS	3.88	1698	304
Tro6-cTnI80	2	205A	LYS	3.86	2123	1635
Tro6-cTnI60	1	194A	ASN	3.42	2145	1554
Tro6-cTnI100	1	19A	ILE	3.68	2335	127
Tro6-cTnI100	2	19A	ILE	3.75	2341	128
Tro6-cTnI100	3	40A	LYS	3.66	1692	304
Tro6-cTnI100	4	194A	ASN	3.79	2145	1554

Table A.2. Hydrogen Bond

Complex_Time point	Index	Residue	AA	Distance HA	Distance DA	Donor Angle	Donor Atom	Acceptor Atom
Tro1-cTnI20	1	19A	ILE	2.54	3.31	135.14	2108 [Nar]	132 [O2]
Tro1-cTnI20	2	21A	ARG	2.12	2.96	141.47	144 [Nam]	2107 [O2]
Tro1-cTnI20	3	21A	ARG	3.32	3.66	102.05	151 [Ng+]	2060 [O3]
Tro1-cTnI20	4	21A	ARG	2	2.97	169.55	152 [Ng+]	2101 [O3]
Tro1-cTnI20	5	22A	ARG	3.56	3.92	104.84	2096 [O3]	165 [O2]
Tro1-cTnI20	6	23A	SER	2.98	3.75	137.1	169 [O3]	1909 [O2]
Tro1-cTnI20	7	23A	SER	2.67	3.34	125.45	1910 [Nar]	169 [O3]
Tro1-cTnI20	8	24A	SER	2.27	3.23	164.81	172 [Nam]	1930 [O2]
Tro1-cTnI20	9	29A	TYR	1.91	2.75	141.63	2066 [Nar]	225 [O2]
Tro1-cTnI20	10	36A	LYS	2.16	3.03	147.38	269 [Nam]	2329 [O2]
Tro1-cTnI20	11	36A	LYS	2.16	2.98	136.19	275 [N3]	2301 [O3]
Tro1-cTnI20	12	39A	SER	2.49	3.34	146.8	299 [O3]	2306 [O2]
Tro1-cTnI20	13	40A	LYS	1.89	2.84	163.53	2327 [Nar]	310 [O2]
Tro1-cTnI20	14	42A	SER	1.94	2.83	151.03	322 [O3]	2404 [Npl]
Tro1-cTnI20	15	42A	SER	1.9	2.83	155.78	2404 [Npl]	322 [O3]
Tro1-cTnI20	16	43A	ALA	3.65	3.96	101.37	325 [Nam]	2357 [O2]
Tro1-cTnI20	17	44A	SER	2.24	3.05	140.29	333 [O3]	2409 [O2]
Tro1-cTnI20	18	44A	SER	1.81	2.73	153.63	2407 [Nar]	333 [O3]
Tro1-cTnI20	19	44A	SER	1.99	2.9	152.03	330 [Nam]	2357 [O2]
Tro1-cTnI20	20	45A	ARG	3.62	3.97	103.77	343 [Ng+]	2409 [O2]
Tro1-cTnI20	21	48A	GLN	1.98	2.94	163.82	370 [Nam]	2034 [O3]
Tro1-cTnI20	22	55A	GLN	2.53	3.49	163.77	427 [Nam]	1922 [O3]
Tro1-cTnI20	23	81A	GLN	1.86	2.79	156.75	1694 [Nar]	640 [O2]
Tro1-cTnI20	24	83A	LEU	2.54	3.09	114.94	648 [Nam]	1693 [O2]
Tro1-cTnI20	25	165A	GLU	3.32	3.68	103.93	1970 [Npl]	1309 [O3]
Tro1-cTnI20	26	166A	SER	2.2	2.83	121.54	1316 [O3]	1937 [O3]
Tro1-cTnI40	1	20A	ARG	2.31	3	126.99	141 [Ng+]	2135 [O2]
Tro1-cTnI40	2	21A	ARG	1.98	2.93	161.73	144 [Nam]	2107 [O2]
Tro1-cTnI40	3	21A	ARG	1.94	2.89	162.85	152 [Ng+]	2101 [O3]
Tro1-cTnI40	4	34A	HIS	2.18	3.13	161.92	2272 [Npl]	257 [N2]
Tro1-cTnI40	5	36A	LYS	1.97	2.94	156.18	275 [N3]	2301 [O3]
Tro1-cTnI40	6	37A	LYS	2.82	3.53	129.8	2307 [Nar]	286 [O2]
Tro1-cTnI40	7	39A	SER	2.31	3.22	155.23	299 [O3]	2329 [O2]
Tro1-cTnI40	8	40A	LYS	3.08	3.91	139.96	308 [N3]	2327 [Nar]
Tro1-cTnI40	9	45A	ARG	2.22	2.91	125.96	344 [Ng+]	2068 [O2]
Tro1-cTnI40	10	48A	GLN	2.12	3.06	159.51	370 [Nam]	2032 [O3]
Tro1-cTnI40	11	55A	GLN	3	3.37	103.59	427 [Nam]	1922 [O3]
Tro1-cTnI40	12	59A	GLN	2.53	3.05	112.75	458 [Nam]	1930 [O2]
Tro1-cTnI40	13	69A	ARG	2.62	3.02	104.24	548 [Ng+]	1797 [Npl]
Tro1-cTnI40	14	94A	GLN	2.9	3.81	154.01	733 [Nam]	1744 [O2]
Tro1-cTnI40	15	98A	ARG	2.21	2.91	127.41	766 [Ng+]	1748 [O3]
Tro1-cTnI40	16	98A	ARG	3.36	3.79	108.63	765 [Ng+]	1754 [O2]

Complex_Time point	Index	Residue	AA	Distance HA	Distance DA	Donor Angle	Donor Atom	Acceptor Atom
Tro1-cTnI40	17	101A	HIS	2.21	3.06	145.79	1685 [O3]	792 [N2]
Tro1-cTnI40	18	105A	ASP	2.09	3.02	172.09	823 [O3]	1738 [Npl]
Tro1-cTnI40	19	105A	ASP	2.18	3.02	142.99	1738 [Npl]	823 [O3]
Tro1-cTnI40	20	141A	ARG	3.06	3.7	123.53	1126 [Ng+]	1795 [Nar]
Tro1-cTnI40	21	162A	ARG	3.26	3.6	101.79	1287 [Ng+]	1980 [O3]
Tro1-cTnI40	22	166A	SER	3.08	4.01	161.39	1316 [O3]	1939 [O3]
Tro1-cTnI40	23	190A	ASP	2.83	3.17	101.25	1893 [Npl]	1517 [O2]
Tro1-cTnI40	24	191A	TRP	2.85	3.71	147.42	1872 [Nar]	1531 [O2]
Tro1-cTnI40	25	205A	LYS	2.36	3.18	136.75	1637 [N3]	1970 [Npl]
Tro1-cTnI40	26	205A	LYS	2.4	3.18	135.88	1970 [Npl]	1637 [N3]
Tro1-cTnI40	27	206A	LYS	2.68	3.24	116.73	1640 [Nam]	1967 [O2]
Tro1-cTnI40	28	207A	LYS	2.56	3.38	140.77	1649 [Nam]	1967 [O2]
Tro1-cTnI60	1	19A	ILE	3.53	3.89	103.92	2108 [Nam]	132 [O2]
Tro1-cTnI60	2	21A	ARG	2.42	3.22	137.67	152 [Ng+]	2107 [O2]
Tro1-cTnI60	3	21A	ARG	2.69	3.63	160.88	144 [Nam]	2108 [Nam]
Tro1-cTnI60	4	31A	THR	2.88	3.4	113.82	231 [Nam]	2065 [O2]
Tro1-cTnI60	5	31A	THR	1.92	2.9	169.48	2066 [Nar]	237 [O2]
Tro1-cTnI60	6	34A	HIS	2.19	3.15	165.82	2327 [Nar]	263 [O2]
Tro1-cTnI60	7	36A	LYS	2.94	3.34	105.58	269 [Nam]	2329 [O2]
Tro1-cTnI60	8	36A	LYS	1.92	2.9	159.29	275 [N3]	2301 [O3]
Tro1-cTnI60	9	37A	LYS	2.74	3.66	155.9	278 [Nam]	2256 [O2]
Tro1-cTnI60	10	42A	SER	1.94	2.87	161.43	2337 [O3]	322 [O3]
Tro1-cTnI60	11	42A	SER	3	3.9	155.42	322 [O3]	2336 [O2]
Tro1-cTnI60	12	48A	GLN	2.79	3.46	126.31	370 [Nam]	1949 [Nar]
Tro1-cTnI60	13	55A	GLN	3.1	3.75	124.59	427 [Nam]	1922 [O3]
Tro1-cTnI60	14	69A	ARG	1.91	2.82	151.6	548 [Ng+]	1731 [Nar]
Tro1-cTnI60	15	69A	ARG	3.39	3.99	121.34	549 [Ng+]	1731 [Nar]
Tro1-cTnI60	16	98A	ARG	2.24	3.07	141.03	765 [Ng+]	1735 [Nar]
Tro1-cTnI60	17	101A	HIS	3.02	3.39	104.48	1685 [O3]	789 [N2]
Tro1-cTnI60	18	162A	ARG	2.82	3.48	125.16	1286 [Ng+]	1970 [Npl]
Tro1-cTnI60	19	165A	GLU	2.24	2.95	127.71	1970 [Npl]	1310 [O3]
Tro1-cTnI60	20	166A	SER	2.81	3.47	125.75	1316 [O3]	1954 [O3]
Tro1-cTnI60	21	186A	ARG	3.28	3.97	128.83	1487 [Ng+]	1833 [Nam]
Tro1-cTnI60	22	192A	ARG	3.08	3.86	136.42	1539 [Ng+]	1865 [O3]
Tro1-cTnI60	23	207A	LYS	2.69	3.09	104.66	1649 [Nam]	1967 [O2]
Tro1-cTnI80	1	19A	ILE	2.41	3.14	130.65	2110 [N2]	132 [O2]
Tro1-cTnI80	2	20A	ARG	2.77	3.65	148.09	141 [Ng+]	2135 [O2]
Tro1-cTnI80	3	20A	ARG	3.27	3.85	119.93	140 [Ng+]	2108 [Nar]
Tro1-cTnI80	4	21A	ARG	2.75	3.68	159.12	144 [Nam]	2107 [O2]
Tro1-cTnI80	5	21A	ARG	1.89	2.84	161.8	152 [Ng+]	2101 [O3]

Complex_Time point	Index	Residue	AA	Distance HA	Distance DA	Donor Angle	Donor Atom	Acceptor Atom
Tro1-cTnI80	6	25A	ASN	3.23	3.61	105.37	183 [Nam]	1853 [O2]
Tro1-cTnI80	7	25A	ASN	2.01	2.88	146	178 [Nam]	1853 [O2]
Tro1-cTnI80	8	31A	THR	2.66	3.13	109.3	231 [Nam]	2065 [O2]
Tro1-cTnI80	9	31A	THR	2	2.8	137.5	2066 [Nar]	237 [O2]
Tro1-cTnI80	10	34A	HIS	2.28	3.12	143.35	2327 [Nar]	263 [O2]
Tro1-cTnI80	11	36A	LYS	3.27	3.62	103.01	269 [Nam]	2329 [O2]
Tro1-cTnI80	12	37A	LYS	1.55	2.54	175.88	278 [Nam]	2256 [O3]
Tro1-cTnI80	13	42A	SER	2.3	2.85	114.98	322 [O3]	2334 [O3]
Tro1-cTnI80	14	44A	SER	3.43	4.06	125.21	333 [O3]	2409 [O2]
Tro1-cTnI80	15	45A	ARG	2.97	3.64	126.35	344 [Ng+]	2068 [O2]
Tro1-cTnI80	16	48A	GLN	2.22	3.16	159.08	370 [Nam]	1949 [Nar]
Tro1-cTnI80	17	48A	GLN	2.06	3.04	173.91	2386 [Nar]	369 [O2]
Tro1-cTnI80	18	105A	ASP	3.45	4.01	118.37	1738 [Npl]	824 [O3]
Tro1-cTnI80	19	162A	ARG	2.91	3.76	145.14	1287 [Ng+]	1975 [O3]
Tro1-cTnI80	20	166A	SER	3.22	3.95	133.45	1316 [O3]	1938 [O2]
Tro1-cTnI80	21	177A	LYS	3.46	3.91	109.04	1408 [N3]	1826 [O3]
Tro1-cTnI80	22	181A	THR	3.06	4.01	165.54	1440 [O3]	1835 [Npl]
Tro1-cTnI80	23	186A	ARG	2.37	3.26	152.81	1821 [O3]	1489 [O2]
Tro1-cTnI80	24	190A	ASP	2.71	3.5	137.89	1510 [Nam]	1893 [Npl]
Tro1-cTnI80	25	193A	LYS	1.82	2.83	170.59	1549 [N3]	1871 [O2]
Tro1-cTnI100	1	19A	ILE	2.85	3.56	129.8	2108 [Nar]	132 [O2]
Tro1-cTnI100	2	19A	ILE	1.94	2.89	160.14	2110 [N2]	132 [O2]
Tro1-cTnI100	3	21A	ARG	2.74	3.54	139.48	144 [Nam]	2107 [O2]
Tro1-cTnI100	4	21A	ARG	2.05	2.89	142.63	152 [Ng+]	2101 [O3]
Tro1-cTnI100	5	24A	SER	2.77	3.46	127.87	1912 [Npl]	177 [O2]
Tro1-cTnI100	6	25A	ASN	2.59	3.45	146.23	183 [Nam]	1910 [Nar]
Tro1-cTnI100	7	31A	THR	1.97	2.84	146.27	231 [Nam]	2065 [O2]
Tro1-cTnI100	8	31A	THR	2.08	2.88	137.28	2066 [Nar]	237 [O2]
Tro1-cTnI100	9	36A	LYS	1.92	2.8	142.63	275 [N3]	2301 [O3]
Tro1-cTnI100	10	37A	LYS	1.76	2.71	161.12	278 [Nam]	2256 [O3]
Tro1-cTnI100	11	42A	SER	2.74	3.36	121.87	322 [O3]	2326 [O2]
Tro1-cTnI100	12	43A	ALA	2.89	3.26	103.26	325 [Nam]	2337 [O2]
Tro1-cTnI100	13	44A	SER	2.62	3.13	113.15	333 [O3]	2326 [O2]
Tro1-cTnI100	14	44A	SER	1.83	2.8	167.06	330 [Nam]	2337 [O2]
Tro1-cTnI100	15	48A	GLN	3.29	3.86	118.73	370 [Nam]	1949 [Nar]
Tro1-cTnI100	16	55A	GLN	3.19	3.97	136.72	427 [Nam]	1917 [O2]
Tro1-cTnI100	17	66A	GLU	3.4	4.07	128.09	1704 [O3]	520 [O2]
Tro1-cTnI100	18	69A	ARG	3.5	3.88	105.41	548 [Ng+]	1731 [Nar]
Tro1-cTnI100	19	94A	GLN	1.78	2.76	172.45	733 [Nam]	1784 [O2]
Tro1-cTnI100	20	98A	ARG	3.52	3.99	111.48	766 [Ng+]	1783 [O3]
Tro1-cTnI100	21	100A	LEU	3.23	4.02	139.43	1685 [O3]	785 [O2]
Tro1-cTnI100	22	105A	ASP	2.71	3.27	116.01	1738 [Npl]	824 [O3]
Tro1-cTnI100	23	162A	ARG	3.16	3.64	111.11	1286 [Ng+]	1970 [Npl]

Complex_Time point	Index	Residue	AA	Distance HA	Distance DA	Donor Angle	Donor Atom	Acceptor Atom
Tro1-cTnI100	24	162A	ARG	2.69	3.14	108.18	1287 [Ng+]	1980 [O3]
Tro1-cTnI100	25	165A	GLU	2.64	3.2	116.76	1970 [Npl]	1309 [O3]
Tro1-cTnI100	26	166A	SER	2.71	3.49	138.57	1316 [O3]	1954 [O3]
Tro1-cTnI100	27	186A	ARG	2.61	3.37	136.11	1821 [O3]	1489 [O2]
Tro1-cTnI100	28	191A	TRP	2.1	2.99	148.85	1874 [Npl]	1531 [O2]
Tro1-cTnI100	29	192A	ARG	3.52	3.9	105.31	1540 [Ng+]	1874 [Npl]
Tro1-cTnI100	30	192A	ARG	3.44	3.75	100.79	1893 [Npl]	1537 [Ng+]
Tro1-cTnI100	31	192A	ARG	3.62	3.94	101.47	1539 [Ng+]	1871 [O2]
Tro1-cTnI100	32	202A	GLU	1.91	2.83	154.38	1607 [Nam]	1986 [O2]
Tro1-cTnI100	33	203A	GLY	3.03	3.79	134.95	1616 [Nam]	1975 [O3]
Tro1-cTnI100	34	204A	ARG	3.55	4.05	113.29	1620 [Nam]	1976 [O2]
Tro1-cTnI100	35	206A	LYS	1.68	2.65	167.95	1640 [Nam]	1967 [O2]
Tro1-cTnI0	1	29A	TYR	3.5	4.04	116.94	275 [Nam]	2734 [O2]
Tro1-cTnI0	2	29A	TYR	1.81	2.8	163.11	2735 [Nam]	288 [O2]
Tro1-cTnI0	3	31A	THR	2.61	3.54	159.97	299 [O3]	2992 [N2]
Tro1-cTnI0	4	31A	THR	2.94	3.29	101.12	2988 [Npl]	299 [O3]
Tro1-cTnI0	5	32A	GLU	2.62	3.27	121.95	3050 [Npl]	313 [O2]
Tro1-cTnI0	6	34A	HIS	2.31	3.25	162	321 [Nam]	3050 [Npl]
Tro1-cTnI0	7	36A	LYS	2.86	3.77	156.03	339 [Nam]	3145 [O2]
Tro1-cTnI0	8	40A	LYS	3.44	3.75	101.01	386 [Nam]	3142 [Nam]
Tro1-cTnI0	9	40A	LYS	1.87	2.83	156.92	3142 [Nam]	398 [O2]
Tro1-cTnI0	10	42A	SER	2.91	3.81	153.67	408 [Nam]	3206 [Nam]
Tro1-cTnI0	11	51A	THR	2.99	3.85	150.74	507 [O3]	2550 [O2]
Tro1-cTnI0	12	55A	GLN	2.02	2.95	157.33	546 [Nam]	2518 [O2]
Tro1-cTnI0	13	62A	GLU	3.32	3.73	106.07	2369 [Npl]	616 [O3]
Tro1-cTnI0	14	69A	ARG	1.88	2.79	143.06	699 [Ng+]	2306 [O2]
Tro1-cTnI0	15	69A	ARG	2.47	3.21	127.55	702 [Ng+]	2306 [O2]
Tro1-cTnI0	16	98A	ARG	2.79	3.2	105.62	967 [Ng+]	2154 [O2]
Tro1-cTnI0	17	98A	ARG	2.68	3.11	104.15	973 [Ng+]	2154 [O2]
Tro1-cTnI0	18	166A	SER	2	2.93	160.59	1668 [O3]	2535 [O3]
Tro1-cTnI0	19	174A	LYS	2.02	3	155.44	1750 [N3+]	2400 [O2]
Tro1-cTnI0	20	190A	ASP	1.98	2.89	147.64	2431 [Npl]	1919 [O2]
Tro2-cTnI0	1	141A	ARG	2.29	3.08	136.49	1414 [Nam]	2225 [O2]
Tro2-cTnI0	2	143A	THR	2.65	3.53	150.7	1442 [O3]	2258 [O2]
Tro2-cTnI0	3	150A	SER	2.31	3.27	162.51	1524 [Nam]	2508 [O3]
Tro2-cTnI0	4	150A	SER	1.92	2.87	166.47	1528 [O3]	2508 [O3]
Tro2-cTnI0	5	150A	SER	2.3	2.87	116.84	2508 [O3]	1528 [O3]
Tro2-cTnI0	6	157A	ALA	2.77	3.53	133.02	2368 [Npl]	1588 [O2]

Complex_Time point	Index	Residue	AA	Distance HA	Distance DA	Donor Angle	Donor Atom	Acceptor Atom
Tro2-cTnI0	7	175A	GLN	3.24	3.87	123.31	1763 [Nam]	3133 [O3]
Tro2-cTnI0	8	178A	LYS	2.87	3.9	174.5	1796 [N3+]	3150 [O2]
Tro2-cTnI0	9	207A	LYS	2.43	3.25	140.15	2093 [Nam]	3164 [O2]
Tro2-cTnI0	10	210A	SER	2.89	3.83	163.11	2132 [O3]	3132 [O3]
Tro2-cTnI0	11	210A	SER	3.04	3.83	139.65	3132 [O3]	2132 [O3]
Tro2-cTnI20	1	37A	LYS	2.47	2.93	106.15	284 [N3]	1847 [O3]
Tro2-cTnI20	2	37A	LYS	2.8	3.53	131.34	278 [Nam]	1813 [O2]
Tro2-cTnI20	3	38A	LYS	3.29	3.67	104.89	287 [Nam]	1822 [O3]
Tro2-cTnI20	4	39A	SER	2.19	3.16	168.65	296 [Nam]	1822 [O3]
Tro2-cTnI20	5	44A	SER	3.35	3.65	100.18	333 [O3]	2059 [O2]
Tro2-cTnI20	6	50A	LYS	3	3.64	121.58	387 [N3]	1830 [Nar]
Tro2-cTnI20	7	136A	ARG	3.14	3.84	129.25	1082 [Ng+]	1721 [O2]
Tro2-cTnI20	8	141A	ARG	2.25	3.05	137.76	1127 [Ng+]	1685 [O3]
Tro2-cTnI20	9	142A	PRO	2.01	2.95	163.84	1685 [O3]	1136 [O2]
Tro2-cTnI20	10	143A	THR	2.17	2.72	114.79	1140 [O3]	1740 [O2]
Tro2-cTnI20	11	146A	ARG	2.99	3.86	146.81	1171 [Ng+]	1767 [O3]
Tro2-cTnI20	12	147A	VAL	2.98	3.73	134.36	1797 [N2]	1180 [O2]
Tro2-cTnI20	13	148A	ARG	2.66	3.35	127.11	1188 [Ng+]	1788 [O3]
Tro2-cTnI20	14	148A	ARG	2.33	3.08	132.78	1189 [Ng+]	1788 [O3]
Tro2-cTnI20	15	149A	ILE	3.15	4.1	161.49	1192 [Nam]	1794 [O2]
Tro2-cTnI20	16	202A	GLU	3.21	3.79	121.44	1612 [O3]	2414 [O2]
Tro2-cTnI20	17	204A	ARG	2.9	3.51	121.45	1620 [Nam]	2339 [O2]
Tro2-cTnI20	18	207A	LYS	2.23	2.88	120.45	1655 [N3]	2324 [O3]
Tro2-cTnI20	19	207A	LYS	2.38	3.23	143.72	1649 [Nam]	2329 [O2]
Tro2-cTnI40	1	36A	LYS	3.47	3.99	114.94	1816 [Npl]	275 [N3]
Tro2-cTnI40	2	36A	LYS	2.48	3.37	145.29	275 [N3]	1813 [O2]
Tro2-cTnI40	3	37A	LYS	2.33	3.3	167.06	278 [Nam]	1813 [O2]
Tro2-cTnI40	4	37A	LYS	3.66	4.09	107.71	284 [N3]	1853 [O2]
Tro2-cTnI40	5	38A	LYS	2.75	3.38	122.23	287 [Nam]	1822 [O3]
Tro2-cTnI40	6	39A	SER	2.94	3.92	174.8	296 [Nam]	1822 [O3]
Tro2-cTnI40	7	43A	ALA	2.69	3.63	161.01	2054 [Npl]	329 [O2]
Tro2-cTnI40	8	44A	SER	3.43	3.87	109.5	333 [O3]	1994 [O2]
Tro2-cTnI40	9	50A	LYS	2.12	2.89	130.65	387 [N3]	1830 [Nar]
Tro2-cTnI40	10	141A	ARG	1.85	2.8	160.02	1119 [Nam]	1721 [O2]
Tro2-cTnI40	11	142A	PRO	1.94	2.7	133.17	1685 [O3]	1136 [O2]
Tro2-cTnI40	12	143A	THR	3.43	3.94	115.08	1140 [O3]	1870 [Npl]
Tro2-cTnI40	13	143A	THR	3.51	3.94	109.09	1870 [Npl]	1140 [O3]
Tro2-cTnI40	14	146A	ARG	2.87	3.67	139.21	1170 [Ng+]	1866 [O3]
Tro2-cTnI40	15	146A	ARG	2.34	3.26	155.83	1171 [Ng+]	1866 [O3]
Tro2-cTnI40	16	147A	VAL	2.32	3.17	144.21	1797 [Npl]	1180 [O2]

Complex_Time point	Index	Residue	AA	Distance HA	Distance DA	Donor Angle	Donor Atom	Acceptor Atom
Tro2-cTnI40	17	147A	VAL	3.27	4.08	140.91	1174 [Nam]	1908 [O3]
Tro2-cTnI40	18	149A	ILE	1.94	2.91	170.73	1192 [Nam]	1795 [Nar]
Tro2-cTnI40	19	161A	ALA	1.75	2.67	154.05	1274 [Nam]	2040 [O2]
Tro2-cTnI40	20	205A	LYS	3.35	4.05	127.5	1637 [N3]	2477 [O2]
Tro2-cTnI60	1	37A	LYS	1.77	2.74	157.85	284 [N3]	1807 [O3]
Tro2-cTnI60	2	37A	LYS	3.19	3.74	117.14	278 [Nam]	1813 [O2]
Tro2-cTnI60	3	38A	LYS	1.85	2.79	158.37	287 [Nam]	1822 [O3]
Tro2-cTnI60	4	43A	ALA	3.42	3.84	108.3	2054 [Npl]	329 [O2]
Tro2-cTnI60	5	136A	ARG	3.68	4.07	106.48	1083 [Ng+]	1721 [O2]
Tro2-cTnI60	6	146A	ARG	2	2.92	153.68	1170 [Ng+]	1908 [O3]
Tro2-cTnI60	7	147A	VAL	2.89	3.85	165.95	1174 [Nam]	1915 [Nar]
Tro2-cTnI60	8	149A	ILE	2.81	3.74	157.18	1192 [Nam]	1795 [Nar]
Tro2-cTnI60	9	157A	ALA	1.78	2.72	159.09	1936 [Npl]	1253 [O2]
Tro2-cTnI60	10	161A	ALA	2.06	3.03	170.44	1274 [Nam]	2040 [O2]
Tro2-cTnI60	11	162A	ARG	2.89	3.58	127.54	1287 [Ng+]	2064 [O3]
Tro2-cTnI60	12	162A	ARG	3.14	3.88	133.34	1279 [Nam]	2040 [O2]
Tro2-cTnI80	1	35A	ALA	2.31	3.29	175.75	264 [Nam]	1803 [O3]
Tro2-cTnI80	2	36A	LYS	3.15	4	142.11	275 [N3]	1778 [Npl]
Tro2-cTnI80	3	36A	LYS	3.3	4	130.1	1778 [Npl]	275 [N3]
Tro2-cTnI80	4	37A	LYS	2.22	3.18	162.61	278 [Nam]	1813 [O2]
Tro2-cTnI80	5	43A	ALA	2.87	3.64	135.58	2052 [Nar]	329 [O2]
Tro2-cTnI80	6	46A	LYS	3.07	3.7	120.74	353 [N3]	1970 [O2]
Tro2-cTnI80	7	50A	LYS	1.87	2.79	147.78	387 [N3]	1973 [O2]
Tro2-cTnI80	8	140A	LYS	2.33	2.99	121.52	1116 [N3]	1713 [O2]
Tro2-cTnI80	9	142A	PRO	2.06	2.82	132.76	1870 [Npl]	1136 [O2]
Tro2-cTnI80	10	146A	ARG	2.01	2.94	155.01	1163 [Nam]	1783 [O2]
Tro2-cTnI80	11	146A	ARG	2.11	2.72	118.36	1170 [Ng+]	1908 [O3]
Tro2-cTnI80	12	147A	VAL	2.09	3.04	162.83	1797 [Npl]	1180 [O2]
Tro2-cTnI80	13	147A	VAL	2.33	3.31	171.65	1174 [Nam]	1915 [Nar]
Tro2-cTnI80	14	148A	ARG	1.92	2.91	177.36	1188 [Ng+]	1794 [O2]
Tro2-cTnI80	15	162A	ARG	1.86	2.62	131.02	1286 [Ng+]	2064 [O3]
Tro2-cTnI80	16	162A	ARG	2.41	3.06	122.3	1287 [Ng+]	2064 [O3]
Tro2-cTnI80	17	203A	GLY	2.93	3.56	123.37	1616 [Nam]	2431 [Nar]
Tro2-cTnI80	18	204A	ARG	2.74	3.4	124.51	1627 [Ng+]	2441 [O3]
Tro2-cTnI80	19	207A	LYS	2.69	3.34	121.57	1655 [N3]	2309 [O2]
Tro2-cTnI100	1	36A	LYS	3.1	3.78	125.01	275 [N3]	1778 [Npl]
Tro2-cTnI100	2	36A	LYS	3.03	3.78	133.84	1778 [Npl]	275 [N3]
Tro2-cTnI100	3	37A	LYS	2.12	3.05	157.08	278 [Nam]	1813 [O2]

Complex_Time point	Index	Residue	AA	Distance HA	Distance DA	Donor Angle	Donor Atom	Acceptor Atom
Tro2-cTnI100	4	48A	GLN	3.3	3.96	126.26	370 [Nam]	2060 [O2]
Tro2-cTnI100	5	50A	LYS	2.75	3.5	132.47	1837 [Npl]	387 [N3]
Tro2-cTnI100	6	50A	LYS	1.72	2.71	161.3	387 [N3]	1933 [O2]
Tro2-cTnI100	7	141A	ARG	1.69	2.64	160.75	1119 [Nam]	1713 [O2]
Tro2-cTnI100	8	144A	LEU	2.03	3.01	169.64	1144 [Nam]	1893 [O2]
Tro2-cTnI100	9	146A	ARG	3.54	3.89	103.7	1170 [Ng+]	1901 [O3]
Tro2-cTnI100	10	146A	ARG	1.92	2.9	176.57	1163 [Nam]	1783 [O2]
Tro2-cTnI100	11	147A	VAL	3.5	3.88	105.08	1174 [Nam]	1797 [Npl]
Tro2-cTnI100	12	147A	VAL	1.75	2.73	173.49	1797 [Npl]	1180 [O2]
Tro2-cTnI100	13	148A	ARG	2.87	3.79	155.73	1188 [Ng+]	1795 [Nar]
Tro2-cTnI100	14	161A	ALA	2.48	3.37	150.16	1274 [Nam]	2040 [O3]
Tro2-cTnI100	15	162A	ARG	2.06	2.85	135.7	1286 [Ng+]	2064 [O3]
Tro2-cTnI100	16	162A	ARG	2.07	2.86	135.79	1287 [Ng+]	2064 [O3]
Tro2-cTnI100	17	202A	GLU	2.08	2.95	146.29	2475 [Nar]	1613 [O2]
Tro2-cTnI100	18	203A	GLY	2.97	3.66	128.34	1616 [Nam]	2477 [O2]
Tro2-cTnI100	19	204A	ARG	3.28	3.81	115.71	1627 [Ng+]	2448 [O3]
Tro2-cTnI100	20	204A	ARG	2.12	2.85	130.35	1628 [Ng+]	2448 [O3]
Tro2-cTnI100	21	205A	LYS	3.4	3.8	106.5	1631 [Nam]	2186 [Nar]
Tro2-cTnI100	22	207A	LYS	2.41	3.28	142.73	1655 [N3]	2375 [Nar]
Tro2-cTnI100	23	207A	LYS	2.47	3.42	161.62	1649 [Nam]	2332 [O2]
Tro2-cTnI100	24	208A	PHE	2.5	3.27	135.44	1658 [Nam]	2309 [O2]
Tro2-cTnI100	25	208A	PHE	2.79	3.5	130.41	2310 [Nar]	1668 [O2]
Tro3-cTnI20	1	37A	LYS	1.85	2.72	141.41	284 [N3]	2420 [O2]
Tro3-cTnI20	2	38A	LYS	2.09	3	148.09	293 [N3]	2042 [Nar]
Tro3-cTnI20	3	38A	LYS	2.41	3.2	136.43	2421 [Nar]	293 [N3]
Tro3-cTnI20	4	41A	ILE	2.06	3.03	168.1	311 [Nam]	2026 [O3]
Tro3-cTnI20	5	41A	ILE	2.61	3.42	141.16	2026 [O3]	318 [O2]
Tro3-cTnI20	6	46A	LYS	1.99	2.86	140.95	353 [N3]	2031 [O3]
Tro3-cTnI20	7	46A	LYS	3.15	3.83	127.76	2035 [Npl]	353 [N3]
Tro3-cTnI20	8	50A	LYS	2.02	2.84	135.6	387 [N3]	2401 [O2]
Tro3-cTnI20	9	108A	ASP	2.68	3.38	127.8	2305 [Npl]	850 [O2]
Tro3-cTnI20	10	117A	LYS	2.27	2.73	105.4	928 [N3]	1841 [O2]
Tro3-cTnI20	11	145A	ARG	1.98	2.8	138.6	1152 [Nam]	1870 [O3]
Tro3-cTnI20	12	146A	ARG	2.89	3.47	118.53	1171 [Ng+]	1862 [Nar]
Tro3-cTnI20	13	146A	ARG	2.92	3.78	145.78	1170 [Ng+]	1875 [O3]
Tro3-cTnI20	14	146A	ARG	2.1	3.07	169.13	1163 [Nam]	1870 [O3]

Complex_Time point	Index	Residue	AA	Distance HA	Distance DA	Donor Angle	Donor Atom	Acceptor Atom
Tro3-cTnI20	15	156A	GLN	1.88	2.8	153.28	1246 [Nam]	2189 [O3]
Tro3-cTnI20	16	174A	LYS	2.95	3.56	119.28	1383 [N3]	2235 [O3]
Tro3-cTnI20	17	208A	PHE	2.63	3.33	128.68	2284 [N2]	1668 [O2]
Tro3-cTnI40	1	20A	ARG	3.25	3.86	121.43	141 [Ng+]	2260 [Nar]
Tro3-cTnI40	2	37A	LYS	2.09	3	152.44	278 [Nam]	2490 [O3]
Tro3-cTnI40	3	37A	LYS	2.12	3.03	152.87	284 [Npl]	2433 [O3]
Tro3-cTnI40	4	38A	LYS	3.41	3.98	117.16	293 [N3]	2042 [Nar]
Tro3-cTnI40	5	38A	LYS	2.33	3.31	168.95	287 [Nam]	2040 [O2]
Tro3-cTnI40	6	41A	ILE	2.47	3.34	147.88	311 [Nam]	2026 [O3]
Tro3-cTnI40	7	42A	SER	1.71	2.66	159.81	319 [Nam]	2026 [O3]
Tro3-cTnI40	8	46A	LYS	2.29	2.67	100.69	353 [N3]	2031 [O3]
Tro3-cTnI40	9	69A	ARG	2.76	3.26	112.09	548 [Ng+]	2260 [Nar]
Tro3-cTnI40	10	69A	ARG	3.33	3.94	121.8	549 [Ng+]	2221 [Nar]
Tro3-cTnI40	11	108A	ASP	1.9	2.83	157.17	2305 [Npl]	848 [O2]
Tro3-cTnI40	12	131A	LYS	1.8	2.77	158.78	1037 [N3]	1781 [Nar]
Tro3-cTnI40	13	141A	ARG	3.18	3.63	109.7	1119 [Nam]	2471 [O3]
Tro3-cTnI40	14	141A	ARG	2.72	3.15	107.16	2471 [O3]	1129 [O2]
Tro3-cTnI40	15	143A	THR	1.92	2.77	145.33	1140 [O3]	2450 [O2]
Tro3-cTnI40	16	145A	ARG	1.95	2.77	139.26	1152 [Nam]	1870 [O2]
Tro3-cTnI40	17	146A	ARG	2.14	3.01	146.22	1163 [Nam]	1870 [O2]
Tro3-cTnI40	18	146A	ARG	3.29	4.04	134.5	1171 [Ng+]	1883 [Nar]
Tro3-cTnI40	19	146A	ARG	2.8	3.74	158.71	1170 [Ng+]	1875 [O3]
Tro3-cTnI40	20	153A	ALA	2.51	3.2	127.38	2404 [Npl]	1223 [O2]
Tro3-cTnI40	21	174A	LYS	2.56	3.26	128.12	2244 [Npl]	1385 [O2]
Tro3-cTnI40	22	174A	LYS	3.35	3.9	115.76	1383 [N3]	2235 [O3]
Tro3-cTnI60	1	21A	ARG	2.33	3.03	127.26	151 [Ng+]	2254 [O3]
Tro3-cTnI60	2	21A	ARG	2.09	2.85	132.56	152 [Ng+]	2254 [O3]
Tro3-cTnI60	3	38A	LYS	2.34	3.32	169.39	287 [Nam]	2040 [O2]
Tro3-cTnI60	4	38A	LYS	2.52	3.36	139.19	293 [N3]	2040 [O2]
Tro3-cTnI60	5	40A	LYS	3.46	3.82	103.43	308 [N3]	2011 [O3]
Tro3-cTnI60	6	41A	ILE	2.04	3.02	171.69	311 [Nam]	2027 [O3]
Tro3-cTnI60	7	42A	SER	2.1	3	150.89	319 [Nam]	2027 [O3]
Tro3-cTnI60	8	46A	LYS	2.41	3.3	145.14	353 [N3]	2036 [Nar]
Tro3-cTnI60	9	50A	LYS	2.87	3.25	103.91	2116 [Npl]	387 [N3]
Tro3-cTnI60	10	69A	ARG	3.13	3.77	123.87	548 [Ng+]	2220 [O2]
Tro3-cTnI60	11	69A	ARG	1.77	2.7	156.95	549 [Ng+]	2220 [O2]
Tro3-cTnI60	12	112A	TYR	1.7	2.61	162.83	887 [O3]	2311 [O3]
Tro3-cTnI60	13	112A	TYR	2.08	2.61	112.44	2311 [O3]	887 [O3]
Tro3-cTnI60	14	131A	LYS	2.19	3.13	151.83	1037 [N3]	1781 [Nar]
Tro3-cTnI60	15	141A	ARG	2.11	3.02	153.95	1119 [Nam]	2472 [O3]
Tro3-cTnI60	16	143A	THR	2.41	2.91	111.78	2450 [O3]	1140 [O3]

Complex_Time point	Index	Residue	AA	Distance HA	Distance DA	Donor Angle	Donor Atom	Acceptor Atom
Tro3-cTnI60	17	143A	THR	3.4	3.87	111.91	1140 [O3]	2449 [O2]
Tro3-cTnI60	18	145A	ARG	2.35	3.12	134.39	1152 [Nam]	1870 [O3]
Tro3-cTnI60	19	146A	ARG	2.02	2.96	157.84	1163 [Nam]	1870 [O3]
Tro3-cTnI60	20	174A	LYS	2.46	3.26	138.18	2244 [Npl]	1385 [O2]
Tro3-cTnI60	21	210A	SER	2.44	3.39	163.84	1681 [O3]	2305 [Npl]
Tro3-cTnI60	22	210A	SER	2.63	3.39	133.88	2305 [Npl]	1681 [O3]
Tro3-cTnI80	1	21A	ARG	3.6	4.04	109.51	151 [Ng+]	2258 [Nar]
Tro3-cTnI80	2	21A	ARG	2.45	3.07	120.61	152 [Ng+]	2258 [Nar]
Tro3-cTnI80	3	38A	LYS	2.18	3.07	145.51	293 [N3]	2042 [Nar]
Tro3-cTnI80	4	41A	ILE	2.05	2.88	140.34	311 [Nam]	2026 [O2]
Tro3-cTnI80	5	42A	SER	1.91	2.84	155.72	319 [Nam]	2026 [O2]
Tro3-cTnI80	6	46A	LYS	2.74	3.71	158.78	353 [N3]	2035 [Npl]
Tro3-cTnI80	7	50A	LYS	1.82	2.82	164.67	387 [N3]	2414 [O3]
Tro3-cTnI80	8	108A	ASP	2.68	3.62	159.62	2305 [Npl]	847 [O2]
Tro3-cTnI80	9	112A	TYR	1.71	2.65	174.88	887 [O3]	2311 [O3]
Tro3-cTnI80	10	127A	ASP	1.74	2.65	152.57	1760 [Npl]	1003 [O2]
Tro3-cTnI80	11	131A	LYS	1.9	2.9	166.22	1037 [N3]	1781 [Npl]
Tro3-cTnI80	12	131A	LYS	1.96	2.9	158.46	1781 [Npl]	1037 [N3]
Tro3-cTnI80	13	145A	ARG	1.68	2.62	159.38	1152 [Nam]	1870 [O3]
Tro3-cTnI80	14	145A	ARG	3.34	3.66	101.21	1160 [Ng+]	1760 [Npl]
Tro3-cTnI80	15	146A	ARG	2.27	3.23	163.06	1163 [Nam]	1870 [O3]
Tro3-cTnI80	16	146A	ARG	3.34	3.99	125.58	1171 [Ng+]	1883 [Nar]
Tro3-cTnI80	17	146A	ARG	2.97	3.79	142.23	1170 [Ng+]	1875 [O3]
Tro3-cTnI80	18	174A	LYS	3.22	3.8	119.52	2244 [Npl]	1385 [O2]
Tro3-cTnI80	19	174A	LYS	2.32	3.13	135.25	1383 [N3]	2235 [O3]
Tro3-cTnI100	1	21A	ARG	2.25	3.07	140.24	152 [Ng+]	2254 [O3]
Tro3-cTnI100	2	22A	ARG	1.93	2.88	159.75	162 [Ng+]	2260 [Nar]
Tro3-cTnI100	3	40A	LYS	3.27	3.6	102.17	302 [Nam]	2016 [O2]
Tro3-cTnI100	4	41A	ILE	2.05	3.02	166.9	311 [Nam]	2026 [O3]
Tro3-cTnI100	5	42A	SER	1.75	2.66	152.36	319 [Nam]	2026 [O3]
Tro3-cTnI100	6	46A	LYS	2.79	3.73	153.01	353 [N3]	2421 [Nar]
Tro3-cTnI100	7	50A	LYS	1.96	2.93	156.74	387 [N3]	2420 [O2]
Tro3-cTnI100	8	72A	LYS	2.57	3.03	107.24	571 [N3]	2298 [Nar]
Tro3-cTnI100	9	108A	ASP	1.74	2.71	168.95	2305 [Npl]	848 [O3]
Tro3-cTnI100	10	127A	ASP	2.62	3.41	138.1	1699 [Npl]	1004 [O2]
Tro3-cTnI100	11	127A	ASP	1.75	2.69	159.07	1760 [Npl]	1003 [O3]
Tro3-cTnI100	12	145A	ARG	1.75	2.69	157.5	1152 [Nam]	1870 [O3]
Tro3-cTnI100	13	145A	ARG	2.93	3.45	114.43	1160 [Ng+]	1760 [Npl]
Tro3-cTnI100	14	146A	ARG	2.16	3.01	143.56	1163 [Nam]	1870 [O3]
Tro3-cTnI100	15	146A	ARG	2.72	3.32	119.31	1171 [Ng+]	1883 [Nar]

Complex_Time point	Index	Residue	AA	Distance HA	Distance DA	Donor Angle	Donor Atom	Acceptor Atom
Tro3-cTnI100	16	146A	ARG	2.03	2.88	142.73	1170 [Ng+]	1875 [O3]
Tro3-cTnI100	17	150A	SER	3.11	3.56	109.35	1203 [O3]	2320 [O2]
Tro3-cTnI0	1	40A	LYS	2.11	2.79	120.79	393 [N3+]	2691 [O2]
Tro3-cTnI0	2	141A	ARG	3.51	3.98	109.14	1426 [Ng+]	2494 [O3]
Tro3-cTnI0	3	145A	ARG	2	2.89	149.98	1456 [Nam]	2424 [O3]
Tro3-cTnI0	4	146A	ARG	2.59	3.52	149.08	1482 [Ng+]	2405 [N2]
Tro3-cTnI0	5	146A	ARG	2.55	3.43	149.44	1473 [Nam]	2424 [O3]
Tro3-cTnI0	6	157A	ALA	1.96	2.88	150.17	3256 [Npl]	1588 [O2]
Tro3-cTnI0	7	161A	ALA	2.01	2.96	161.87	1612 [Nam]	2860 [O3]
Tro3-cTnI0	8	174A	LYS	2.71	3.14	105.79	3006 [Npl]	1755 [O2]
Tro3-cTnI0	9	175A	GLN	2.5	3.23	130.06	1763 [Nam]	3004 [Nam]
Tro3-cTnI0	10	209A	GLU	2.69	3.41	133.85	2125 [O3]	3065 [O2]
Tro3-cTnI0	11	210A	SER	2.28	2.88	118.64	2128 [Nam]	3051 [O3]
Tro4-cTnI20	1	35A	ALA	3.51	4.07	118.58	264 [Nam]	2004 [Npl]
Tro4-cTnI20	2	35A	ALA	2.06	2.97	153.09	2004 [Npl]	268 [O2]
Tro4-cTnI20	3	37A	LYS	1.8	2.79	178.74	278 [Nam]	2001 [O2]
Tro4-cTnI20	4	38A	LYS	1.86	2.85	161.17	293 [N3]	1941 [Nar]
Tro4-cTnI20	5	38A	LYS	3.44	4	118.36	1943 [Npl]	293 [N3]
Tro4-cTnI20	6	40A	LYS	3.09	3.75	125.36	2237 [Npl]	308 [N3]
Tro4-cTnI20	7	40A	LYS	1.98	2.91	150.22	308 [N3]	2238 [Nar]
Tro4-cTnI20	8	141A	ARG	2.45	3.37	155.83	1119 [Nam]	1848 [O2]
Tro4-cTnI20	9	146A	ARG	2.4	3.04	122.17	1171 [Ng+]	2354 [O3]
Tro4-cTnI20	10	150A	SER	3.2	3.76	117.89	2368 [O3]	1203 [O3]
Tro4-cTnI20	11	150A	SER	1.77	2.73	164.36	1200 [Nam]	2369 [O2]
Tro4-cTnI20	12	204A	ARG	2.9	3.62	130.8	1628 [Ng+]	2436 [O2]
Tro4-cTnI40	1	37A	LYS	2.6	3.48	149.05	278 [Nam]	2002 [Nar]
Tro4-cTnI40	2	37A	LYS	2.94	3.62	126.79	2162 [Npl]	284 [N3]
Tro4-cTnI40	3	37A	LYS	2.34	3.03	123.63	284 [N3]	2157 [Nar]
Tro4-cTnI40	4	38A	LYS	3.33	3.92	118.49	293 [N3]	1934 [O3]
Tro4-cTnI40	5	143A	THR	2.78	3.69	155.98	1140 [O3]	1864 [Npl]
Tro4-cTnI40	6	143A	THR	2.9	3.69	137.94	1864 [Npl]	1140 [O3]
Tro4-cTnI40	7	146A	ARG	3.61	3.96	103.68	1171 [Ng+]	2360 [O2]
Tro4-cTnI40	8	150A	SER	2.1	2.99	148.99	1200 [Nam]	2368 [O3]
Tro4-cTnI40	9	204A	ARG	3.21	4	138.75	1628 [Ng+]	2436 [O2]
Tro4-cTnI40	10	204A	ARG	3.53	3.87	102.92	1627 [Ng+]	2477 [Nar]
Tro4-cTnI60	1	6A	SER	2.92	3.71	139.31	35 [O3]	1985 [Npl]
Tro4-cTnI60	2	6A	SER	3.28	3.71	108.14	1985 [Npl]	35 [O3]
Tro4-cTnI60	3	10A	ARG	3.3	3.89	120.38	63 [Ng+]	1978 [Nar]
Tro4-cTnI60	4	37A	LYS	2.38	3.31	157.04	278 [Nam]	2002 [Nar]

Complex_Time point	Index	Residue	AA	Distance HA	Distance DA	Donor Angle	Donor Atom	Acceptor Atom
Tro4-cTnI60	5	37A	LYS	1.92	2.78	140.45	284 [N3]	2014 [O3]
Tro4-cTnI60	6	38A	LYS	2.2	3.2	165.42	293 [N3]	2177 [O2]
Tro4-cTnI60	7	140A	LYS	3.15	3.69	114.75	1116 [N3]	1833 [O3]
Tro4-cTnI60	8	146A	ARG	2.42	2.97	114.65	1171 [Ng+]	2354 [O3]
Tro4-cTnI60	9	150A	SER	2.89	3.8	154.17	1200 [Nam]	2366 [O3]
Tro4-cTnI60	10	204A	ARG	3.21	3.71	112.98	1628 [Ng+]	2471 [O3]
Tro4-cTnI60	11	207A	LYS	3.6	4.03	107.58	1655 [N3]	2477 [Nar]
Tro4-cTnI80	1	5A	SER	1.88	2.84	166.72	26 [Nam]	1910 [O2]
Tro4-cTnI80	2	10A	ARG	3.58	4.03	109.92	63 [Ng+]	2004 [Npl]
Tro4-cTnI80	3	10A	ARG	2.9	3.41	113.47	64 [Ng+]	2004 [Npl]
Tro4-cTnI80	4	11A	GLU	2.98	3.56	121.39	73 [O3]	1985 [Npl]
Tro4-cTnI80	5	11A	GLU	3.05	3.56	113.8	1985 [Npl]	73 [O3]
Tro4-cTnI80	6	36A	LYS	3.52	4.08	118.74	2196 [Npl]	277 [O2]
Tro4-cTnI80	7	37A	LYS	1.84	2.72	147.43	278 [Nam]	2001 [O2]
Tro4-cTnI80	8	37A	LYS	1.95	2.94	162.92	284 [N3]	1940 [O2]
Tro4-cTnI80	9	37A	LYS	2.18	2.98	136.65	2021 [Nam]	284 [N3]
Tro4-cTnI80	10	40A	LYS	3.07	3.75	127.32	2237 [Npl]	308 [N3]
Tro4-cTnI80	11	40A	LYS	2.1	3.01	147.85	308 [N3]	2238 [Nar]
Tro4-cTnI80	12	141A	ARG	2.57	3.25	126.41	1126 [Ng+]	1857 [Nar]
Tro4-cTnI80	13	141A	ARG	1.86	2.72	144.07	1127 [Ng+]	1857 [Nar]
Tro4-cTnI80	14	149A	ILE	2.25	2.81	115.41	1192 [Nam]	2350 [O2]
Tro4-cTnI80	15	150A	SER	3.42	3.76	103.07	2368 [O3]	1203 [O3]
Tro4-cTnI80	16	150A	SER	2.66	3.56	153.62	1200 [Nam]	2366 [O3]
Tro4-cTnI80	17	205A	LYS	1.83	2.75	154.09	2482 [Npl]	1639 [O2]
Tro4-cTnI80	18	207A	LYS	1.93	2.87	158.28	1649 [Nam]	2400 [O2]
Tro4-cTnI100	1	1A	MET	1.58	2.58	163.26	1 [N3]	1948 [O2]
Tro4-cTnI100	2	5A	SER	2.51	3.43	155.56	26 [Nam]	1910 [O2]
Tro4-cTnI100	3	10A	ARG	1.85	2.78	155.62	64 [Ng+]	1995 [O3]
Tro4-cTnI100	4	37A	LYS	2.26	3.21	162.53	278 [Nam]	2001 [O2]
Tro4-cTnI100	5	37A	LYS	2.08	2.98	144.81	284 [N3]	1940 [O2]
Tro4-cTnI100	6	40A	LYS	2.95	3.65	129.12	2237 [Npl]	308 [N3]
Tro4-cTnI100	7	40A	LYS	3.04	3.78	130.68	308 [N3]	2233 [O3]
Tro4-cTnI100	8	148A	ARG	3.65	4.06	107.19	1188 [Ng+]	2331 [O2]
Tro4-cTnI100	9	149A	ILE	2.56	3.44	149.08	1192 [Nam]	2350 [O2]
Tro4-cTnI100	10	150A	SER	2.03	2.92	148.78	1200 [Nam]	2369 [O2]
Tro4-cTnI100	11	204A	ARG	2.08	2.89	138.79	1628 [Ng+]	2436 [O2]
Tro4-cTnI100	12	207A	LYS	2.45	3.09	119.63	1655 [N3]	2400 [O2]
Tro4-cTnI0	1	40A	LYS	2.15	3.17	164.42	393 [N3+]	2992 [O3]
Tro4-cTnI0	2	150A	SER	2.68	3.32	124.14	3202 [O3]	1528 [O3]

Complex_Time point	Index	Residue	AA	Distance HA	Distance DA	Donor Angle	Donor Atom	Acceptor Atom
Tro4-cTnI0	3	150A	SER	2.1	2.97	148.85	1528 [O3]	3203 [O2]
Tro4-cTnI0	4	150A	SER	2.22	3.18	164.39	1524 [Nam]	3203 [O2]
Tro4-cTnI0	5	168A	ASP	2.13	3.05	165.14	1686 [O3]	3376 [Npl]
Tro4-cTnI0	6	168A	ASP	2.76	3.61	142.35	3376 [Npl]	1689 [O2]
Tro4-cTnI0	7	170A	ARG	3.24	4.1	141.17	1711 [Ng+]	3248 [Nam]
Tro4-cTnI0	8	207A	LYS	2.82	3.52	124.77	2100 [N3+]	3371 [N2]
Tro4-cTnI0	9	208A	PHE	3.24	3.98	133.23	2106 [Nam]	3371 [N2]
Tro4-cTnI0	10	210A	SER	2.29	2.86	116.29	2128 [Nam]	3265 [O2]
Tro4-cTnI0	11	210A	SER	3.37	3.91	116.97	2132 [O3]	3388 [O3]
Tro5-cTnI0	1	40A	LYS	2.47	3.44	153.54	393 [N3+]	2717 [Nam]
Tro5-cTnI0	2	50A	LYS	2.31	3.34	172.51	497 [N3+]	2624 [O2]
Tro5-cTnI0	3	53A	LEU	2.72	3.27	113.94	2627 [Npl]	529 [O2]
Tro5-cTnI0	4	143A	THR	3.16	4.01	147.53	1442 [O3]	2594 [O2]
Tro5-cTnI0	5	146A	ARG	2.97	3.99	163.93	1482 [Ng+]	2905 [O2]
Tro5-cTnI0	6	147A	VAL	2.45	3.44	177.2	1490 [Nam]	2549 [O3]
Tro5-cTnI0	7	147A	VAL	3.26	4.09	144.48	2549 [O3]	1497 [O2]
Tro5-cTnI0	8	148A	ARG	3.13	4.09	159.38	2374 [Npl]	1514 [O2]
Tro5-cTnI0	9	150A	SER	2.57	3.52	167.46	1528 [O3]	2358 [O3]
Tro5-cTnI0	10	161A	ALA	2.79	3.65	143.6	3215 [Nam]	1617 [O2]
Tro5-cTnI0	11	175A	GLN	2.95	3.88	159.89	1763 [Nam]	3153 [N2]
Tro5-cTnI0	12	207A	LYS	2.86	3.81	153.4	2100 [N3+]	3145 [O3]
Tro5-cTnI0	13	207A	LYS	2.31	3.26	164.27	2093 [Nam]	3153 [N2]
Tro5-cTnI20	1	38A	LYS	1.9	2.83	157.05	287 [Nam]	2085 [O2]
Tro5-cTnI20	2	40A	LYS	3.2	3.72	113.3	308 [N3]	2118 [O2]
Tro5-cTnI20	3	42A	SER	3.24	3.88	124.81	322 [O3]	1736 [Nar]
Tro5-cTnI20	4	46A	LYS	2.01	2.86	138.54	353 [N3]	1755 [O2]
Tro5-cTnI20	5	46A	LYS	2.47	3.23	133.47	1756 [Nar]	353 [N3]
Tro5-cTnI20	6	140A	LYS	1.82	2.76	150.91	1116 [N3]	2172 [O3]
Tro5-cTnI20	7	141A	ARG	2.23	3.18	162.81	2181 [Npl]	1129 [O2]
Tro5-cTnI20	8	146A	ARG	1.78	2.74	162.57	1171 [Ng+]	2178 [O2]
Tro5-cTnI20	9	146A	ARG	2.53	3.45	154.33	1983 [N2]	1173 [O2]
Tro5-cTnI20	10	147A	VAL	2.39	3.35	162.31	1174 [Nam]	1950 [O3]
Tro5-cTnI20	11	150A	SER	3.77	4.06	100.66	1203 [O3]	1834 [Nar]
Tro5-cTnI20	12	150A	SER	2	2.92	154.93	2002 [Npl]	1205 [O2]
Tro5-cTnI20	13	161A	ALA	2.56	3.52	166.53	1274 [Nam]	1763 [O3]
Tro5-cTnI20	14	204A	ARG	2.91	3.48	118.4	1628 [Ng+]	2335 [Nar]
Tro5-cTnI20	15	207A	LYS	1.74	2.75	166.77	1655 [N3]	2331 [O3]

Complex_Time point	Index	Residue	AA	Distance HA	Distance DA	Donor Angle	Donor Atom	Acceptor Atom
Tro5-cTnI40	1	40A	LYS	2.65	3.28	119.65	308 [N3]	2118 [O2]
Tro5-cTnI40	2	42A	SER	2.72	3.69	174	322 [O3]	1734 [Nar]
Tro5-cTnI40	3	46A	LYS	3.48	3.84	103.18	353 [N3]	2060 [Npl]
Tro5-cTnI40	4	46A	LYS	3.24	3.84	120.7	2060 [Npl]	353 [N3]
Tro5-cTnI40	5	140A	LYS	3.24	3.91	126.87	1110 [Nam]	2148 [O3]
Tro5-cTnI40	6	146A	ARG	1.91	2.78	146.09	1983 [Npl]	1173 [O2]
Tro5-cTnI40	7	146A	ARG	3.08	3.59	113.6	1171 [Ng+]	2172 [O3]
Tro5-cTnI40	8	147A	VAL	1.79	2.75	163.85	1174 [Nam]	1950 [O3]
Tro5-cTnI40	9	148A	ARG	2.27	3.13	145.02	1841 [Npl]	1191 [O2]
Tro5-cTnI40	10	150A	SER	2.9	3.3	105.66	1200 [Nam]	1841 [Npl]
Tro5-cTnI40	11	165A	GLU	1.72	2.7	172.73	2397 [Nam]	1310 [O2]
Tro5-cTnI40	12	204A	ARG	2.47	3.24	134.5	1620 [Nam]	2337 [Nar]
Tro5-cTnI40	13	204A	ARG	2.56	3.4	143.2	1628 [Ng+]	2352 [O3]
Tro5-cTnI40	14	206A	LYS	3.6	4.06	109.32	1646 [N3]	2339 [Nar]
Tro5-cTnI60	1	37A	LYS	3.14	4.06	150.89	284 [N3]	2118 [O2]
Tro5-cTnI60	2	38A	LYS	2.1	3.05	161.83	287 [Nam]	2086 [O2]
Tro5-cTnI60	3	40A	LYS	2.37	2.85	107.52	308 [N3]	2118 [O2]
Tro5-cTnI60	4	46A	LYS	3.16	3.62	108.93	353 [N3]	2060 [Npl]
Tro5-cTnI60	5	46A	LYS	3.25	3.62	104.26	2060 [Npl]	353 [N3]
Tro5-cTnI60	6	60A	GLU	2.08	3.05	166.4	2002 [Npl]	466 [O2]
Tro5-cTnI60	7	130A	GLN	3.03	3.8	136.47	1028 [Nam]	1971 [O3]
Tro5-cTnI60	8	140A	LYS	1.95	2.85	150.44	1110 [Nam]	2147 [O3]
Tro5-cTnI60	9	146A	ARG	1.98	2.86	148.17	1983 [Npl]	1173 [O2]
Tro5-cTnI60	10	147A	VAL	1.89	2.85	165.49	1174 [Nam]	1950 [O3]
Tro5-cTnI60	11	147A	VAL	2.9	3.85	166.85	1950 [O3]	1180 [O2]
Tro5-cTnI60	12	148A	ARG	3.11	3.84	131.55	1189 [Ng+]	1980 [O2]
Tro5-cTnI60	13	148A	ARG	2.12	3.02	151.91	1841 [Npl]	1191 [O2]
Tro5-cTnI60	14	150A	SER	2.5	3.26	133.27	1200 [Nam]	1841 [Npl]
Tro5-cTnI60	15	152A	ASP	2.89	3.75	147.82	1825 [O3]	1218 [O2]
Tro5-cTnI60	16	162A	ARG	3.39	3.75	103.77	1287 [Ng+]	1776 [O2]
Tro5-cTnI60	17	175A	GLN	3.4	3.86	111.03	1392 [Nam]	2337 [Nar]
Tro5-cTnI60	18	179A	GLU	3.04	3.36	101.87	1426 [O3]	2342 [Npl]
Tro5-cTnI60	19	204A	ARG	2.56	3.32	134.04	1628 [Ng+]	2352 [O3]
Tro5-cTnI60	20	206A	LYS	3.22	3.71	111.5	1646 [N3]	2342 [Npl]
Tro5-cTnI60	21	206A	LYS	2.87	3.71	144.12	2342 [Npl]	1646 [N3]
Tro5-cTnI60	22	207A	LYS	3.07	4.03	163.54	1649 [Nam]	2337 [Nar]
Tro5-cTnI80	1	17A	ALA	1.92	2.8	147.78	1718 [Npl]	117 [O2]

Complex_Time point	Index	Residue	AA	Distance HA	Distance DA	Donor Angle	Donor Atom	Acceptor Atom
Tro5-cTnI80	2	17A	ALA	3.16	3.8	124.79	113 [Nam]	1713 [Nar]
Tro5-cTnI80	3	19A	ILE	3.21	3.88	126.55	125 [Nam]	1718 [Npl]
Tro5-cTnI80	4	20A	ARG	2.49	3.34	144.18	140 [Ng+]	1707 [O3]
Tro5-cTnI80	5	20A	ARG	2.85	3.62	134.89	141 [Ng+]	1707 [O3]
Tro5-cTnI80	6	39A	SER	2.5	3.4	152.49	296 [Nam]	2075 [O2]
Tro5-cTnI80	7	40A	LYS	2.31	2.81	108.43	308 [N3]	2057 [O2]
Tro5-cTnI80	8	46A	LYS	3.32	3.87	117.63	2060 [Npl]	353 [N3]
Tro5-cTnI80	9	141A	ARG	1.93	2.89	165.03	1127 [Ng+]	2172 [O3]
Tro5-cTnI80	10	146A	ARG	2.01	2.84	139.58	1983 [Npl]	1173 [O2]
Tro5-cTnI80	11	146A	ARG	3.54	3.95	107.67	1171 [Ng+]	2172 [O3]
Tro5-cTnI80	12	147A	VAL	2.16	3.14	173.85	1174 [Nam]	1950 [O3]
Tro5-cTnI80	13	147A	VAL	2.97	3.66	128.92	1950 [O3]	1180 [O2]
Tro5-cTnI80	14	150A	SER	2.28	3.13	145.36	1203 [O3]	1841 [Npl]
Tro5-cTnI80	15	150A	SER	2.51	3.13	120.09	1841 [Npl]	1203 [O3]
Tro5-cTnI80	16	153A	ALA	2.84	3.34	112.02	1219 [Nam]	1841 [Npl]
Tro5-cTnI80	17	156A	GLN	2.71	3.05	100.87	1246 [Nam]	1880 [O2]
Tro5-cTnI80	18	158A	LEU	2.07	2.91	142.82	1739 [Npl]	1261 [O2]
Tro5-cTnI80	19	161A	ALA	2.98	3.52	115.58	1274 [Nam]	1805 [O3]
Tro5-cTnI80	20	163A	ALA	3.12	3.75	123.02	1290 [Nam]	1739 [Npl]
Tro5-cTnI80	21	204A	ARG	2.61	3.34	130.96	1627 [Ng+]	2299 [O2]
Tro5-cTnI80	22	204A	ARG	1.9	2.81	152.46	1628 [Ng+]	2299 [O2]
Tro5-cTnI80	23	207A	LYS	3.03	3.95	154.79	1649 [Nam]	2337 [Nar]
Tro5-cTnI100	1	13A	ARG	2.12	2.98	145.78	90 [Ng+]	1736 [Npl]
Tro5-cTnI100	2	17A	ALA	2.23	3.2	168.92	1718 [Npl]	117 [O2]
Tro5-cTnI100	3	17A	ALA	2.78	3.42	123.58	113 [Nam]	1713 [Nar]
Tro5-cTnI100	4	20A	ARG	3.36	3.74	104.98	140 [Ng+]	1713 [Nar]
Tro5-cTnI100	5	20A	ARG	2.83	3.41	118.47	141 [Ng+]	1707 [O3]
Tro5-cTnI100	6	21A	ARG	3.45	3.9	110.1	151 [Ng+]	1697 [N2]
Tro5-cTnI100	7	48A	GLN	2.27	2.86	117.37	1697 [N2]	369 [O2]
Tro5-cTnI100	8	140A	LYS	3.68	4.06	104.31	1116 [N3]	1961 [O2]
Tro5-cTnI100	9	143A	THR	1.88	2.82	163.41	1140 [O3]	2178 [O2]
Tro5-cTnI100	10	146A	ARG	3.39	3.8	107.41	1171 [Ng+]	2172 [O3]
Tro5-cTnI100	11	146A	ARG	1.93	2.9	170.35	1983 [Npl]	1173 [O2]
Tro5-cTnI100	12	147A	VAL	1.94	2.92	171.76	1174 [Nam]	1950 [O3]
Tro5-cTnI100	13	148A	ARG	2.87	3.6	131.91	1189 [Ng+]	1980 [O2]
Tro5-cTnI100	14	149A	ILE	2.59	3.06	109.39	2002 [Npl]	1199 [O2]
Tro5-cTnI100	15	161A	ALA	2.61	3.58	170.08	1274 [Nam]	1785 [O3]
Tro5-cTnI100	16	162A	ARG	3.3	3.83	116.71	1783 [O3]	1284 [Ng+]

Complex_Time point	Index	Residue	AA	Distance HA	Distance DA	Donor Angle	Donor Atom	Acceptor Atom
Tro5-cTnI100	17	162A	ARG	2.26	3.06	138.24	1279 [Nam]	1784 [O2]
Tro5-cTnI100	18	179A	GLU	3.06	3.9	144.12	2342 [Npl]	1425 [O2]
Tro5-cTnI100	19	204A	ARG	2.35	3.22	147.17	1628 [Ng+]	2352 [O3]
Tro5-cTnI100	20	207A	LYS	2.23	3.13	151.09	1649 [Nam]	2339 [Nar]
Tro6-cTnI0	1	46A	LYS	2.11	2.9	130.8	454 [N3+]	2496 [O2]
Tro6-cTnI0	2	142A	PRO	1.75	2.73	161.73	2435 [Npl]	1437 [O2]
Tro6-cTnI0	3	143A	THR	3.75	4.07	101.7	2526 [Npl]	1442 [O3]
Tro6-cTnI0	4	147A	VAL	3.09	4.05	164.76	1490 [Nam]	2608 [O2]
Tro6-cTnI0	5	147A	VAL	3.17	3.79	123.17	2577 [O3]	1497 [O2]
Tro6-cTnI0	6	149A	ILE	2.98	3.9	156.78	1515 [Nam]	2577 [O3]
Tro6-cTnI0	7	161A	ALA	2.2	2.81	118.95	1612 [Nam]	2166 [O3]
Tro6-cTnI0	8	172A	HIS	2.21	2.89	125.69	1730 [Nar]	2814 [O2]
Tro6-cTnI0	9	175A	GLN	2.27	2.83	115.44	1763 [Nam]	2779 [N2]
Tro6-cTnI0	10	179A	GLU	3.62	4.1	111.6	2845 [Npl]	1808 [O2]
Tro6-cTnI0	11	204A	ARG	2.18	3.07	149.34	2050 [Nam]	2795 [O3]
Tro6-cTnI0	12	204A	ARG	2.9	3.56	125.81	2056 [Ng+]	2764 [O3]
Tro6-cTnI0	13	208A	PHE	2.12	3	148.54	2106 [Nam]	2716 [O2]
Tro6-cTnI0	14	209A	GLU	2.65	3.52	149.35	2118 [Nam]	2716 [O2]
Tro6-cTnI0	15	209A	GLU	3.09	3.82	135.85	2125 [O3]	2716 [O2]
Tro6-cTnI0	16	209A	GLU	2.36	2.85	108.74	2717 [Nam]	2125 [O3]
Tro6-cTnI0	17	210A	SER	2.11	2.99	149.87	2132 [O3]	2734 [O3]
Tro6-cTnI20	1	37A	LYS	3.68	4.07	106.08	278 [Nam]	1918 [O2]
Tro6-cTnI20	2	37A	LYS	1.83	2.72	142.78	284 [N3]	1912 [O3]
Tro6-cTnI20	3	38A	LYS	2.63	3.54	154.1	287 [Nam]	1695 [Nar]
Tro6-cTnI20	4	141A	ARG	2.62	3.34	129.7	1126 [Ng+]	1811 [O3]
Tro6-cTnI20	5	141A	ARG	2.79	3.51	130.24	1127 [Ng+]	1811 [O3]
Tro6-cTnI20	6	141A	ARG	2.41	3.38	167.48	1119 [Nam]	1818 [Nar]
Tro6-cTnI20	7	141A	ARG	2.15	3.02	146.64	1820 [Npl]	1129 [O2]
Tro6-cTnI20	8	145A	ARG	3.29	3.7	107.38	1160 [Ng+]	1878 [Nar]
Tro6-cTnI20	9	148A	ARG	3.39	3.88	113.05	1189 [Ng+]	1874 [Nar]
Tro6-cTnI20	10	175A	GLN	3.61	4.07	111.26	1392 [Nam]	2098 [Nar]
Tro6-cTnI20	11	183A	LYS	2.02	2.86	141.95	2225 [N2]	1461 [O2]
Tro6-cTnI20	12	183A	LYS	1.93	2.73	133.05	1459 [N3]	2154 [O3]
Tro6-cTnI20	13	184A	GLU	1.69	2.66	166.67	2202 [Nar]	1470 [O2]
Tro6-cTnI20	14	185A	ASN	3.04	3.5	110	1476 [Nam]	2169 [O3]
Tro6-cTnI20	15	186A	ARG	3.33	3.77	108.71	1486 [Ng+]	2204 [O2]

Complex_Time point	Index	Residue	AA	Distance HA	Distance DA	Donor Angle	Donor Atom	Acceptor Atom
Tro6-cTnI20	16	186A	ARG	2.23	3.17	158.81	1479 [Nam]	2201 [O2]
Tro6-cTnI20	17	202A	GLU	2.63	3.34	129.34	1607 [Nam]	2121 [O2]
Tro6-cTnI20	18	205A	LYS	2.01	2.94	157.16	1631 [Nam]	2079 [O2]
Tro6-cTnI20	19	205A	LYS	3.31	3.83	113.67	1637 [N3]	2082 [Npl]
Tro6-cTnI20	20	205A	LYS	3.17	3.83	125.6	2082 [Npl]	1637 [N3]
Tro6-cTnI20	21	207A	LYS	2.33	3.2	146.07	1649 [Nam]	2100 [Nar]
Tro6-cTnI40	1	37A	LYS	2.23	3.2	166.54	278 [Nam]	1919 [Nar]
Tro6-cTnI40	2	40A	LYS	3.04	3.52	109.34	308 [N3]	2441 [O2]
Tro6-cTnI40	3	41A	ILE	2.4	3.16	133.16	311 [Nam]	1703 [O2]
Tro6-cTnI40	4	42A	SER	2.64	3.25	121.72	322 [O3]	2420 [O2]
Tro6-cTnI40	5	42A	SER	1.9	2.87	168.85	319 [Nam]	1694 [O2]
Tro6-cTnI40	6	45A	ARG	3.28	3.62	102.25	343 [Ng+]	2422 [Nar]
Tro6-cTnI40	7	45A	ARG	2.54	3.18	122.13	344 [Ng+]	2460 [Nar]
Tro6-cTnI40	8	46A	LYS	1.86	2.71	138.3	353 [N3]	2002 [O2]
Tro6-cTnI40	9	136A	ARG	2	2.74	129.43	1083 [Ng+]	1836 [O2]
Tro6-cTnI40	10	137A	GLY	2.3	3.2	151.14	1086 [Nam]	1836 [O2]
Tro6-cTnI40	11	138A	LYS	1.88	2.83	160.23	1090 [Nam]	1817 [O2]
Tro6-cTnI40	12	139A	PHE	2.71	3.38	126.11	1820 [N2]	1109 [O2]
Tro6-cTnI40	13	139A	PHE	2.28	3.18	151.47	1099 [Nam]	1818 [Nar]
Tro6-cTnI40	14	139A	PHE	3.15	3.76	121.63	1818 [Nar]	1109 [O2]
Tro6-cTnI40	15	146A	ARG	3.25	4.03	136.79	1163 [Nam]	1865 [O3]
Tro6-cTnI40	16	202A	GLU	2.03	2.84	137.98	1607 [Nam]	2121 [O2]
Tro6-cTnI80	1	37A	LYS	2.8	3.7	152.35	278 [Nam]	1918 [O2]
Tro6-cTnI80	2	39A	SER	1.92	2.9	172.01	2439 [Nar]	299 [O3]
Tro6-cTnI80	3	39A	SER	3.22	3.92	130.87	299 [O3]	2441 [O2]
Tro6-cTnI80	4	40A	LYS	1.96	2.86	152.14	302 [Nam]	2438 [O2]
Tro6-cTnI80	5	42A	SER	3.45	3.83	105.97	322 [O3]	1688 [O3]
Tro6-cTnI80	6	42A	SER	2.64	3	101.75	319 [Nam]	1694 [O2]
Tro6-cTnI80	7	43A	ALA	3.48	3.99	114	325 [Nam]	1718 [Nar]
Tro6-cTnI80	8	45A	ARG	2.78	3.77	176.39	1697 [Npl]	341 [Ng+]
Tro6-cTnI80	9	46A	LYS	1.91	2.71	133.39	353 [N3]	1716 [O2]
Tro6-cTnI80	10	46A	LYS	3.6	4.03	109.08	1738 [Npl]	353 [N3]
Tro6-cTnI80	11	137A	GLY	1.95	2.88	157.63	1086 [Nam]	1836 [O2]
Tro6-cTnI80	12	138A	LYS	2.47	3.41	158.78	1090 [Nam]	1817 [O2]
Tro6-cTnI80	13	139A	PHE	2.51	3.47	166.32	1099 [Nam]	1818 [Nar]
Tro6-cTnI80	14	139A	PHE	3.65	4.09	109.56	1818 [Nar]	1109 [O2]

Complex_Time point	Index	Residue	AA	Distance HA	Distance DA	Donor Angle	Donor Atom	Acceptor Atom
Tro6-cTnI80	15	146A	ARG	1.76	2.7	157.76	1163 [Nam]	1866 [O2]
Tro6-cTnI80	16	148A	ARG	2.95	3.52	117.38	1188 [Ng+]	1870 [O3]
Tro6-cTnI80	17	148A	ARG	3.52	4.02	113.63	1189 [Ng+]	1870 [O3]
Tro6-cTnI80	18	188A	VAL	2.33	2.92	117.58	2202 [Nar]	1505 [O2]
Tro6-cTnI80	19	192A	ARG	3.35	3.75	106.7	1539 [Ng+]	2181 [O2]
Tro6-cTnI80	20	194A	ASN	3.03	3.36	101.04	1557 [Nam]	2147 [O3]
Tro6-cTnI80	21	194A	ASN	1.76	2.72	161.45	1552 [Nam]	2150 [O3]
Tro6-cTnI80	22	199A	SER	2.27	3.08	139	2302 [Npl]	1594 [O2]
Tro6-cTnI80	23	203A	GLY	2.17	2.98	139.17	1616 [Nam]	2121 [O2]
Tro6-cTnI80	24	207A	LYS	3.1	4.01	154.33	1649 [Nam]	2100 [Nar]
Tro6-cTnI80	25	209A	GLU	2.34	3.32	171.28	2103 [Npl]	1674 [O3]
Tro6-cTnI60	1	21A	ARG	2.65	3.51	146.81	152 [Ng+]	2378 [O2]
Tro6-cTnI60	2	32A	GLU	3.38	3.86	114.02	243 [O3]	2429 [O2]
Tro6-cTnI60	3	37A	LYS	2.58	3.43	140	284 [N3]	1918 [O2]
Tro6-cTnI60	4	37A	LYS	2.09	3.02	155.49	278 [Nam]	1919 [Nar]
Tro6-cTnI60	5	42A	SER	3.54	4.04	114.24	322 [O3]	2420 [O2]
Tro6-cTnI60	6	42A	SER	1.77	2.71	159.12	319 [Nam]	1694 [O2]
Tro6-cTnI60	7	45A	ARG	2.68	3.26	118.45	344 [Ng+]	1697 [Npl]
Tro6-cTnI60	8	45A	ARG	2.67	3.63	164.12	1697 [Npl]	341 [Ng+]
Tro6-cTnI60	9	46A	LYS	1.94	2.81	141.61	353 [N3]	1716 [O2]
Tro6-cTnI60	10	46A	LYS	3.11	3.97	146.27	2024 [Npl]	353 [N3]
Tro6-cTnI60	11	46A	LYS	3.46	3.86	106.82	1738 [Npl]	353 [N3]
Tro6-cTnI60	12	137A	GLY	1.77	2.71	159.2	1086 [Nam]	1836 [O2]
Tro6-cTnI60	13	138A	LYS	2.04	3	164.52	1090 [Nam]	1817 [O2]
Tro6-cTnI60	14	139A	PHE	2.07	2.76	125.18	1820 [Npl]	1109 [O2]
Tro6-cTnI60	15	139A	PHE	2.8	3.78	171.75	1099 [Nam]	1818 [Nar]
Tro6-cTnI60	16	190A	ASP	2.13	3.01	149.09	1510 [Nam]	2204 [O2]
Tro6-cTnI60	17	194A	ASN	2.03	2.99	167.05	1552 [Nam]	2150 [O3]
Tro6-cTnI60	18	203A	GLY	2.26	2.93	124.13	1616 [Nam]	2121 [O2]
Tro6-cTnI60	19	204A	ARG	2.91	3.83	154.75	1628 [Ng+]	2048 [O2]
Tro6-cTnI100	1	21A	ARG	3.5	4.07	118.95	152 [Ng+]	2337 [O2]
Tro6-cTnI100	2	21A	ARG	2.29	2.97	125.98	151 [Ng+]	2380 [Nar]
Tro6-cTnI100	3	22A	ARG	2.24	3.15	154.29	155 [Nam]	2345 [O2]
Tro6-cTnI100	4	22A	ARG	2.24	3.04	137.28	163 [Ng+]	2391 [O3]
Tro6-cTnI100	5	36A	LYS	1.94	2.92	168.74	269 [Nam]	2441 [O2]
Tro6-cTnI100	6	37A	LYS	2.25	3.02	133.46	2481 [N2]	286 [O2]
Tro6-cTnI100	7	37A	LYS	2.31	3.26	161.98	278 [Nam]	1919 [Nar]

Complex_Time point	Index	Residue	AA	Distance HA	Distance DA	Donor Angle	Donor Atom	Acceptor Atom
Tro6-cTnI100	8	37A	LYS	1.75	2.7	152.22	284 [N3]	1912 [O3]
Tro6-cTnI100	9	39A	SER	2.06	2.95	150.3	2439 [Nar]	299 [O3]
Tro6-cTnI100	10	39A	SER	3.01	3.46	109.97	299 [O3]	2441 [O2]
Tro6-cTnI100	11	42A	SER	2.61	3.27	125.87	322 [O3]	2420 [O2]
Tro6-cTnI100	12	43A	ALA	3.22	3.68	110.22	325 [Nam]	1718 [Nar]
Tro6-cTnI100	13	46A	LYS	2.95	3.62	124.29	353 [N3]	1738 [Npl]
Tro6-cTnI100	14	46A	LYS	3.21	3.62	106.93	1738 [Npl]	353 [N3]
Tro6-cTnI100	15	136A	ARG	2.17	2.99	139.61	1820 [Npl]	1085 [O2]
Tro6-cTnI100	16	136A	ARG	2.4	3.17	135.04	1083 [Ng+]	1836 [O2]
Tro6-cTnI100	17	137A	GLY	2.92	3.53	121.31	1086 [Nam]	1839 [Npl]
Tro6-cTnI100	18	138A	LYS	2.41	3.35	160.35	1090 [Nam]	1817 [O2]
Tro6-cTnI100	19	139A	PHE	2.17	3.12	159.7	1099 [Nam]	1818 [Nar]
Tro6-cTnI100	20	141A	ARG	2.22	2.91	125.47	1127 [Ng+]	1811 [O3]
Tro6-cTnI100	21	148A	ARG	2.57	3.19	120.45	1188 [Ng+]	1870 [O3]
Tro6-cTnI100	22	193A	LYS	3.27	4.03	134.69	2263 [N2]	1551 [O2]
Tro6-cTnI100	23	194A	ASN	1.79	2.71	153.83	1552 [Nam]	2150 [O3]
Tro6-cTnI100	24	194A	ASN	2.85	3.66	139.8	1557 [Nam]	2263 [N2]
Tro6-cTnI100	25	199A	SER	2.29	2.99	127.24	2340 [Npl]	1592 [O3]
Tro6-cTnI100	26	207A	LYS	2.51	3.42	154.1	1649 [Nam]	2100 [Nar]
Tro6-cTnI100	27	209A	GLU	2.31	3.29	175.94	2103 [Npl]	1675 [O3]

Table A.3. Π-Stack Interaction

Complex_Time point	Index	Residue	AA	Distance	Angle	Offset	Stacking Type	Ligand Atoms
Tro1-cTnI20	1	101A	HIS	3.35	17.41	0.09	P	1730, 1733, 1734, 1735, 1736, 1737
Tro1-cTnI20	2	101A	HIS	3.46	12.96	1.39	P	1729, 1730, 1731, 1732, 1736
Tro1-cTnI60	1	101A	HIS	3.98	29.67	1.6	P	1730, 1733, 1734, 1735, 1736, 1737
Tro1-cTnI60	2	101A	HIS	3.94	29.98	1.52	P	1729, 1730, 1731, 1732, 1736
Tro1-cTnI80	1	101A	HIS	4.08	24.65	0.57	P	1729, 1730, 1731, 1732, 1736
Tro1-cTnI100	1	101A	HIS	3.78	10.75	1.49	P	1729, 1730, 1731, 1732, 1736
Tro3-cTnI20	1	112A	TYR	4.22	19.16	0.82	P	2296, 2297, 2298, 2299, 2303
Tro3-cTnI20	2	112A	TYR	3.97	21.38	0.39	P	2297, 2300, 2301, 2302, 2303, 2304
Tro3-cTnI40	1	112A	TYR	3.91	9.97	0.82	P	2296, 2297, 2298, 2299, 2303
Tro3-cTnI40	2	112A	TYR	4.72	15.79	1.93	P	2297, 2300, 2301, 2302, 2303, 2304
Tro3-cTnI40	3	191A	TRP	4.06	24.22	1.18	P	2237, 2238, 2239, 2240, 2242, 2243
Tro3-cTnI60	1	191A	TRP	5.24	73.53	1.2	T	2237, 2238, 2239, 2240, 2242, 2243
Tro3-cTnI80	1	112A	TYR	4.06	2.25	1.82	P	2297, 2300, 2301, 2302, 2303, 2304
Tro3-cTnI80	2	112A	TYR	3.75	3.62	0.99	P	2296, 2297, 2298, 2299, 2303
Tro3-cTnI80	3	191A	TRP	5.05	81.73	0.89	T	2237, 2238, 2239, 2240, 2242, 2243

Complex_Time point	Index	Residue	AA	Distance	Angle	Offset	Stacking Type	Ligand Atoms
Tro3-cTnI100	1	112A	TYR	3.91	1.31	1.68	P	2296, 2297, 2298, 2299, 2303
Tro3-cTnI100	2	112A	TYR	3.98	3.1	1.76	P	2297, 2300, 2301, 2302, 2303, 2304
Tro4-cTnI40	1	139A	PHE	3.75	15.31	0.18	P	1835, 1836, 1837, 1839, 1840, 1842
Tro4-cTnI60	1	139A	PHE	3.89	2.58	1.56	P	1835, 1836, 1837, 1839, 1840, 1842
Tro4-cTnI80	1	139A	PHE	4.11	7.55	1.79	P	1835, 1836, 1837, 1839, 1840, 1842
Tro4-cTnI100	1	139A	PHE	4.31	7.73	1.94	P	1835, 1836, 1837, 1839, 1840, 1842
Tro6-cTnI40	1	139A	PHE	5.22	72.29	1.49	T	1813, 1814, 1815, 1816, 1818, 1819
Tro6-cTnI80	1	139A	PHE	3.91	10.2	0.68	P	1813, 1814, 1815, 1816, 1818, 1819
Tro6-cTnI60	1	139A	PHE	3.74	15.71	0.75	P	1813, 1814, 1815, 1816, 1818, 1819

Table A.4. Π-Cation Interaction

Complex_Time point	Index	Residue	AA	Distance	Offset	Ligand Group	Ligand Atom
Tro1-cTnI20	1	36A	LYS	4.05	1.79	Aromatic	2303, 2304, 2305, 2307, 2308, 2310
Tro1-cTnI20	2	45A	ARG	3.49	0.46	Aromatic	2403, 2405, 2406, 2407, 2408, 2410
Tro1-cTnI20	3	45A	ARG	3.86	1.64	Aromatic	2402, 2403, 2410, 2411, 2412
Tro1-cTnI40	1	148A	ARG	3.42	0.29	Aromatic	1769, 1770, 1771, 1772, 1776
Tro1-cTnI40	2	162A	ARG	3.76	0.07	Aromatic	1982, 1983, 1984, 1985, 1987, 1988
Tro1-cTnI80	1	20A	ARG	4.25	1.04	Aromatic	2103, 2104, 2105, 2106, 2108, 2109
Tro1-cTnI80	2	74A	ARG	3.67	0.09	Aromatic	1690, 1691, 1692, 1694, 1695, 1697
Tro1-cTnI80	3	162A	ARG	3.97	1.39	Aromatic	1982, 1983, 1984, 1985, 1987, 1988
Tro1-cTnI80	4	177A	LYS	4.94	1.27	Aromatic	1828, 1829, 1830, 1831, 1833, 1834
Tro1-cTnI80	5	192A	ARG	4.1	1.32	Aromatic	1867, 1868, 1869, 1870, 1872, 1873
Tro1-cTnI100	1	20A	ARG	3.61	1.13	Aromatic	2103, 2104, 2105, 2106, 2108, 2109
Tro1-cTnI100	2	37A	LYS	5.95	0.5	Aromatic	2244, 2245, 2246, 2247, 2249, 2250
Tro1-cTnI100	3	74A	ARG	4.14	1.02	Aromatic	1690, 1691, 1692, 1694, 1695, 1697
Tro1-cTnI100	4	162A	ARG	3.7	0.58	Aromatic	1982, 1983, 1984, 1985, 1987, 1988
Tro1-cTnI100	5	192A	ARG	3.84	0.94	Aromatic	1867, 1868, 1869, 1870, 1872, 1873
Tro2-cTnI20	1	37A	LYS	4.04	1.57	Aromatic	1849, 1850, 1851, 1852, 1854, 1855
Tro2-cTnI20	2	50A	LYS	4.04	1.81	Aromatic	1828, 1829, 1830, 1831, 1835
Tro2-cTnI60	1	162A	ARG	3.88	1.78	Aromatic	2066, 2067, 2068, 2070, 2071, 2073
Tro2-cTnI80	1	37A	LYS	4.56	0.24	Aromatic	1849, 1850, 1851, 1852, 1854, 1855
Tro2-cTnI80	2	141A	ARG	3.62	1.15	Aromatic	1690, 1691, 1692, 1693, 1695, 1696
Tro2-cTnI80	3	162A	ARG	3.5	0.91	Aromatic	2066, 2067, 2068, 2070, 2071, 2073
Tro2-cTnI100	1	141A	ARG	4.5	1.57	Aromatic	1709, 1710, 1711, 1712, 1714, 1715
Tro2-cTnI100	2	162A	ARG	3.56	0.85	Aromatic	2066, 2067, 2068, 2070, 2071, 2073
Tro2-cTnI100	3	204A	ARG	3.51	0.9	Aromatic	2450, 2451, 2452, 2453, 2457
Tro2-cTnI100	4	204A	ARG	3.57	1.11	Aromatic	2451, 2454, 2455, 2456, 2457, 2458
Tro3-cTnI20	1	120A	LYS	4.32	1.75	Aromatic	1834, 1835, 1842, 1843, 1844
Tro3-cTnI40	1	21A	ARG	3.81	1.47	Aromatic	2256, 2257, 2258, 2259, 2263
Tro3-cTnI40	2	46A	LYS	3.59	1.98	Aromatic	2033, 2034, 2041, 2042, 2043
Tro3-cTnI40	3	69A	ARG	3.83	0.17	Aromatic	2257, 2260, 2261, 2262, 2263, 2264

Complex_Time point	Index	Residue	AA	Distance	Offset	Ligand Group	Ligand Atom
Tro3-cTnI60	1	21A	ARG	3.87	1.68	Aromatic	2257, 2260, 2261, 2262, 2263, 2264
Tro3-cTnI60	2	140A	LYS	5.17	1.36	Aromatic	2479, 2482, 2483, 2484, 2485, 2486
Tro3-cTnI60	3	140A	LYS	5.25	1.6	Aromatic	2478, 2479, 2480, 2481, 2485
Tro3-cTnI100	1	21A	ARG	3.33	0.64	Aromatic	2256, 2257, 2258, 2259, 2263
Tro3-cTnI100	2	37A	LYS	5.81	1.52	Aromatic	2479, 2482, 2483, 2484, 2485, 2486
Tro3-cTnI100	3	37A	LYS	5.81	1.79	Aromatic	2478, 2479, 2480, 2481, 2485
Tro3-cTnI100	4	131A	LYS	5.77	1.32	Aromatic	1711, 1712, 1713, 1715, 1716, 1718
Tro3-cTnI0	1	141A	ARG	5.91	1.46	Aromatic	2497, 2498, 2500, 2502, 2503, 2507
Tro4-cTnI20	1	204A	ARG	3.54	0.5	Aromatic	2473, 2474, 2475, 2476, 2480
Tro4-cTnI40	1	204A	ARG	3.69	1.23	Aromatic	2473, 2474, 2475, 2476, 2480
Tro4-cTnI40	2	204A	ARG	3.86	1.8	Aromatic	2474, 2477, 2478, 2479, 2480, 2481
Tro4-cTnI60	1	10A	ARG	4.27	1.75	Aromatic	1976, 1977, 1978, 1979, 1983
Tro4-cTnI60	2	204A	ARG	4.13	1.04	Aromatic	2473, 2474, 2475, 2476, 2480
Tro4-cTnI80	1	10A	ARG	4.01	1.06	Aromatic	1997, 1998, 1999, 2000, 2002, 2003
Tro4-cTnI80	2	37A	LYS	4.41	0.73	Aromatic	1997, 1998, 1999, 2000, 2002, 2003
Tro4-cTnI80	3	204A	ARG	3.45	0.37	Aromatic	2473, 2474, 2475, 2476, 2480
Tro4-cTnI100	1	10A	ARG	4.62	0.62	Aromatic	1997, 1998, 1999, 2000, 2002, 2003
Tro4-cTnI100	2	37A	LYS	4.52	0.12	Aromatic	1997, 1998, 1999, 2000, 2002, 2003
Tro4-cTnI100	3	204A	ARG	3.44	0.25	Aromatic	2473, 2474, 2475, 2476, 2480
Tro5-cTnI20	1	140A	LYS	3.96	1.8	Aromatic	2174, 2175, 2176, 2177, 2179, 2180
Tro5-cTnI20	2	204A	ARG	3.76	1.34	Aromatic	2333, 2334, 2335, 2336, 2340
Tro5-cTnI40	1	204A	ARG	3.9	1.48	Aromatic	2354, 2355, 2356, 2357, 2359, 2360
Tro5-cTnI80	1	20A	ARG	3.45	1.09	Aromatic	1709, 1710, 1711, 1712, 1716
Tro5-cTnI80	2	20A	ARG	3.39	1.04	Aromatic	1710, 1713, 1714, 1715, 1716, 1717
Tro5-cTnI80	3	141A	ARG	3.94	0.92	Aromatic	2174, 2175, 2176, 2177, 2179, 2180
Tro5-cTnI80	4	162A	ARG	3.94	0.7	Aromatic	1730, 1731, 1732, 1733, 1737
Tro5-cTnI100	1	20A	ARG	3.59	1.19	Aromatic	1709, 1710, 1711, 1712, 1716
Tro5-cTnI100	2	20A	ARG	3.78	1.7	Aromatic	1710, 1713, 1714, 1715, 1716, 1717
Tro5-cTnI100	3	162A	ARG	3.56	0.7	Aromatic	1730, 1731, 1732, 1733, 1737
Tro5-cTnI100	4	204A	ARG	3.86	0.69	Aromatic	2354, 2355, 2356, 2357, 2359, 2360
Tro6-cTnI0	1	206A	LYS	4.98	0.96	Aromatic	2806, 2807, 2809, 2811, 2813, 2815
Tro6-cTnI20	1	141A	ARG	3.74	0.84	Aromatic	1813, 1814, 1815, 1816, 1818, 1819
Tro6-cTnI20	2	183A	LYS	3.86	1.79	Aromatic	2156, 2157, 2164, 2165, 2166
Tro6-cTnI20	3	186A	ARG	3.84	0.92	Aromatic	2198, 2199, 2200, 2202, 2203, 2205
Tro6-cTnI40	1	36A	LYS	4.96	1.36	Aromatic	1914, 1915, 1916, 1917, 1919, 1920
Tro6-cTnI80	1	148A	ARG	4.03	1.92	Aromatic	1873, 1876, 1877, 1878, 1879, 1880
Tro6-cTnI80	2	186A	ARG	3.89	1.63	Aromatic	2198, 2199, 2200, 2202, 2203, 2205
Tro6-cTnI60	1	45A	ARG	4.2	1.89	Aromatic	2413, 2414, 2421, 2422, 2423
Tro6-cTnI60	2	204A	ARG	3.74	1.04	Aromatic	2075, 2076, 2077, 2078, 2080, 2081
Tro6-cTnI100	1	36A	LYS	5.37	1.74	Aromatic	1914, 1915, 1916, 1917, 1919, 1920
Tro6-cTnI100	2	148A	ARG	3.39	0.3	Aromatic	1872, 1873, 1874, 1875, 1879
Tro6-cTnI100	3	148A	ARG	4.05	1.95	Aromatic	1873, 1876, 1877, 1878, 1879, 1880
Tro6-cTnI100	4	186A	ARG	3.93	1.66	Aromatic	2198, 2199, 2200, 2202, 2203, 2205

Complex_Time point	Index	Residue	AA	Distance	Offset	Ligand Group	Ligand Atom
Tro6-cTnI100	5	207A	LYS	5.68	1.93	Aromatic	2094, 2095, 2096, 2097, 2101

Table A.5. Salt Bridge Interaction

Complex_Time point	Index	Residue	AA	Distance	Ligand Group	Ligand Atoms
Tro1-cTnI_20	1	20A	ARG	5.04	Phosphate	2155, 2155, 2154, 2156, 2157, 2158
Tro1-cTnI_20	2	21A	ARG	4.4	Phosphate	2095, 2095, 2096, 2097, 2098, 2094
Tro1-cTnI_20	3	21A	ARG	4.87	Phosphate	2054, 2054, 2056, 2057, 2053, 2055
Tro1-cTnI_20	4	36A	LYS	3.96	Phosphate	2276, 2276, 2275, 2277, 2278, 2279
Tro1-cTnI_20	5	36A	LYS	4.24	Phosphate	2295, 2295, 2296, 2297, 2298, 2294
Tro1-cTnI_20	6	101A	HIS	5.23	Phosphate	1721, 1721, 1720, 1722, 1723, 1724
Tro1-cTnI_20	7	170A	ARG	4.72	Phosphate	1916, 1916, 1915, 1917, 1918, 1919
Tro1-cTnI_20	8	170A	ARG	4.13	Phosphate	1936, 1936, 1937, 1938, 1939, 1935
Tro1-cTnI_20	9	174A	LYS	3.81	Phosphate	1820, 1820, 1819, 1821, 1822, 1823
Tro1-cTnI_20	10	177A	LYS	3.58	Phosphate	1820, 1820, 1819, 1821, 1822, 1823
Tro1-cTnI_20	11	177A	LYS	3.99	Phosphate	1839, 1839, 1838, 1840, 1841,
Tro1-cTnI_40	1	20A	ARG	4.62	Phosphate	2155, 2155, 2154, 2156, 2157, 2158
Tro1-cTnI_40	2	21A	ARG	4.51	Phosphate	2095, 2095, 2094, 2096, 2097, 2098
Tro1-cTnI_40	3	21A	ARG	4.74	Phosphate	2054, 2054, 2053, 2056, 2057, 2055
Tro1-cTnI_40	4	34A	HIS	4.4	Phosphate	2236, 2236, 2235, 2237, 2238, 2239
Tro1-cTnI_40	5	36A	LYS	4.17	Phosphate	2276, 2276, 2275, 2277, 2278, 2279
Tro1-cTnI_40	6	36A	LYS	4.57	Phosphate	2295, 2295, 2294, 2296, 2297, 2298
Tro1-cTnI_40	7	45A	ARG	4.4	Phosphate	2033, 2033, 2032, 2034, 2035, 2036
Tro1-cTnI_40	8	98A	ARG	4.56	Phosphate	1742, 1742, 1744, 1745, 1741, 1743
Tro1-cTnI_40	9	162A	ARG	4.37	Phosphate	1955, 1955, 1954, 1956, 1957, 1958
Tro1-cTnI_40	10	162A	ARG	4.92	Phosphate	1974, 1974, 1973, 1976, 1977, 1975
Tro1-cTnI_40	11	170A	ARG	4.4	Phosphate	1916, 1916, 1915, 1917, 1918, 1919
Tro1-cTnI_40	12	170A	ARG	4.75	Phosphate	1897, 1897, 1896, 1898, 1899, 1900
Tro1-cTnI_40	13	170A	ARG	4.58	Phosphate	1936, 1936, 1937, 1938, 1939, 1935
Tro1-cTnI_40	14	177A	LYS	5.4	Phosphate	1820, 1820, 1819, 1821, 1822, 1823
Tro1-cTnI_40	15	177A	LYS	3.93	Phosphate	1839, 1839, 1840, 1841, 1842, 1838
Tro1-cTnI_40	16	206A	LYS	4.19	Phosphate	1974, 1974, 1973, 1976, 1977, 1975
Tro1-cTnI_60	1	20A	ARG	4.81	Phosphate	2114, 2114, 2113, 2115, 2116, 2117
Tro1-cTnI_60	2	20A	ARG	5.11	Phosphate	2133, 2133, 2136, 2132, 2134, 2135
Tro1-cTnI_60	3	21A	ARG	4.84	Phosphate	2095, 2095, 2096, 2097, 2098, 2094
Tro1-cTnI_60	4	21A	ARG	4.92	Phosphate	2074, 2074, 2073, 2075, 2076, 2077
Tro1-cTnI_60	5	21A	ARG	4.79	Phosphate	2054, 2054, 2056, 2057, 2053, 2055
Tro1-cTnI_60	6	22A	ARG	4.88	Phosphate	2074, 2074, 2073, 2075, 2076, 2077
Tro1-cTnI_60	7	36A	LYS	3.96	Phosphate	2276, 2276, 2275, 2277, 2278, 2279
Tro1-cTnI_60	8	36A	LYS	4.32	Phosphate	2295, 2295, 2294, 2296, 2297, 2298
Tro1-cTnI_60	9	37A	LYS	3.59	Phosphate	2255, 2255, 2256, 2257, 2258, 2254
Tro1-cTnI_60	10	37A	LYS	5.2	Phosphate	2276, 2276, 2275, 2277, 2278, 2279
Tro1-cTnI_60	11	45A	ARG	4.17	Phosphate	2033, 2033, 2032, 2034, 2035, 2036

Complex_Time point	Index	Residue	AA	Distance	Ligand Group	Ligand Atoms
Tro1-cTnI_60	12	69A	ARG	4.84	Phosphate	1721, 1721, 1720, 1722, 1723, 1724
Tro1-cTnI_60	13	69A	ARG	4.23	Phosphate	1702, 1702, 1704, 1705, 1701, 1703
Tro1-cTnI_60	14	101A	HIS	4.84	Phosphate	1721, 1721, 1720, 1722, 1723, 1724
Tro1-cTnI_60	15	162A	ARG	4.45	Phosphate	1955, 1955, 1954, 1956, 1957, 1958
Tro1-cTnI_60	16	162A	ARG	5.22	Phosphate	1974, 1974, 1976, 1977, 1973, 1975
Tro1-cTnI_60	17	170A	ARG	4.25	Phosphate	1916, 1916, 1915, 1917, 1918, 1919
Tro1-cTnI_60	18	170A	ARG	4.69	Phosphate	1897, 1897, 1896, 1898, 1899, 1900
Tro1-cTnI_60	19	170A	ARG	4.6	Phosphate	1936, 1936, 1937, 1938, 1939, 1935
Tro1-cTnI_60	20	177A	LYS	3.54	Phosphate	1820, 1820, 1819, 1821, 1822, 1823
Tro1-cTnI_60	21	177A	LYS	5.44	Phosphate	1839, 1839, 1840, 1841, 1842, 1838
Tro1-cTnI_60	22	192A	ARG	4.48	Phosphate	1859, 1859, 1858, 1860, 1861, 1862
Tro1-cTnI_60	23	206A	LYS	3.77	Phosphate	1974, 1974, 1976, 1977, 1973, 1975
Tro1-cTnI_80	1	20A	ARG	4.57	Phosphate	2155, 2155, 2154, 2156, 2157, 2158
Tro1-cTnI_80	2	21A	ARG	4.76	Phosphate	2095, 2095, 2096, 2097, 2098, 2094
Tro1-cTnI_80	3	21A	ARG	4.96	Phosphate	2074, 2074, 2073, 2075, 2076, 2077
Tro1-cTnI_80	4	21A	ARG	4.82	Phosphate	2054, 2054, 2053, 2056, 2057, 2055
Tro1-cTnI_80	5	22A	ARG	4.42	Phosphate	2074, 2074, 2073, 2075, 2076, 2077
Tro1-cTnI_80	6	36A	LYS	3.68	Phosphate	2276, 2276, 2275, 2277, 2278, 2279
Tro1-cTnI_80	7	36A	LYS	4.08	Phosphate	2295, 2295, 2296, 2297, 2298, 2294
Tro1-cTnI_80	8	45A	ARG	4.32	Phosphate	2033, 2033, 2032, 2034, 2035, 2036
Tro1-cTnI_80	9	69A	ARG	4.52	Phosphate	1702, 1702, 1701, 1704, 1705, 1703
Tro1-cTnI_80	10	69A	ARG	4.54	Phosphate	1721, 1721, 1720, 1722, 1723, 1724
Tro1-cTnI_80	11	101A	HIS	5.29	Phosphate	1702, 1702, 1701, 1704, 1705, 1703
Tro1-cTnI_80	12	101A	HIS	4.95	Phosphate	1721, 1721, 1720, 1722, 1723, 1724
Tro1-cTnI_80	13	162A	ARG	4.14	Phosphate	1955, 1955, 1954, 1956, 1957, 1958
Tro1-cTnI_80	14	170A	ARG	4.52	Phosphate	1916, 1916, 1915, 1917, 1918, 1919
Tro1-cTnI_80	15	170A	ARG	4.53	Phosphate	1897, 1897, 1896, 1898, 1899, 1900
Tro1-cTnI_80	16	170A	ARG	4.04	Phosphate	1936, 1936, 1935, 1937, 1938, 1939
Tro1-cTnI_80	17	177A	LYS	3.88	Phosphate	1820, 1820, 1819, 1821, 1822, 1823
Tro1-cTnI_80	18	192A	ARG	5.01	Phosphate	1878, 1878, 1877, 1880, 1881, 1879
Tro1-cTnI_100	1	20A	ARG	4.95	Phosphate	2133, 2133, 2132, 2136, 2134, 2135
Tro1-cTnI_100	2	21A	ARG	4.39	Phosphate	2095, 2095, 2096, 2097, 2098, 2094
Tro1-cTnI_100	3	21A	ARG	5.07	Phosphate	2074, 2074, 2073, 2075, 2076, 2077
Tro1-cTnI_100	4	21A	ARG	4.81	Phosphate	2054, 2054, 2053, 2056, 2057, 2055
Tro1-cTnI_100	5	36A	LYS	3.79	Phosphate	2295, 2295, 2294, 2296, 2297, 2298
Tro1-cTnI_100	6	37A	LYS	3.9	Phosphate	2255, 2255, 2254, 2256, 2257, 2258
Tro1-cTnI_100	7	37A	LYS	3.68	Phosphate	2276, 2276, 2275, 2277, 2278, 2279
Tro1-cTnI_100	8	45A	ARG	4.31	Phosphate	2033, 2033, 2032, 2034, 2035, 2036
Tro1-cTnI_100	9	69A	ARG	4.57	Phosphate	1702, 1702, 1701, 1704, 1705, 1703
Tro1-cTnI_100	10	69A	ARG	5.02	Phosphate	1721, 1721, 1720, 1722, 1723, 1724
Tro1-cTnI_100	11	98A	ARG	4.16	Phosphate	1761, 1761, 1760, 1762, 1763, 1764
Tro1-cTnI_100	12	98A	ARG	4.86	Phosphate	1742, 1742, 1744, 1745, 1741, 1743
Tro1-cTnI_100	13	101A	HIS	4.61	Phosphate	1702, 1702, 1701, 1704, 1705, 1703
Tro1-cTnI_100	14	101A	HIS	5.3	Phosphate	1721, 1721, 1720, 1722, 1723, 1724

Complex_Time point	Index	Residue	AA	Distance	Ligand Group	Ligand Atoms
Tro1-cTnI_100	15	162A	ARG	4.42	Phosphate	1955, 1955, 1954, 1956, 1957, 1958
Tro1-cTnI_100	16	162A	ARG	5	Phosphate	1974, 1974, 1976, 1977, 1973, 1975
Tro1-cTnI_100	17	170A	ARG	4.99	Phosphate	1897, 1897, 1896, 1898, 1899, 1900
Tro1-cTnI_100	18	170A	ARG	4.77	Phosphate	1916, 1916, 1915, 1917, 1918, 1919
Tro1-cTnI_100	19	170A	ARG	4.73	Phosphate	1936, 1936, 1937, 1938, 1939, 1935
Tro1-cTnI_100	20	177A	LYS	3.96	Phosphate	1820, 1820, 1819, 1821, 1822, 1823
Tro1-cTnI_100	21	186A	ARG	5.22	Phosphate	1820, 1820, 1819, 1821, 1822, 1823
Tro1-cTnI_100	22	192A	ARG	4.8	Phosphate	1878, 1878, 1880, 1881, 1877, 1879
Tro1-cTnI_0	1	36A	LYS	4.25	Phosphate	3125, 3125, 3124, 3128, 3126, 3127
Tro1-cTnI_0	2	36A	LYS	4.66	Phosphate	3063, 3063, 3062, 3064, 3065, 3066
Tro1-cTnI_0	3	45A	ARG	4.7	Phosphate	3219, 3219, 3218, 3220, 3221, 3222
Tro1-cTnI_0	4	170A	ARG	3.85	Phosphate	2502, 2502, 2504, 2505, 2501, 2503
Tro1-cTnI_0	5	170A	ARG	4.82	Phosphate	2472, 2472, 2471, 2473, 2474, 2475
Tro1-cTnI_0	6	170A	ARG	4.04	Phosphate	2534, 2534, 2536, 2537, 2533, 2535
Tro1-cTnI_0	7	177A	LYS	4.03	Phosphate	2412, 2412, 2411, 2413, 2414, 2415
Tro2-cTnI_0	1	46A	LYS	4.35	Phosphate	2349, 2349, 2348, 2352, 2350, 2351
Tro2-cTnI_0	2	50A	LYS	3.14	Phosphate	2319, 2319, 2320, 2321, 2322, 2318
Tro2-cTnI_0	3	140A	LYS	2.95	Phosphate	2224, 2224, 2225, 2226, 2227, 2223
Tro2-cTnI_0	4	141A	ARG	4.05	Phosphate	2194, 2194, 2193, 2195, 2196, 2197
Tro2-cTnI_0	5	146A	ARG	4.53	Phosphate	2444, 2444, 2443, 2445, 2446, 2447
Tro2-cTnI_0	6	146A	ARG	4.78	Phosphate	2476, 2476, 2475, 2477, 2478, 2479
Tro2-cTnI_0	7	148A	ARG	4.16	Phosphate	2289, 2289, 2288, 2290, 2291, 2292
Tro2-cTnI_0	8	204A	ARG	4.33	Phosphate	3194, 3194, 3193, 3195, 3196, 3197
Tro2-cTnI_0	9	206A	LYS	5.23	Phosphate	3162, 3162, 3161, 3163, 3164, 3165
Tro2-cTnI_20	1	37A	LYS	3.62	Phosphate	1841, 1841, 1840, 1842, 1843, 1844
Tro2-cTnI_20	2	46A	LYS	3.71	Phosphate	1820, 1820, 1819, 1821, 1822, 1823
Tro2-cTnI_20	3	50A	LYS	4.09	Phosphate	1801, 1801, 1800, 1802, 1803, 1804
Tro2-cTnI_20	4	148A	ARG	5	Phosphate	1782, 1782, 1784, 1785, 1781, 1783
Tro2-cTnI_20	5	204A	ARG	4.64	Phosphate	2338, 2338, 2337, 2339, 2340, 2341
Tro2-cTnI_20	6	204A	ARG	5.21	Phosphate	2358, 2358, 2360, 2361, 2357, 2359
Tro2-cTnI_20	7	206A	LYS	3.72	Phosphate	2338, 2338, 2337, 2339, 2340, 2341
Tro2-cTnI_20	8	207A	LYS	4.3	Phosphate	2318, 2318, 2320, 2321, 2317, 2319
Tro2-cTnI_40	1	37A	LYS	4.04	Phosphate	1841, 1841, 1840, 1842, 1843, 1844
Tro2-cTnI_40	2	46A	LYS	3.95	Phosphate	1820, 1820, 1819, 1821, 1822, 1823
Tro2-cTnI_40	3	50A	LYS	3.73	Phosphate	1801, 1801, 1800, 1802, 1803, 1804
Tro2-cTnI_40	4	140A	LYS	4.13	Phosphate	1739, 1739, 1738, 1740, 1741, 1742
Tro2-cTnI_40	5	140A	LYS	3.79	Phosphate	1720, 1720, 1721, 1722, 1723, 1719
Tro2-cTnI_40	6	145A	ARG	5.38	Phosphate	1761, 1761, 1760, 1762, 1763, 1764
Tro2-cTnI_40	7	146A	ARG	5.39	Phosphate	1882, 1882, 1881, 1883, 1884, 1885
Tro2-cTnI_40	8	148A	ARG	4.76	Phosphate	1782, 1782, 1784, 1785, 1781, 1783
Tro2-cTnI_40	9	148A	ARG	4.76	Phosphate	1761, 1761, 1760, 1762, 1763, 1764
Tro2-cTnI_40	10	204A	ARG	4.35	Phosphate	2358, 2358, 2360, 2361, 2357, 2359
Tro2-cTnI_40	11	205A	LYS	4.18	Phosphate	2442, 2442, 2441, 2443, 2444, 2445
Tro2-cTnI_60	1	37A	LYS	4.07	Phosphate	1841, 1841, 1840, 1842, 1843, 1844

Complex_Time point	Index	Residue	AA	Distance	Ligand Group	Ligand Atoms
Tro2-cTnI_60	2	46A	LYS	3.85	Phosphate	1820, 1820, 1819, 1821, 1822, 1823
Tro2-cTnI_60	3	50A	LYS	4.95	Phosphate	1801, 1801, 1800, 1802, 1803, 1804
Tro2-cTnI_60	4	136A	ARG	4.45	Phosphate	1739, 1739, 1738, 1740, 1741, 1742
Tro2-cTnI_60	5	145A	ARG	5.23	Phosphate	1739, 1739, 1738, 1740, 1741, 1742
Tro2-cTnI_60	6	146A	ARG	4.67	Phosphate	1902, 1902, 1904, 1905, 1901, 1903
Tro2-cTnI_60	7	162A	ARG	4.19	Phosphate	2058, 2058, 2057, 2059, 2060, 2061
Tro2-cTnI_60	8	204A	ARG	5.07	Phosphate	2420, 2420, 2419, 2421, 2422, 2423
Tro2-cTnI_60	9	204A	ARG	4.68	Phosphate	2400, 2400, 2401, 2402, 2403, 2399
Tro2-cTnI_60	10	204A	ARG	5.02	Phosphate	2442, 2442, 2441, 2443, 2444, 2445
Tro2-cTnI_60	11	205A	LYS	3.68	Phosphate	2442, 2442, 2441, 2443, 2444, 2445
Tro2-cTnI_80	1	34A	HIS	4.9	Phosphate	1782, 1782, 1784, 1785, 1781, 1783
Tro2-cTnI_80	2	37A	LYS	3.79	Phosphate	1820, 1820, 1819, 1821, 1822, 1823
Tro2-cTnI_80	3	37A	LYS	3.96	Phosphate	1841, 1841, 1840, 1842, 1843, 1844
Tro2-cTnI_80	4	46A	LYS	4.07	Phosphate	1820, 1820, 1819, 1821, 1822, 1823
Tro2-cTnI_80	5	50A	LYS	3.89	Phosphate	1801, 1801, 1800, 1802, 1803, 1804
Tro2-cTnI_80	6	141A	ARG	4.91	Phosphate	1701, 1701, 1704, 1700, 1702, 1703
Tro2-cTnI_80	7	145A	ARG	4.87	Phosphate	1782, 1782, 1784, 1785, 1781, 1783
Tro2-cTnI_80	8	145A	ARG	4.09	Phosphate	1761, 1761, 1760, 1762, 1763, 1764
Tro2-cTnI_80	9	146A	ARG	4.84	Phosphate	1902, 1902, 1904, 1905, 1901, 1903
Tro2-cTnI_80	10	148A	ARG	4.22	Phosphate	1782, 1782, 1784, 1785, 1781, 1783
Tro2-cTnI_80	11	162A	ARG	4.33	Phosphate	2058, 2058, 2057, 2059, 2060, 2061
Tro2-cTnI_80	12	162A	ARG	4.84	Phosphate	2039, 2039, 2038, 2040, 2041, 2042
Tro2-cTnI_80	13	204A	ARG	4.73	Phosphate	2420, 2420, 2419, 2421, 2422, 2423
Tro2-cTnI_80	14	204A	ARG	4.46	Phosphate	2380, 2380, 2379, 2381, 2382, 2383
Tro2-cTnI_100	1	37A	LYS	4.01	Phosphate	1841, 1841, 1840, 1842, 1843, 1844
Tro2-cTnI_100	2	37A	LYS	4	Phosphate	1820, 1820, 1819, 1821, 1822, 1823
Tro2-cTnI_100	3	46A	LYS	3.89	Phosphate	1820, 1820, 1819, 1821, 1822, 1823
Tro2-cTnI_100	4	50A	LYS	4.14	Phosphate	1801, 1801, 1800, 1802, 1803, 1804
Tro2-cTnI_100	5	136A	ARG	4.82	Phosphate	1739, 1739, 1738, 1740, 1741, 1742
Tro2-cTnI_100	6	140A	LYS	3.58	Phosphate	1720, 1720, 1719, 1721, 1722, 1723
Tro2-cTnI_100	7	148A	ARG	4.1	Phosphate	1782, 1782, 1781, 1784, 1785, 1783
Tro2-cTnI_100	8	162A	ARG	4.75	Phosphate	2039, 2039, 2038, 2040, 2041, 2042
Tro2-cTnI_100	9	162A	ARG	4.71	Phosphate	2058, 2058, 2057, 2059, 2060, 2061
Tro2-cTnI_100	10	204A	ARG	3.99	Phosphate	2442, 2442, 2441, 2443, 2444, 2445
Tro2-cTnI_100	11	205A	LYS	3.76	Phosphate	2176, 2176, 2177, 2178, 2179, 2175
Tro2-cTnI_100	12	207A	LYS	4.09	Phosphate	2318, 2318, 2320, 2321, 2317, 2319
Tro4-cTnI_20	1	37A	LYS	4.07	Phosphate	2008, 2008, 2009, 2010, 2011, 2007
Tro4-cTnI_20	2	46A	LYS	3.87	Phosphate	2268, 2268, 2267, 2269, 2270, 2271
Tro4-cTnI_20	3	50A	LYS	4.04	Phosphate	2288, 2288, 2289, 2290, 2291, 2287
Tro4-cTnI_20	4	140A	LYS	3.56	Phosphate	1847, 1847, 1848, 1849, 1850, 1846
Tro4-cTnI_20	5	146A	ARG	4.6	Phosphate	2348, 2348, 2347, 2349, 2350, 2351
Tro4-cTnI_20	6	146A	ARG	4.37	Phosphate	2329, 2329, 2328, 2330, 2331, 2332
Tro4-cTnI_20	7	148A	ARG	4.83	Phosphate	2329, 2329, 2328, 2330, 2331, 2332
Tro4-cTnI_20	8	204A	ARG	5.48	Phosphate	2465, 2465, 2464, 2466, 2467, 2468

Complex_Time point	Index	Residue	AA	Distance	Ligand Group	Ligand Atoms
Tro4-cTnI_40	1	37A	LYS	5.35	Phosphate	1989, 1989, 1992, 1988, 1990, 1991
Tro4-cTnI_40	2	38A	LYS	4.04	Phosphate	1928, 1928, 1929, 1930, 1931, 1927
Tro4-cTnI_40	3	46A	LYS	3.83	Phosphate	2268, 2268, 2267, 2269, 2270, 2271
Tro4-cTnI_40	4	50A	LYS	3.72	Phosphate	2288, 2288, 2287, 2289, 2290, 2291
Tro4-cTnI_40	5	140A	LYS	3.65	Phosphate	1847, 1847, 1846, 1848, 1849, 1850
Tro4-cTnI_40	6	146A	ARG	4.15	Phosphate	2348, 2348, 2347, 2349, 2350, 2351
Tro4-cTnI_40	7	146A	ARG	5.09	Phosphate	2329, 2329, 2328, 2330, 2331, 2332
Tro4-cTnI_40	8	148A	ARG	4.57	Phosphate	2329, 2329, 2328, 2330, 2331, 2332
Tro4-cTnI_60	1	34A	HIS	4.21	Phosphate	1909, 1909, 1912, 1908, 1910, 1911
Tro4-cTnI_60	2	40A	LYS	3.49	Phosphate	2249, 2249, 2248, 2250, 2251, 2252
Tro4-cTnI_60	3	46A	LYS	4.82	Phosphate	2288, 2288, 2289, 2290, 2291, 2287
Tro4-cTnI_60	4	50A	LYS	3.77	Phosphate	2288, 2288, 2289, 2290, 2291, 2287
Tro4-cTnI_60	5	140A	LYS	4.01	Phosphate	1847, 1847, 1846, 1848, 1849, 1850
Tro4-cTnI_60	6	146A	ARG	4.32	Phosphate	2348, 2348, 2347, 2349, 2350, 2351
Tro4-cTnI_60	7	146A	ARG	5.05	Phosphate	2329, 2329, 2328, 2330, 2331, 2332
Tro4-cTnI_60	8	148A	ARG	4.84	Phosphate	2329, 2329, 2328, 2330, 2331, 2332
Tro4-cTnI_60	9	204A	ARG	4.13	Phosphate	2465, 2465, 2464, 2466, 2467, 2468
Tro4-cTnI_80	1	10A	ARG	4.57	Phosphate	1928, 1928, 1927, 1929, 1930, 1931
Tro4-cTnI_80	2	36A	LYS	5.04	Phosphate	2008, 2008, 2009, 2010, 2011, 2007
Tro4-cTnI_80	3	38A	LYS	5.16	Phosphate	2186, 2186, 2185, 2187, 2188, 2189
Tro4-cTnI_80	4	50A	LYS	3.83	Phosphate	2288, 2288, 2287, 2289, 2290, 2291
Tro4-cTnI_80	5	140A	LYS	3.9	Phosphate	1847, 1847, 1848, 1849, 1850, 1846
Tro4-cTnI_80	6	141A	ARG	5.03	Phosphate	1805, 1805, 1804, 1808, 1806, 1807
Tro4-cTnI_80	7	146A	ARG	4.86	Phosphate	2348, 2348, 2347, 2349, 2350, 2351
Tro4-cTnI_80	8	146A	ARG	4.73	Phosphate	2329, 2329, 2328, 2330, 2331, 2332
Tro4-cTnI_80	9	148A	ARG	5.14	Phosphate	2329, 2329, 2328, 2330, 2331, 2332
Tro4-cTnI_80	10	204A	ARG	5.38	Phosphate	2465, 2465, 2464, 2466, 2467, 2468
Tro4-cTnI_100	1	10A	ARG	5.03	Phosphate	1989, 1989, 1988, 1992, 1990, 1991
Tro4-cTnI_100	2	38A	LYS	3.53	Phosphate	2186, 2186, 2185, 2187, 2188, 2189
Tro4-cTnI_100	3	38A	LYS	4	Phosphate	2166, 2166, 2168, 2169, 2165, 2167
Tro4-cTnI_100	4	46A	LYS	5.04	Phosphate	2288, 2288, 2289, 2290, 2291, 2287
Tro4-cTnI_100	5	50A	LYS	3.8	Phosphate	2288, 2288, 2289, 2290, 2291, 2287
Tro4-cTnI_100	6	140A	LYS	3.75	Phosphate	1847, 1847, 1848, 1849, 1850, 1846
Tro4-cTnI_100	7	146A	ARG	4.54	Phosphate	2348, 2348, 2347, 2349, 2350, 2351
Tro4-cTnI_100	8	146A	ARG	4.61	Phosphate	2329, 2329, 2328, 2330, 2331, 2332
Tro4-cTnI_100	9	204A	ARG	4.54	Phosphate	2465, 2465, 2464, 2466, 2467, 2468
Tro4-cTnI_0	1	37A	LYS	3.1	Phosphate	2544, 2544, 2543, 2545, 2546, 2547
Tro4-cTnI_0	2	46A	LYS	4.82	Phosphate	3046, 3046, 3048, 3049, 3045, 3047
Tro4-cTnI_0	3	46A	LYS	3.23	Phosphate	3016, 3016, 3015, 3017, 3018, 3019
Tro4-cTnI_0	4	50A	LYS	3.06	Phosphate	3078, 3078, 3077, 3080, 3081, 3079
Tro4-cTnI_0	5	50A	LYS	4.45	Phosphate	3046, 3046, 3048, 3049, 3045, 3047
Tro4-cTnI_0	6	140A	LYS	3.92	Phosphate	2389, 2389, 2388, 2392, 2390, 2391
Tro4-cTnI_0	7	146A	ARG	3.96	Phosphate	3141, 3141, 3140, 3144, 3142, 3143
Tro4-cTnI_0	8	148A	ARG	4.07	Phosphate	3111, 3111, 3112, 3113, 3114, 3110

Complex_Time point	Index	Residue	AA	Distance	Ligand Group	Ligand Atoms
Tro4-cTnI_0	9	207A	LYS	3.54	Phosphate	3357, 3357, 3356, 3360, 3358, 3359
Tro3-cTnI_20	1	38A	LYS	4.19	Phosphate	2005, 2005, 2008, 2004, 2006, 2007
Tro3-cTnI_20	2	38A	LYS	3.91	Phosphate	1986, 1986, 1985, 1987, 1988, 1989
Tro3-cTnI_20	3	40A	LYS	5.02	Phosphate	2025, 2025, 2024, 2026, 2027, 2028
Tro3-cTnI_20	4	124A	GLU	5.03	Guanidine	1836, 1837, 1839
Tro3-cTnI_20	5	140A	LYS	4.03	Phosphate	2470, 2470, 2469, 2472, 2473, 2471
Tro3-cTnI_20	6	140A	LYS	5.32	Phosphate	2448, 2448, 2449, 2450, 2451, 2447
Tro3-cTnI_20	7	141A	ARG	4.48	Phosphate	1909, 1909, 1912, 1908, 1910, 1911
Tro3-cTnI_20	8	145A	ARG	4.07	Phosphate	1890, 1890, 1889, 1891, 1892, 1893
Tro3-cTnI_20	9	145A	ARG	4.8	Phosphate	1869, 1869, 1868, 1872, 1870, 1871
Tro3-cTnI_20	10	146A	ARG	4.24	Phosphate	2369, 2369, 2368, 2370, 2371, 2372
Tro3-cTnI_20	11	146A	ARG	4.86	Phosphate	1869, 1869, 1868, 1872, 1870, 1871
Tro3-cTnI_20	12	148A	ARG	5.08	Phosphate	2389, 2389, 2388, 2392, 2390, 2391
Tro3-cTnI_20	13	148A	ARG	4.86	Phosphate	2369, 2369, 2368, 2370, 2371, 2372
Tro3-cTnI_20	14	148A	ARG	5.17	Phosphate	2350, 2350, 2352, 2353, 2349, 2351
Tro3-cTnI_20	15	164A	LYS	3.86	Phosphate	2168, 2168, 2169, 2170, 2171, 2167
Tro3-cTnI_20	16	164A	LYS	3.52	Phosphate	2188, 2188, 2187, 2189, 2190, 2191
Tro3-cTnI_20	17	170A	ARG	4.22	Phosphate	2209, 2209, 2208, 2210, 2211, 2212
Tro3-cTnI_20	18	170A	ARG	4.87	Phosphate	2229, 2229, 2232, 2228, 2230, 2231
Tro3-cTnI_20	19	174A	LYS	3.74	Phosphate	2229, 2229, 2232, 2228, 2230, 2231
Tro3-cTnI_40	1	21A	ARG	4.98	Phosphate	2229, 2229, 2232, 2228, 2230, 2231
Tro3-cTnI_40	2	21A	ARG	4.6	Phosphate	2248, 2248, 2247, 2249, 2250, 2251
Tro3-cTnI_40	3	38A	LYS	4.13	Phosphate	2005, 2005, 2004, 2008, 2006, 2007
Tro3-cTnI_40	4	38A	LYS	4.79	Phosphate	1986, 1986, 1985, 1987, 1988, 1989
Tro3-cTnI_40	5	40A	LYS	3.71	Phosphate	2025, 2025, 2024, 2026, 2027, 2028
Tro3-cTnI_40	6	46A	LYS	3.57	Phosphate	2025, 2025, 2024, 2026, 2027, 2028
Tro3-cTnI_40	7	50A	LYS	3.96	Phosphate	2408, 2408, 2409, 2410, 2411, 2407
Tro3-cTnI_40	8	127A	ASP	4.37	Guanidine	1774, 1775, 1777
Tro3-cTnI_40	9	131A	LYS	5.31	Phosphate	1745, 1745, 1744, 1746, 1747, 1748
Tro3-cTnI_40	10	140A	LYS	3.8	Phosphate	2470, 2470, 2472, 2473, 2469, 2471
Tro3-cTnI_40	11	141A	ARG	4.61	Phosphate	1909, 1909, 1912, 1908, 1910, 1911
Tro3-cTnI_40	12	145A	ARG	4.3	Phosphate	1869, 1869, 1868, 1872, 1870, 1871
Tro3-cTnI_40	13	145A	ARG	4.33	Phosphate	1890, 1890, 1889, 1891, 1892, 1893
Tro3-cTnI_40	14	146A	ARG	4.62	Phosphate	2369, 2369, 2368, 2370, 2371, 2372
Tro3-cTnI_40	15	146A	ARG	4.99	Phosphate	1869, 1869, 1868, 1872, 1870, 1871
Tro3-cTnI_40	16	148A	ARG	5.15	Phosphate	2389, 2389, 2388, 2392, 2390, 2391
Tro3-cTnI_40	17	148A	ARG	4.43	Phosphate	2369, 2369, 2368, 2370, 2371, 2372
Tro3-cTnI_40	18	164A	LYS	4.84	Phosphate	2188, 2188, 2187, 2189, 2190, 2191
Tro3-cTnI_40	19	164A	LYS	4.34	Phosphate	2168, 2168, 2169, 2170, 2171, 2167
Tro3-cTnI_40	20	170A	ARG	4.84	Phosphate	2209, 2209, 2208, 2210, 2211, 2212
Tro3-cTnI_40	21	174A	LYS	3.59	Phosphate	2229, 2229, 2232, 2228, 2230, 2231
Tro3-cTnI_60	1	21A	ARG	4.56	Phosphate	2248, 2248, 2247, 2249, 2250, 2251
Tro3-cTnI_60	2	38A	LYS	4.08	Phosphate	2005, 2005, 2008, 2004, 2006, 2007
Tro3-cTnI_60	3	46A	LYS	3.93	Phosphate	2025, 2025, 2024, 2026, 2027, 2028

Complex_Time point	Index	Residue	AA	Distance	Ligand Group	Ligand Atoms
Tro3-cTnI_60	4	50A	LYS	4.15	Phosphate	2408, 2408, 2409, 2410, 2411, 2407
Tro3-cTnI_60	5	127A	ASP	4.38	Guanidine	1774, 1775, 1777
Tro3-cTnI_60	6	141A	ARG	4.37	Phosphate	1909, 1909, 1912, 1908, 1910, 1911
Tro3-cTnI_60	7	145A	ARG	4.14	Phosphate	1890, 1890, 1889, 1891, 1892, 1893
Tro3-cTnI_60	8	145A	ARG	4.84	Phosphate	1869, 1869, 1868, 1872, 1870, 1871
Tro3-cTnI_60	9	146A	ARG	5.06	Phosphate	1869, 1869, 1868, 1872, 1870, 1871
Tro3-cTnI_60	10	146A	ARG	4.34	Phosphate	2369, 2369, 2368, 2370, 2371, 2372
Tro3-cTnI_60	11	148A	ARG	4.68	Phosphate	2369, 2369, 2368, 2370, 2371, 2372
Tro3-cTnI_60	12	148A	ARG	5.32	Phosphate	2389, 2389, 2388, 2392, 2390, 2391
Tro3-cTnI_60	13	164A	LYS	3.42	Phosphate	2188, 2188, 2187, 2189, 2190, 2191
Tro3-cTnI_60	14	164A	LYS	4.01	Phosphate	2168, 2168, 2169, 2170, 2171, 2167
Tro3-cTnI_60	15	170A	ARG	5.22	Phosphate	2229, 2229, 2232, 2228, 2230, 2231
Tro3-cTnI_60	16	170A	ARG	4.44	Phosphate	2209, 2209, 2208, 2210, 2211, 2212
Tro3-cTnI_60	17	174A	LYS	4.14	Phosphate	2229, 2229, 2232, 2228, 2230, 2231
Tro3-cTnI_80	1	38A	LYS	4.01	Phosphate	1986, 1986, 1985, 1987, 1988, 1989
Tro3-cTnI_80	2	38A	LYS	3.94	Phosphate	2005, 2005, 2004, 2008, 2006, 2007
Tro3-cTnI_80	3	58A	LYS	5.38	Phosphate	2209, 2209, 2208, 2210, 2211, 2212
Tro3-cTnI_80	4	69A	ARG	3.85	Phosphate	2269, 2269, 2268, 2272, 2270, 2271
Tro3-cTnI_80	5	127A	ASP	3.89	Guanidine	1774, 1775, 1777
Tro3-cTnI_80	6	131A	LYS	3.73	Phosphate	1745, 1745, 1744, 1746, 1747, 1748
Tro3-cTnI_80	7	141A	ARG	4.34	Phosphate	1909, 1909, 1912, 1908, 1910, 1911
Tro3-cTnI_80	8	145A	ARG	4.16	Phosphate	1869, 1869, 1872, 1868, 1870, 1871
Tro3-cTnI_80	9	145A	ARG	4.19	Phosphate	1890, 1890, 1889, 1891, 1892, 1893
Tro3-cTnI_80	10	146A	ARG	4.54	Phosphate	2369, 2369, 2368, 2370, 2371, 2372
Tro3-cTnI_80	11	146A	ARG	5.13	Phosphate	1869, 1869, 1872, 1868, 1870, 1871
Tro3-cTnI_80	12	148A	ARG	4.42	Phosphate	2369, 2369, 2368, 2370, 2371, 2372
Tro3-cTnI_80	13	148A	ARG	4.69	Phosphate	2389, 2389, 2388, 2392, 2390, 2391
Tro3-cTnI_80	14	164A	LYS	3.98	Phosphate	2188, 2188, 2187, 2189, 2190, 2191
Tro3-cTnI_80	15	164A	LYS	3.87	Phosphate	2168, 2168, 2169, 2170, 2171, 2167
Tro3-cTnI_80	16	170A	ARG	5.1	Phosphate	2229, 2229, 2228, 2232, 2230, 2231
Tro3-cTnI_80	17	170A	ARG	4.66	Phosphate	2209, 2209, 2208, 2210, 2211, 2212
Tro3-cTnI_80	18	174A	LYS	3.58	Phosphate	2229, 2229, 2228, 2232, 2230, 2231
Tro3-cTnI_100	1	21A	ARG	4.86	Phosphate	2248, 2248, 2247, 2249, 2250, 2251
Tro3-cTnI_100	2	21A	ARG	4.89	Phosphate	2229, 2229, 2228, 2232, 2230, 2231
Tro3-cTnI_100	3	37A	LYS	4.74	Phosphate	2448, 2448, 2449, 2450, 2451, 2447
Tro3-cTnI_100	4	38A	LYS	5.06	Phosphate	2470, 2470, 2469, 2472, 2473, 2471
Tro3-cTnI_100	5	40A	LYS	4.05	Phosphate	2025, 2025, 2024, 2026, 2027, 2028
Tro3-cTnI_100	6	68A	ARG	4.72	Phosphate	2288, 2288, 2289, 2290, 2291, 2287
Tro3-cTnI_100	7	69A	ARG	3.99	Phosphate	2269, 2269, 2268, 2272, 2270, 2271
Tro3-cTnI_100	8	127A	ASP	4.32	Guanidine	1774, 1775, 1777
Tro3-cTnI_100	9	141A	ARG	4.23	Phosphate	1909, 1909, 1908, 1912, 1910, 1911
Tro3-cTnI_100	10	145A	ARG	4.25	Phosphate	1869, 1869, 1872, 1868, 1870, 1871
Tro3-cTnI_100	11	145A	ARG	4.24	Phosphate	1890, 1890, 1889, 1891, 1892, 1893
Tro3-cTnI_100	12	146A	ARG	4.87	Phosphate	2369, 2369, 2368, 2370, 2371, 2372

Complex_Time point	Index	Residue	AA	Distance	Ligand Group	Ligand Atoms
Tro3-cTnI_100	13	146A	ARG	4.86	Phosphate	1869, 1869, 1872, 1868, 1870, 1871
Tro3-cTnI_100	14	148A	ARG	4.58	Phosphate	2389, 2389, 2392, 2388, 2390, 2391
Tro3-cTnI_100	15	148A	ARG	4.6	Phosphate	2369, 2369, 2368, 2370, 2371, 2372
Tro3-cTnI_100	16	164A	LYS	3.58	Phosphate	2188, 2188, 2187, 2189, 2190, 2191
Tro3-cTnI_100	17	164A	LYS	3.89	Phosphate	2168, 2168, 2167, 2169, 2170, 2171
Tro3-cTnI_100	18	170A	ARG	4.49	Phosphate	2209, 2209, 2208, 2210, 2211, 2212
Tro3-cTnI_0	1	40A	LYS	4.23	Phosphate	3297, 3297, 3296, 3298, 3299, 3300
Tro3-cTnI_0	2	46A	LYS	4.06	Phosphate	3297, 3297, 3296, 3298, 3299, 3300
Tro3-cTnI_0	3	141A	ARG	4.45	Phosphate	2485, 2485, 2488, 2484, 2486, 2487
Tro3-cTnI_0	4	164A	LYS	4.8	Phosphate	2923, 2923, 2922, 2924, 2925, 2926
Tro5-cTnI_0	1	46A	LYS	5.1	Phosphate	2700, 2700, 2699, 2701, 2702, 2703
Tro5-cTnI_0	2	46A	LYS	3.12	Phosphate	2670, 2670, 2672, 2673, 2669, 2671
Tro5-cTnI_0	3	50A	LYS	3.6	Phosphate	2638, 2638, 2637, 2640, 2641, 2639
Tro5-cTnI_0	4	148A	ARG	5.44	Phosphate	2578, 2578, 2577, 2579, 2580, 2581
Tro5-cTnI_0	5	148A	ARG	4.36	Phosphate	2608, 2608, 2609, 2610, 2611, 2607
Tro5-cTnI_0	6	164A	LYS	5.01	Phosphate	3198, 3198, 3200, 3201, 3197, 3199
Tro5-cTnI_0	7	204A	ARG	4.35	Phosphate	3168, 3168, 3167, 3169, 3170, 3171
Tro5-cTnI_20	1	38A	LYS	5.45	Phosphate	2084, 2084, 2083, 2085, 2086, 2087
Tro5-cTnI_20	2	40A	LYS	3.79	Phosphate	2084, 2084, 2083, 2085, 2086, 2087
Tro5-cTnI_20	3	46A	LYS	4.03	Phosphate	2045, 2045, 2048, 2044, 2046, 2047
Tro5-cTnI_20	4	50A	LYS	3.88	Phosphate	2006, 2006, 2008, 2009, 2005, 2007
Tro5-cTnI_20	5	50A	LYS	3.52	Phosphate	2026, 2026, 2025, 2027, 2028, 2029
Tro5-cTnI_20	6	140A	LYS	3.85	Phosphate	2166, 2166, 2168, 2169, 2165, 2167
Tro5-cTnI_20	7	207A	LYS	4.72	Phosphate	2325, 2325, 2324, 2328, 2326, 2327
Tro5-cTnI_40	1	38A	LYS	4.05	Phosphate	2084, 2084, 2083, 2085, 2086, 2087
Tro5-cTnI_40	2	40A	LYS	5.29	Phosphate	2084, 2084, 2083, 2085, 2086, 2087
Tro5-cTnI_40	3	46A	LYS	3.94	Phosphate	2026, 2026, 2025, 2027, 2028, 2029
Tro5-cTnI_40	4	50A	LYS	3.81	Phosphate	2006, 2006, 2005, 2008, 2009, 2007
Tro5-cTnI_40	5	50A	LYS	3.87	Phosphate	2026, 2026, 2025, 2027, 2028, 2029
Tro5-cTnI_40	6	138A	LYS	3.7	Phosphate	2125, 2125, 2128, 2124, 2126, 2127
Tro5-cTnI_40	7	140A	LYS	4	Phosphate	1968, 1968, 1969, 1970, 1971, 1967
Tro5-cTnI_40	8	140A	LYS	3.98	Phosphate	2146, 2146, 2145, 2147, 2148, 2149
Tro5-cTnI_40	9	146A	ARG	4.77	Phosphate	1949, 1949, 1948, 1952, 1950, 1951
Tro5-cTnI_40	10	146A	ARG	4.28	Phosphate	2185, 2185, 2184, 2186, 2187, 2188
Tro5-cTnI_40	11	204A	ARG	5.03	Phosphate	2346, 2346, 2345, 2347, 2348, 2349
Tro5-cTnI_40	12	204A	ARG	4.16	Phosphate	2325, 2325, 2328, 2324, 2326, 2327
Tro5-cTnI_40	13	205A	LYS	3.6	Phosphate	2365, 2365, 2364, 2368, 2366, 2367
Tro5-cTnI_40	14	207A	LYS	4.06	Phosphate	2346, 2346, 2345, 2347, 2348, 2349
Tro5-cTnI_40	15	207A	LYS	5.1	Phosphate	2365, 2365, 2364, 2368, 2366, 2367
Tro5-cTnI_60	1	37A	LYS	5.32	Phosphate	2084, 2084, 2083, 2085, 2086, 2087
Tro5-cTnI_60	2	37A	LYS	3.87	Phosphate	2103, 2103, 2102, 2104, 2105, 2106
Tro5-cTnI_60	3	38A	LYS	3.83	Phosphate	2084, 2084, 2083, 2085, 2086, 2087
Tro5-cTnI_60	4	46A	LYS	4.05	Phosphate	2026, 2026, 2025, 2027, 2028, 2029
Tro5-cTnI_60	5	50A	LYS	3.45	Phosphate	2026, 2026, 2025, 2027, 2028, 2029

Complex_Time point	Index	Residue	AA	Distance	Ligand Group	Ligand Atoms
Tro5-cTnI_60	6	50A	LYS	3.82	Phosphate	2006, 2006, 2008, 2009, 2005, 2007
Tro5-cTnI_60	7	138A	LYS	3.83	Phosphate	2125, 2125, 2128, 2124, 2126, 2127
Tro5-cTnI_60	8	140A	LYS	5.44	Phosphate	2146, 2146, 2145, 2147, 2148, 2149
Tro5-cTnI_60	9	140A	LYS	5.04	Phosphate	1968, 1968, 1969, 1970, 1971, 1967
Tro5-cTnI_60	10	146A	ARG	4.38	Phosphate	2185, 2185, 2184, 2186, 2187, 2188
Tro5-cTnI_60	11	146A	ARG	4.82	Phosphate	1949, 1949, 1948, 1952, 1950, 1951
Tro5-cTnI_60	12	162A	ARG	4.48	Phosphate	1743, 1743, 1742, 1744, 1745, 1746
Tro5-cTnI_60	13	164A	LYS	5.22	Phosphate	2365, 2365, 2368, 2364, 2366, 2367
Tro5-cTnI_60	14	204A	ARG	4.24	Phosphate	2346, 2346, 2345, 2347, 2348, 2349
Tro5-cTnI_60	15	204A	ARG	4.6	Phosphate	2325, 2325, 2328, 2324, 2326, 2327
Tro5-cTnI_60	16	205A	LYS	3.97	Phosphate	2365, 2365, 2368, 2364, 2366, 2367
Tro5-cTnI_60	17	207A	LYS	3.83	Phosphate	2346, 2346, 2345, 2347, 2348, 2349
Tro5-cTnI_80	1	20A	ARG	4.05	Phosphate	1701, 1701, 1704, 1700, 1702, 1703
Tro5-cTnI_80	2	40A	LYS	3.63	Phosphate	2084, 2084, 2083, 2085, 2086, 2087
Tro5-cTnI_80	3	46A	LYS	3.63	Phosphate	2026, 2026, 2025, 2027, 2028, 2029
Tro5-cTnI_80	4	46A	LYS	5.47	Phosphate	2045, 2045, 2048, 2044, 2046, 2047
Tro5-cTnI_80	5	50A	LYS	4.15	Phosphate	2006, 2006, 2005, 2008, 2009, 2007
Tro5-cTnI_80	6	50A	LYS	3.95	Phosphate	2026, 2026, 2025, 2027, 2028, 2029
Tro5-cTnI_80	7	138A	LYS	4.11	Phosphate	2125, 2125, 2124, 2128, 2126, 2127
Tro5-cTnI_80	8	140A	LYS	4.17	Phosphate	2146, 2146, 2145, 2147, 2148, 2149
Tro5-cTnI_80	9	140A	LYS	4.11	Phosphate	1968, 1968, 1967, 1969, 1970, 1971
Tro5-cTnI_80	10	141A	ARG	4.89	Phosphate	2166, 2166, 2165, 2168, 2169, 2167
Tro5-cTnI_80	11	146A	ARG	4.12	Phosphate	2185, 2185, 2184, 2186, 2187, 2188
Tro5-cTnI_80	12	146A	ARG	4.89	Phosphate	1949, 1949, 1952, 1948, 1950, 1951
Tro5-cTnI_80	13	162A	ARG	4.85	Phosphate	1782, 1782, 1784, 1785, 1781, 1783
Tro5-cTnI_80	14	162A	ARG	4.67	Phosphate	1762, 1762, 1761, 1763, 1764, 1765
Tro5-cTnI_80	15	164A	LYS	4.78	Phosphate	2365, 2365, 2364, 2368, 2366, 2367
Tro5-cTnI_80	16	204A	ARG	4.17	Phosphate	2346, 2346, 2345, 2347, 2348, 2349
Tro5-cTnI_80	17	204A	ARG	4.43	Phosphate	2325, 2325, 2328, 2324, 2326, 2327
Tro5-cTnI_80	18	205A	LYS	3.9	Phosphate	2365, 2365, 2364, 2368, 2366, 2367
Tro5-cTnI_80	19	207A	LYS	3.69	Phosphate	2346, 2346, 2345, 2347, 2348, 2349
Tro5-cTnI_100	1	13A	ARG	4.39	Phosphate	1762, 1762, 1761, 1763, 1764, 1765
Tro5-cTnI_100	2	13A	ARG	4.78	Phosphate	1743, 1743, 1744, 1745, 1746, 1742
Tro5-cTnI_100	3	20A	ARG	4.61	Phosphate	1701, 1701, 1700, 1704, 1702, 1703
Tro5-cTnI_100	4	21A	ARG	4.27	Phosphate	1701, 1701, 1700, 1704, 1702, 1703
Tro5-cTnI_100	5	40A	LYS	4.01	Phosphate	2064, 2064, 2063, 2065, 2066, 2067
Tro5-cTnI_100	6	46A	LYS	3.76	Phosphate	2045, 2045, 2048, 2044, 2046, 2047
Tro5-cTnI_100	7	50A	LYS	3.8	Phosphate	2006, 2006, 2005, 2008, 2009, 2007
Tro5-cTnI_100	8	50A	LYS	3.7	Phosphate	2026, 2026, 2025, 2027, 2028, 2029
Tro5-cTnI_100	9	140A	LYS	3.77	Phosphate	2146, 2146, 2145, 2147, 2148, 2149
Tro5-cTnI_100	10	140A	LYS	4.46	Phosphate	1968, 1968, 1967, 1969, 1970, 1971
Tro5-cTnI_100	11	146A	ARG	4.38	Phosphate	2185, 2185, 2184, 2186, 2187, 2188
Tro5-cTnI_100	12	146A	ARG	4.67	Phosphate	1949, 1949, 1952, 1948, 1950, 1951
Tro5-cTnI_100	13	162A	ARG	4.99	Phosphate	1762, 1762, 1761, 1763, 1764, 1765

Complex_Time point	Index	Residue	AA	Distance	Ligand Group	Ligand Atoms
Tro5-cTnI_100	14	204A	ARG	5.21	Phosphate	2346, 2346, 2345, 2347, 2348, 2349
Tro5-cTnI_100	15	204A	ARG	4.59	Phosphate	2325, 2325, 2324, 2328, 2326, 2327
Tro5-cTnI_100	16	205A	LYS	3.94	Phosphate	2365, 2365, 2364, 2368, 2366, 2367
Tro5-cTnI_100	17	207A	LYS	4.13	Phosphate	2346, 2346, 2345, 2347, 2348, 2349
Tro6-cTnI_0	1	50A	LYS	4.08	Phosphate	2510, 2510, 2509, 2512, 2513, 2511
Tro6-cTnI_0	2	146A	ARG	4.22	Phosphate	2575, 2575, 2574, 2576, 2577, 2578
Tro6-cTnI_0	3	146A	ARG	3.94	Phosphate	2416, 2416, 2417, 2418, 2419, 2415
Tro6-cTnI_0	4	148A	ARG	5.01	Phosphate	2575, 2575, 2574, 2576, 2577, 2578
Tro6-cTnI_0	5	148A	ARG	5.4	Phosphate	2543, 2543, 2544, 2545, 2546, 2542
Tro6-cTnI_0	6	207A	LYS	3.66	Phosphate	2762, 2762, 2761, 2763, 2764, 2765
Tro6-cTnI_20	1	36A	LYS	3.6	Phosphate	1925, 1925, 1924, 1928, 1926, 1927
Tro6-cTnI_20	2	37A	LYS	3.72	Phosphate	1947, 1947, 1946, 1948, 1949, 1950
Tro6-cTnI_20	3	50A	LYS	4.01	Phosphate	1987, 1987, 1986, 1988, 1989, 1990
Tro6-cTnI_20	4	50A	LYS	4.78	Phosphate	1967, 1967, 1968, 1969, 1970, 1966
Tro6-cTnI_20	5	141A	ARG	3.96	Phosphate	1805, 1805, 1808, 1804, 1806, 1807
Tro6-cTnI_20	6	146A	ARG	4.98	Phosphate	1864, 1864, 1863, 1865, 1866, 1867
Tro6-cTnI_20	7	148A	ARG	4.23	Phosphate	1864, 1864, 1863, 1865, 1866, 1867
Tro6-cTnI_20	8	183A	LYS	4.89	Phosphate	2148, 2148, 2147, 2149, 2150, 2151
Tro6-cTnI_20	9	193A	LYS	3.61	Phosphate	2148, 2148, 2147, 2149, 2150, 2151
Tro6-cTnI_20	10	204A	ARG	4.64	Phosphate	2107, 2107, 2106, 2108, 2109, 2110
Tro6-cTnI_20	11	204A	ARG	4.62	Phosphate	2086, 2086, 2088, 2089, 2085, 2087
Tro6-cTnI_20	12	206A	LYS	4.04	Phosphate	2127, 2127, 2126, 2128, 2129, 2130
Tro6-cTnI_40	1	37A	LYS	3.76	Phosphate	1947, 1947, 1946, 1948, 1949, 1950
Tro6-cTnI_40	2	37A	LYS	5.34	Phosphate	1906, 1906, 1905, 1907, 1908, 1909
Tro6-cTnI_40	3	45A	ARG	4.7	Phosphate	2385, 2385, 2384, 2386, 2387, 2388
Tro6-cTnI_40	4	136A	ARG	4.44	Phosphate	1885, 1885, 1888, 1884, 1886, 1887
Tro6-cTnI_40	5	141A	ARG	4.4	Phosphate	1805, 1805, 1808, 1804, 1806, 1807
Tro6-cTnI_40	6	141A	ARG	3.98	Phosphate	1843, 1843, 1842, 1844, 1845, 1846
Tro6-cTnI_40	7	148A	ARG	4.2	Phosphate	1967, 1967, 1966, 1968, 1969, 1970
Tro6-cTnI_40	8	148A	ARG	4.91	Phosphate	1864, 1864, 1863, 1865, 1866, 1867
Tro6-cTnI_40	9	192A	ARG	4.32	Phosphate	2190, 2190, 2192, 2193, 2189, 2191
Tro6-cTnI_40	10	192A	ARG	4.93	Phosphate	2170, 2170, 2169, 2171, 2172, 2173
Tro6-cTnI_40	11	193A	LYS	3.81	Phosphate	2148, 2148, 2147, 2149, 2150, 2151
Tro6-cTnI_40	12	204A	ARG	4.54	Phosphate	2107, 2107, 2106, 2108, 2109, 2110
Tro6-cTnI_40	13	204A	ARG	4.54	Phosphate	2067, 2067, 2066, 2068, 2069, 2070
Tro6-cTnI_40	14	206A	LYS	4.14	Phosphate	2127, 2127, 2128, 2129, 2130, 2126
Tro6-cTnI_40	15	207A	LYS	3.75	Phosphate	2086, 2086, 2088, 2089, 2085, 2087
Tro6-cTnI_80	1	21A	ARG	4.74	Phosphate	2344, 2344, 2345, 2346, 2347, 2343
Tro6-cTnI_80	2	22A	ARG	4.22	Phosphate	2344, 2344, 2345, 2346, 2347, 2343
Tro6-cTnI_80	3	37A	LYS	3.99	Phosphate	1947, 1947, 1946, 1948, 1949, 1950
Tro6-cTnI_80	4	45A	ARG	4.98	Phosphate	2405, 2405, 2408, 2404, 2406, 2407
Tro6-cTnI_80	5	136A	ARG	4.35	Phosphate	1885, 1885, 1888, 1884, 1886, 1887
Tro6-cTnI_80	6	141A	ARG	4.66	Phosphate	1805, 1805, 1808, 1804, 1806, 1807
Tro6-cTnI_80	7	141A	ARG	4.17	Phosphate	1843, 1843, 1842, 1844, 1845, 1846

Complex_Time point	Index	Residue	AA	Distance	Ligand Group	Ligand Atoms
Tro6-cTnI_80	8	146A	ARG	5.13	Phosphate	1783, 1783, 1784, 1785, 1786, 1782
Tro6-cTnI_80	9	148A	ARG	4.32	Phosphate	1967, 1967, 1968, 1969, 1970, 1966
Tro6-cTnI_80	10	148A	ARG	4.69	Phosphate	1864, 1864, 1865, 1866, 1867, 1863
Tro6-cTnI_80	11	172A	HIS	4.85	Phosphate	2127, 2127, 2126, 2128, 2129, 2130
Tro6-cTnI_80	12	192A	ARG	4.97	Phosphate	2190, 2190, 2189, 2192, 2193, 2191
Tro6-cTnI_80	13	192A	ARG	4.5	Phosphate	2229, 2229, 2232, 2228, 2230, 2231
Tro6-cTnI_80	14	193A	LYS	3.95	Phosphate	2148, 2148, 2147, 2149, 2150, 2151
Tro6-cTnI_80	15	204A	ARG	5.11	Phosphate	2086, 2086, 2088, 2089, 2085, 2087
Tro6-cTnI_80	16	204A	ARG	4.52	Phosphate	2067, 2067, 2066, 2068, 2069, 2070
Tro6-cTnI_80	17	206A	LYS	3.71	Phosphate	2127, 2127, 2126, 2128, 2129, 2130
Tro6-cTnI_80	18	206A	LYS	5.01	Phosphate	2107, 2107, 2106, 2108, 2109, 2110
Tro6-cTnI_80	19	207A	LYS	5.24	Phosphate	2086, 2086, 2088, 2089, 2085, 2087
Tro6-cTnI_60	1	22A	ARG	4.91	Phosphate	2344, 2344, 2345, 2346, 2347, 2343
Tro6-cTnI_60	2	37A	LYS	3.89	Phosphate	1947, 1947, 1946, 1948, 1949, 1950
Tro6-cTnI_60	3	40A	LYS	4	Phosphate	1701, 1701, 1700, 1704, 1702, 1703
Tro6-cTnI_60	4	45A	ARG	4.68	Phosphate	2405, 2405, 2404, 2408, 2406, 2407
Tro6-cTnI_60	5	50A	LYS	5.28	Phosphate	1967, 1967, 1968, 1969, 1970, 1966
Tro6-cTnI_60	6	136A	ARG	4.5	Phosphate	1885, 1885, 1888, 1884, 1886, 1887
Tro6-cTnI_60	7	141A	ARG	4.67	Phosphate	1805, 1805, 1804, 1808, 1806, 1807
Tro6-cTnI_60	8	141A	ARG	4.25	Phosphate	1843, 1843, 1842, 1844, 1845, 1846
Tro6-cTnI_60	9	146A	ARG	4.63	Phosphate	1987, 1987, 1986, 1988, 1989, 1990
Tro6-cTnI_60	10	148A	ARG	4.22	Phosphate	1967, 1967, 1968, 1969, 1970, 1966
Tro6-cTnI_60	11	148A	ARG	4.25	Phosphate	1864, 1864, 1863, 1865, 1866, 1867
Tro6-cTnI_60	12	172A	HIS	5.02	Phosphate	2127, 2127, 2126, 2128, 2129, 2130
Tro6-cTnI_60	13	193A	LYS	5.3	Phosphate	2148, 2148, 2147, 2149, 2150, 2151
Tro6-cTnI_60	14	204A	ARG	5.15	Phosphate	2086, 2086, 2088, 2089, 2085, 2087
Tro6-cTnI_60	15	206A	LYS	3.91	Phosphate	2127, 2127, 2126, 2128, 2129, 2130
Tro6-cTnI_60	16	207A	LYS	3.82	Phosphate	2086, 2086, 2088, 2089, 2085, 2087
Tro6-cTnI_100	1	21A	ARG	5.41	Phosphate	2344, 2344, 2343, 2345, 2346, 2347
Tro6-cTnI_100	2	22A	ARG	5.13	Phosphate	2385, 2385, 2384, 2386, 2387, 2388
Tro6-cTnI_100	3	37A	LYS	4.02	Phosphate	1947, 1947, 1946, 1948, 1949, 1950
Tro6-cTnI_100	4	37A	LYS	5.49	Phosphate	1906, 1906, 1905, 1907, 1908, 1909
Tro6-cTnI_100	5	40A	LYS	4.72	Phosphate	2466, 2466, 2465, 2467, 2468, 2469
Tro6-cTnI_100	6	45A	ARG	4.05	Phosphate	2405, 2405, 2404, 2408, 2406, 2407
Tro6-cTnI_100	7	45A	ARG	4.91	Phosphate	2427, 2427, 2426, 2428, 2429, 2430
Tro6-cTnI_100	8	136A	ARG	4.89	Phosphate	1885, 1885, 1884, 1888, 1886, 1887
Tro6-cTnI_100	9	141A	ARG	4.36	Phosphate	1805, 1805, 1808, 1804, 1806, 1807
Tro6-cTnI_100	10	148A	ARG	4.46	Phosphate	1967, 1967, 1966, 1968, 1969, 1970
Tro6-cTnI_100	11	148A	ARG	4.1	Phosphate	1864, 1864, 1865, 1866, 1867, 1863
Tro6-cTnI_100	12	192A	ARG	4.49	Phosphate	2229, 2229, 2232, 2228, 2230, 2231
Tro6-cTnI_100	13	192A	ARG	4.59	Phosphate	2190, 2190, 2192, 2193, 2189, 2191
Tro6-cTnI_100	14	192A	ARG	4.92	Phosphate	2248, 2248, 2249, 2250, 2251, 2247
Tro6-cTnI_100	15	204A	ARG	5.1	Phosphate	2086, 2086, 2085, 2088, 2089, 2087
Tro6-cTnI_100	16	204A	ARG	4.11	Phosphate	2067, 2067, 2066, 2068, 2069, 2070

Complex_Time point	Index	Residue	AA	Distance	Ligand Group	Ligand Atoms
Tro6-cTnI_100	17	204A	ARG	4.82	Phosphate	2047, 2047, 2048, 2049, 2050, 2046
Tro6-cTnI_100	18	206A	LYS	5.11	Phosphate	2127, 2127, 2126, 2128, 2129, 2130
Tro6-cTnI_100	19	206A	LYS	3.83	Phosphate	2107, 2107, 2106, 2108, 2109, 2110

Table A.6. Atom Types in Chimera [50]

IDATM atom type		Description
Chimera	Original	
C3	C3	*sp*^{3}-hybridized carbon
C2	C2	*sp*^{2}-hybridized carbon
Car	C2	aromatic carbon
Cac	Cac	carboxylate carbon
C1	C1	*sp*-hybridized carbon
C1–	C1	*sp*-hybridized carbon with formal negative charge (carbon monoxide)
N3+	N3+, Nox	*sp*^{3}-hybridized nitrogen with formal positive charge
N3	N3	*sp*^{3}-hybridized nitrogen, formally neutral
N2+	Npl	*sp*^{2}-hybridized ring nitrogen bonded to three other atoms, formally positive
N2	Npl	*sp*^{2}-hybridized nitrogen bonded to two other atoms, formally neutral (pyridine)
Npl	Npl	*sp*^{2}-hybridized nitrogen bonded to three other atoms, formally neutral (amide, aniline)
Ng+	Ng+	resonance-equivalent nitrogen sharing formal positive charge (guanidinium, amidinium)
Ntr	Ntr	nitro group nitrogen
N1+	N1	*sp*-hybridized nitrogen bonded to two other atoms
N1	N1	*sp*-hybridized nitrogen
O3	O3	*sp*^{3}-hybridized oxygen
O2	O2	*sp*^{2}-hybridized oxygen
Oar+	(none)	aromatic oxygen, formally positive (pyrylium)
Oar	(none)	aromatic oxygen, formally neutral
O3–	O–	possibly resonance-equivalent terminal oxygen on tetrahedral center (phosphate, sulfate, N-oxide)
O2–	O–	resonance-equivalent terminal oxygen on planar center (carboxylate, nitro, nitrate)
O1+	(none)	*sp*-hybridized oxygen with formal positive charge (carbon monoxide)
O1	(none)	*sp*-hybridized oxygen (nitric oxide)
S3+	S3+	*sp*^{3}-hybridized sulfur with formal positive charge
S3	S3	*sp*^{3}-hybridized sulfur
S2	S2	*sp*^{2}-hybridized sulfur
Sar	(none)	aromatic sulfur
S3–	S2	terminal sulfur on tetrahedral center (thiophosphate)
Sac	Sac	sulfate, sulfonate, or sulfamate sulfur
Son	Sox	sulfone sulfur (>SO_2)
Sxd	Sox	sulfoxide sulfur (>SO)
S	S	other sulfur

IDATM atom type		Description
Chimera	Original	
B	Bac, Box, B	boron
P3+	P3+	*sp*[3]-hybridized phosphorus with formal positive charge
Pac	Pac	phosphate, phosphonate, or phosphamate phosphorus
Pox	Pox	P-oxide phosphorus
P	P	other phosphorus
HC	HC	hydrogen bonded to carbon
H	H	other hydrogen
DC	DC	deuterium bonded to carbon
D	D	other deuterium
(element symbol)	(element symbol)	atoms of elements not mentioned above

Reference

[50] Atom Types in Chimera. UCSF Computer Graphics Laboratory. (2012, June). URL https://www.cgl.ucsf.edu/chimera/docs/UsersGuide/idatm.html
