# Supplementary material for: The molecular interaction of six single-stranded DNA aptamers to cardiac troponin I revealed by docking and molecular dynamics simulation
Source: PLoS One. 2024 May 15;19(5):e0302475. doi: 10.1371/journal.pone.0302475 (PMC11095691; doi:10.1371/journal.pone.0302475)
Supplement: S2 File — (PDF) [file pone.0302475.s002.pdf]

**Table B.1.** Energy terms in MMPB (GB) SA calculation, GB (kcal/mol)

| Energyterms | Tro1-cTnI | Tro2-cTnI | Tro3-cTnI | Tro4-cTnI | Tro5-cTnI | Tro6-cTnI |
|---|---|---|---|---|---|---|
| ΔVDWAALS | -138.58 | -0.00 | -0.00 | --127.39 | -0.00 | -90.22 |
| ΔEEL | -7898.76 | -1659.49 | -1792.08 | -9759.04 | -1494.25 | -5809.56 |
| ΔEGB | 7923.96 | 1638.35 | 1769.26 | 9702.41 | 1475.22 | 5798.54 |
| ΔESURF | -18.82 | -0.00 | -0.00 | -18.45 | -0.00 | -12.65 |
| ΔGGAS | -8037.34 | -1659.49 | -1792.09 | -9886.43 | -1494.25 | -5899.78 |
| ΔGSOLV | 7905.14 | 1638.35 | 1769.26 | 9683.95 | 1475.22 | 5785.89 |
| ΔTOTAL | -132.20 | -21.14 | -22.83 | -202.47 | -19.04 | -113.89 |

**Table B.2.** Energy terms in MMPB (GB) SA calculation, PB (kcal/mol)

| Energyterms | Tro1-cTnI | Tro2-cTnI | Tro3-cTnI | Tro4-cTnI | Tro5-cTnI | Tro6-cTnI |
|---|---|---|---|---|---|---|
| ΔVDWAALS | -138.58 | -0.00 | -0.00 | -127.39 | -0.00 | -90.22 |
| ΔEEL | -7898.76 | -1659.49 | -1792.08 | -9759.04 | -1494.25 | -5809.56 |
| ΔEPB | 7894.62 | 1638.92 | 1770.20 | 9666.96 | 1476.65 | 5785.21 |
| ΔENPOLAR | -17.91 | -0.00 | -0.00 | -16.66 | -0.00 | -10.84 |
| ΔGGAS | -8037.34 | -1659.49 | -1792.09 | -9886.43 | -1494.25 | -5899.78 |
| ΔGSOLF | 7876.71 | 1638.92 | 1770.20 | 9650.30 | 1476.65 | 5774.37 |
| ΔTOTAL | -160.63 | -20.57 | -21.89 | -236.13 | -17.61 | -125.41 |
