# Supplementary material for: The molecular interaction of six single-stranded DNA aptamers to cardiac troponin I revealed by docking and molecular dynamics simulation
Source: PLoS One. 2024 May 15;19(5):e0302475. doi: 10.1371/journal.pone.0302475 (PMC11095691; doi:10.1371/journal.pone.0302475)
Supplement: S3 File — (PDF) [file pone.0302475.s003.pdf]

Table C.1. The number of molecular interactions of all aptamers with the protein

	1	2	3	4	5	6	7	8	9	10	11	12	13	14	15	16	17	18	19	20
	Met	Ala	Asp	Gly	Ser	Ser	Asp	Ala	Ala	Arg	Glu	Pro	Arg	Pro	Ala	Pro	Ala	Pro	Ile	Arg
Tro1	0	0	0	0	0	0	0	0	0	0	0	0	0	0	0	0	0	2	5	14
Tro2	0	0	0	0	0	0	0	0	0	0	0	0	0	0	0	0	0	0	0	0
Tro3	0	0	0	0	0	0	0	0	0	0	0	0	0	0	0	0	0	0	0	1
Tro4	1	0	0	0	2	2	0	0	0	11	2	0	0	0	0	0	0	0	0	0
Tro5	0	0	0	0	0	0	0	0	0	0	0	0	3	0	0	0	4	0	1	10
Tro6	0	0	0	0	0	0	0	0	0	0	0	0	0	0	0	0	0	0	2	0

	21	22	23	24	25	26	27	28	29	30	31	32	33	34	35	36	37	38	39	40
	Arg	Arg	Ser	Ser	Asn	Tyr	Arg	Ala	Tyr	Ala	Thr	Glu	Pro	His	Ala	Lys	Lys	Lys	Ser	Lys
Tro1	24	3	2	2	3	0	0	0	3	0	8	1	0	5	0	28	9	0	2	4
Tro2	0	0	0	0	0	0	0	0	0	0	0	0	0	1	3	6	17	3	2	0
Tro3	13	1	0	0	0	0	0	0	0	0	0	0	0	0	0	0	6	21	0	7
Tro4	0	0	0	0	0	0	0	0	0	0	0	0	0	1	2	3	18	8	0	8
Tro5	2	0	0	0	0	0	0	0	0	0	0	0	0	0	0	0	3	5	1	11
Tro6	5	5	0	0	0	0	0	0	0	0	0	1	0	0	0	5	16	1	4	6

	41	42	43	44	45	46	47	48	49	50	51	52	53	54	55	56	57	58	59	60
	Ile	Ser	Ala	Ser	Arg	Lys	Leu	Gln	Leu	Lys	Thr	Leu	Leu	Leu	Gln	Ile	Ala	Lys	Gln	Glu
Tro1	0	7	2	6	11	0	0	6	0	0	1	4	0	0	8	0	0	0	2	0
Tro2	0	0	5	2	0	7	6	1	0	12	0	0	0	0	0	0	0	0	0	0
Tro3	6	4	0	0	0	10	0	0	0	6	0	0	0	0	0	0	0	1	0	0
Tro4	0	0	0	0	0	6	0	0	0	7	0	0	0	0	0	0	0	0	0	0
Tro5	0	2	0	0	0	16	1	1	0	12	0	0	1	1	0	0	1	0	0	1
Tro6	1	7	2	0	11	9	0	0	0	4	0	0	0	0	0	0	0	0	0	0

	61	62	63	64	65	66	67	68	69	70	71	72	73	74	75	76	77	78	79	80
	Leu	Glu	Arg	Glu	Ala	Glu	Glu	Arg	Arg	Gly	Glu	Lys	Gly	Arg	Ala	Leu	Ser	Thr	Arg	Cys
Tro1	0	1	0	0	0	1	0	0	12	0	0	0	0	2	0	0	0	0	0	0
Tro2	0	0	0	0	0	0	0	0	0	0	0	0	0	0	0	0	0	0	0	0
Tro3	0	0	0	0	0	0	0	1	7	0	0	1	0	0	0	0	0	0	0	0
Tro4	0	0	0	0	0	0	0	0	0	0	0	0	0	0	0	0	0	0	0	0
Tro5	0	0	0	0	0	0	0	0	0	0	0	0	0	0	0	0	0	0	0	0
Tro6	0	0	0	0	0	0	0	0	0	0	0	0	0	0	0	0	0	0	0	0

	81	82	83	84	85	86	87	88	89	90	91	92	93	94	95	96	97	98	99	100
	Gln	Pro	Leu	Glu	Leu	Ala	Gly	Leu	Gly	Phe	Ala	Glu	Leu	Gln	Asp	Leu	Cys	Arg	Gln	Leu
Tro1	1	0	1	0	0	0	0	0	0	0	0	0	1	2	0	0	0	10	0	4
Tro2	0	0	0	0	0	0	0	0	0	0	0	0	0	0	0	0	0	0	0	0
Tro3	0	0	0	0	0	0	0	0	0	0	0	0	0	0	0	0	0	0	0	0
Tro4	0	0	0	0	0	0	0	0	0	0	0	0	0	0	0	0	0	0	0	0
Tro5	0	0	0	0	0	0	0	0	0	0	0	0	0	0	0	0	0	0	0	0
Tro6	0	0	0	0	0	0	0	0	0	0	0	0	0	0	0	0	0	0	0	0

	101	102	103	104	105	106	107	108	109	110	111	112	113	114	115	116	117	118	119	120
	His	Ala	Arg	Val	Asp	Lys	Val	Asp	Glu	Glu	Arg	Tyr	Asp	Ile	Glu	Ala	Lys	Val	Thr	Lys
Tro1	14	0	0	0	4	0	0	0	0	0	0	0	0	0	0	0	0	0	0	0
Tro2	0	0	0	0	0	0	0	0	0	0	0	0	0	0	0	0	0	0	0	0
Tro3	0	0	0	0	0	0	0	4	0	0	0	14	0	0	0	0	1	0	0	1
Tro4	0	0	0	0	0	0	0	0	0	0	0	0	0	0	0	0	0	0	0	0
Tro5	0	0	0	0	0	0	0	0	0	0	0	0	0	0	0	0	0	0	0	0
Tro6	0	0	0	0	0	0	0	0	0	0	0	0	0	0	0	0	0	0	0	0

	121	122	123	124	125	126	127	128	129	130	131	132	133	134	135	136	137	138	139	140
	Asn	Ile	Thr	Glu	Ile	Ala	Asp	Leu	Thr	Gln	Lys	Ile	Phe	Asp	Leu	Arg	Gly	Lys	Phe	Lys
Tro1	0	0	0	0	0	0	0	0	0	0	0	0	0	0	0	0	0	0	0	0
Tro2	0	0	0	0	0	0	0	0	0	0	0	0	0	0	0	4	0	0	0	5
Tro3	0	0	0	1	0	0	7	0	0	0	8	0	0	0	0	0	0	0	0	6
Tro4	0	0	0	0	0	0	0	0	0	0	0	0	0	0	0	0	0	0	12	8
Tro5	0	0	0	0	0	1	0	0	0	1	0	0	0	0	0	0	0	3	2	17
Tro6	0	0	0	0	0	0	0	0	0	0	0	0	0	0	0	7	4	4	11	0

	141	142	143	144	145	146	147	148	149	150	151	152	153	154	155	156	157	158	159	160
	Arg	Pro	Thr	Leu	Arg	Arg	Val	Arg	Ile	Ser	Ala	Asp	Ala	Met	Met	Gln	Ala	Leu	Leu	Gly
Tro1	1	0	0	0	0	0	0	1	0	0	0	0	0	0	0	0	0	0	0	0
Tro2	8	3	4	2	4	15	9	10	5	3	0	0	0	0	0	0	3	2	0	0
Tro3	13	1	3	0	18	26	0	11	2	1	0	0	1	0	0	1	1	1	0	0
Tro4	4	0	2	0	0	14	3	6	10	10	0	1	1	0	0	0	0	0	0	0
Tro5	4	0	2	0	0	23	9	7	7	7	0	1	2	1	0	2	0	1	0	0
Tro6	14	1	1	0	1	7	2	18	2	0	0	0	0	0	0	0	0	0	0	0

	161	162	163	164	165	166	167	168	169	170	171	172	173	174	175	176	177	178	179	180
	Ala	Arg	Ala	Lys	Glu	Ser	Leu	Asp	Leu	Arg	Ala	His	Leu	Lys	Gln	Val	Lys	Lys	Glu	Asp
Tro1	0	20	0	0	6	6	0	0	1	19	0	0	1	2	0	0	11	0	0	0
Tro2	3	14	0	0	0	0	0	0	0	0	0	0	0	0	1	0	0	1	0	0
Tro3	1	0	0	12	0	0	2	0	0	8	0	0	0	15	1	0	1	0	0	0
Tro4	0	0	0	0	0	0	0	2	0	1	0	0	0	0	0	0	0	0	0	0
Tro5	7	9	1	4	1	0	0	0	0	0	0	0	0	0	2	0	0	0	2	0
Tro6	2	0	0	0	0	0	0	0	0	0	0	3	0	0	2	0	0	0	1	0

	181	182	183	184	185	186	187	188	189	190	191	192	193	194	195	196	197	198	199	200
	Thr	Glu	Lys	Glu	Asn	Arg	Glu	Val	Gly	Asp	Trp	Arg	Lys	Asn	Ile	Asp	Ala	Leu	Ser	Gly
Tro1	1	0	0	0	0	4	0	0	0	3	2	9	1	0	0	0	0	0	0	0
Tro2	0	0	0	0	0	0	0	0	0	0	0	0	0	0	0	0	0	0	0	0
Tro3	0	0	0	0	0	0	0	0	0	0	6	0	0	0	0	0	0	0	0	0
Tro4	0	0	0	0	0	0	0	0	0	0	0	0	0	0	0	0	0	0	0	0
Tro5	0	0	0	0	0	0	0	0	0	0	0	0	0	0	0	0	0	0	0	0
Tro6	0	0	4	1	1	5	0	1	0	1	0	8	5	7	0	0	0	0	2	0

	201	202	203	204	205	206	207	208	209	210
	Met	Glu	Gly	Arg	Lys	Lys	Lys	Phe	Glu	Ser
Tro1	0	1	1	1	2	6	2	0	0	0
Tro2	0	2	2	17	5	2	9	2	1	2
Tro3	0	0	0	0	0	0	0	3	2	3
Tro4	0	0	0	15	1	0	6	1	2	2
Tro5	0	0	0	21	4	3	12	0	1	0
Tro6	0	2	2	14	5	8	8	1	5	1

Table C.2. The rank of total molecular interactions of all aptamers with the protein (>50)

Residue	Tro1	Tro2	Tro3	Tro4	Tro5	Tro6	Total
146	0	15	26	14	23	7	85
37	9	17	6	18	3	16	69
204	1	17	0	15	21	14	68
148	1	10	11	6	7	18	53
